# Mitochondrial DNA release via VDAC1 in keratinocytes: a key driver of innate immunity and vitiligo pathogenesis

**DOI:** 10.1038/s41419-026-08585-5

**Published:** 2026-03-18

**Authors:** Jinpeng Lv, Wenhui Xu, Peiwen Jiang, Wenhao Yu, Hui Xue, Nan Hu, Yan Cao, Huansha Zhang, Chuanwei Yin, Rongyin Gao

**Affiliations:** 1 Jiangsu Provincial Engineering Research Center for Drug Intelligent Manufacturing and Precision Delivery, School of Pharmacy, Changzhou University, Changzhou, China; 2 Department of Pharmacy, The First People’s Hospital of Changzhou, The Third Affiliated Hospital of Soochow University, Changzhou, China; 3 Department of Dermatology, The Second People’s Hospital of Changzhou, The Third Affiliated Hospital of Nanjing Medical University, Changzhou Medical Center, Nanjing Medical University, Changzhou, China

**Keywords:** Pharmacology, Innate immunity, Stress signalling, Autoimmune diseases

## Abstract

Vitiligo is an autoimmune depigmenting disorder in which oxidative stress is considered a critical trigger of innate immune activation. Although keratinocytes are increasingly recognized as key contributors to disease progression, the mechanism by which they sense and propagate oxidative stress signals has remained unclear. Here, we identify mitochondrial DNA (mtDNA) release as a pivotal event linking oxidative stress to immune activation in keratinocytes. We demonstrate that hydrogen peroxide induces a sequential mitochondrial membrane remodeling process, in which mitochondrial permeability transition pore opening precedes oligomerization of the outer membrane channel protein VDAC1, enabling selective mtDNA release under non-apoptotic conditions. Escaped mtDNA acts as a danger signal that concurrently activates the cGAS–STING axis and the NLRP3 inflammasome, driving type I and type II interferon production, chemokine release, and pyroptosis. Importantly, genetic silencing or pharmacological inhibition of VDAC1 with VBIT-4 effectively blocked mtDNA release, suppressed downstream inflammatory cascades, and alleviated depigmentation and CD8⁺ T cell infiltration in a murine vitiligo model. These findings uncover a previously unrecognized mechanism by which keratinocytes transform oxidative stress into autoimmune signaling and highlight VDAC1-dependent mtDNA release as a promising therapeutic target to intercept vitiligo at an early stage.

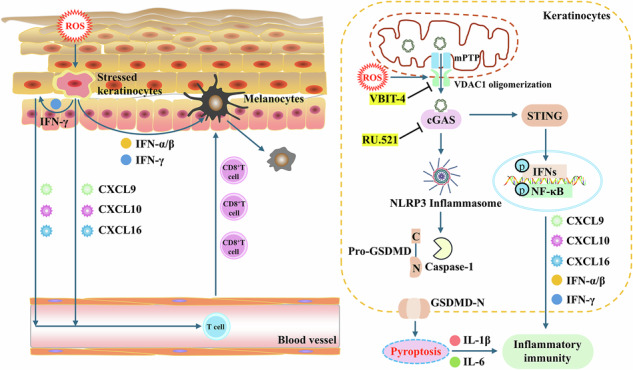

## Introduction

Vitiligo is a chronic autoimmune disorder characterized by progressive skin depigmentation, affecting 0.5–2% of the global population, and remains difficult to treat due to its multifactorial and incompletely understood pathogenesis [1, 2]. Among several proposed mechanisms, oxidative stress-induced innate immune activation is now widely recognized as a central event that precedes CD8⁺ T cell-mediated melanocyte destruction [2, 3]. Critically, keratinocytes-historically regarded as passive bystanders-are now recognized as active orchestrators of the cutaneous immune microenvironment [4–6]. Under oxidative stress, keratinocytes release a spectrum of pro-inflammatory cytokines and chemokines that orchestrate the recruitment and activation of cytotoxic T lymphocytes [7–10]. Therefore, identifying the key danger-associated molecular patterns (DAMPs) that convert oxidative stress into innate immune signaling in keratinocytes is crucial to advance our understanding and treatment of vitiligo.

Mitochondria are a major source of endogenous DAMPs, with mitochondrial DNA (mtDNA) being a particularly potent immunostimulatory signal in various pathological contexts [11, 12]. In vitiligo, both cytosolic and circulating mtDNA have been detected [13], linking mitochondrial oxidative stress to autoimmunity involving melanocytes and monocytes [14–16]. Intriguingly, clinical observations indicate that cytosolic mtDNA accumulation in vitiligo skin occurs mainly in keratinocytes rather than melanocytes or immune cells [16]. Given the numerical abundance of keratinocytes and their prominent role as epidermal immune sentinels, keratinocyte-derived mtDNA represents a compelling candidate linking oxidative stress to innate immune activation in vitiligo. Nevertheless, this possibility has not been directly tested, and the upstream triggers of mtDNA release as well as its downstream signaling consequences remain poorly defined.

Mitochondria are highly dynamic organelles with complex structures, and the release of mtDNA into the cytosol involves multiple proteins and channels that may vary depending on cell type and stress conditions [11]. In the classical apoptotic pathway, this process is initiated at the outer mitochondrial membrane (OMM) through BAX/BAK macropore formation, which subsequently enables inner mitochondrial membrane (IMM) herniation and mtDNA release [17, 18]. In contrast, under non-apoptotic oxidative stress, IMM permeabilization has been associated with opening of the mitochondrial permeability transition pore (mPTP), whereas outer membrane translocation has been linked to voltage-dependent anion channel 1 (VDAC1) oligomerization [19–22]. Consistent with this model, our previous work demonstrated that oxidative stress induces VDAC1-dependent mtDNA release in melanocytes [23]. However, whether mPTP opening and VDAC1 oligomerization function in a coordinated or sequential manner, and whether this mechanism operates similarly in keratinocytes, remain unresolved. Addressing this gap is particularly relevant to vitiligo, where oxidative stress-induced innate immune activation precedes melanocyte loss, raising the possibility that VDAC1-dependent mtDNA release in keratinocytes may link mitochondrial stress to inflammatory signaling and disease initiation.

Cytosolic mtDNA activates two major DNA-sensing pathways: the cyclic GMP-AMP synthase-stimulator of interferon genes (cGAS-STING) axis and the NOD-like receptor family pyrin domain-containing protein 3 (NLRP3) inflammasome [24]. Recognition of mtDNA by cGAS leads to STING activation, phosphorylation of TANK-binding kinase 1 (TBK1) and interferon regulatory factor 3 (IRF3), and the induction of type I interferons and NF-κB-driven proinflammatory mediators [25]. In parallel, mtDNA activates the NLRP3 inflammasome, which promotes caspase-1 cleavage, cytokine maturation, and pyroptotic cell death [26]. Given the dual immunostimulatory potential of mtDNA, we hypothesized that oxidative stress-induced mtDNA release in keratinocytes represents a key upstream event that couples mitochondrial dysfunction to autoimmune signaling in vitiligo.

To test this hypothesis, we investigated whether oxidative stress promotes mtDNA release in keratinocytes, delineated the molecular mechanism underlying this release, and determined how released mtDNA engages the cGAS-STING and NLRP3 inflammasome pathways to drive inflammatory responses. By uncovering the role of VDAC1-dependent mtDNA release, our study aims to establish a previously unrecognized mechanism by which keratinocytes actively shape the autoimmune microenvironment of vitiligo.

## Materials and methods

### Patients and specimens

Perilesional skin tissues were collected from patients with progressive-stage vitiligo at the Department of Dermatology, The Third Affiliated Hospital of Nanjing Medical University. Subjects who had received systemic or topical treatment within the three months prior to sample collection were excluded based on pre-established criteria. Control skin samples were obtained from age- and sex-matched individuals undergoing plastic surgery at the same institution. Written informed consents were obtained from all participants in accordance with the Declaration of Helsinki, and the study was approved by the Clinical Research Ethics Committee and the Ethics Review Board of The Third Affiliated Hospital of Nanjing Medical University (Approval No. [2024]KY029-01A). Skin samples intended for immunofluorescence analysis were fixed and paraffin-embedded.

### Animal models

Animal experiments were approved by the Animal Care and Use Committee of Changzhou University (Approval No: 202402280052-1) and conducted in accordance with its guidelines. Female C57BL/6 mice (5 weeks old, 20 ± 2 g) were purchased from Shanghai Bikai Keyi Biotechnology Co., Ltd and maintained under specific pathogen-free (SPF) conditions with a 12-h light/12-h dark cycle and free access to water and standard chow. Mice were randomly divided into three groups (*n* = 6 per group) using a computer-generated random number sequence: (1) PBS-treated control; (2) H_2_O_2_-induced vitiligo model; and (3) H_2_O_2_ + VBIT-4 treatment. After one week of acclimatization, dorsal hair in a 2 × 2 cm area was removed using electric clippers. Two days later, 5% (w/v) H_2_O_2_ was applied topically twice daily for 4 weeks to induce vitiligo-like lesions, while the control group received sterile PBS. After induction, mice in the H_2_O_2_ + VBIT-4 group were administered VBIT-4 intradermally (10 mg/kg, once daily) for 3 weeks. VBIT-4 (V413014, Aladdin, Shanghai, China) was dissolved in a vehicle consisting of 10% DMSO and 90% saline containing 20% (w/v) sulfobutylether-β-cyclodextrin (SBE-β-CD) to a final concentration of 4 mg/mL. The sample size (*n* = 6 per group) was not determined by a formal power analysis. Instead, it was chosen based on common practice in the field, preliminary data, and prior studies indicating that this group size is sufficient to detect biologically relevant effects. No animals were excluded from the analysis.

### Cell culture and treatment

The human immortalized keratinocyte HaCaT cell line was obtained from the Cell Bank of the Chinese Academy of Sciences. The cell line was authenticated by short tandem repeat (STR) profiling and confirmed to be free of mycoplasma contamination. HaCaT cells were cultured in Dulbecco’s Modified Eagle Medium (DMEM) supplemented with 10% fetal bovine serum and 1% penicillin/streptomycin. Normal human keratinocytes (NHKs) were isolated from foreskin specimens of healthy donors. All procedures were approved by the Clinical Research Ethics Committee and the Ethics Review Board of The Third Affiliated Hospital of Nanjing Medical University (Approval No. [2024]KY029-01B). NHKs were cultured in keratinocyte-SFM (17005042, ThermoFisher, Waltham, MA, USA) according to the manufacturer’s instructions. To induce oxidative stress, HaCaT cells and NHKs were stimulated with H_2_O_2_ (21040210, Yonghua Chemical, Jiangsu, China). For functional studies, additional treatments included ethidium bromide (EtBr, HY-D0021, MedChemExpress, Shanghai, China), RU.521 (HY-114180, MedChemExpress), and Cyclosporin A (CsA, C804822, Macklin, Shanghai, China).

### Cell viability assay

Cells were seeded in 96-well plates at 5 × 10^3^ cells/well and allowed to adhere for 24 h. After treatment with H_2_O_2_ (24 h) or EtBr (48 h), 10 μL of MTT reagent (5 mg/mL, Beyotime, Shanghai, China, C0009S) was added to each well and incubated for 4 h at 37 °C with 5% CO_2_. Then, 100 μL of Formazan solubilization solution was added and incubated for 1–2 h until complete dissolution. Absorbance was measured at 570 nm using a microplate reader (TECAN, Männedorf, Switzerland). Cell viability was calculated as: (OD_treatment group_/OD_control group_) × 100%.

### Cell death assays

Keratinocytes were treated with 500 μM H_2_O_2_ for 24 h and then stained with Annexin V-FITC/PI (Beyotime, C1062S) for 15 min at room temperature. Cells were analyzed by flow cytometry (BD Accuri™ C6 Plus, New Jersey, USA), with Annexin V^+^/PI^−^ populations defined as early apoptotic cells, and Annexin V^+^/PI^+^ populations as late apoptotic cells. To assess mtDNA transfection-induced pyroptosis, two complementary approaches were employed. Cells were stained with Calcein-AM/PI (Beyotime, C2015S) for 30 min at 37 °C and imaged using a confocal laser scanning microscope (C2, Nikon, Tokyo, Japan), with PI-positive cells indicating pore formation. Concurrently, membrane integrity was quantified by measuring LDH release (Beyotime, C0019S) using a spectrophotometer at 450 nm.

### Detection of mitochondrial permeability transition pore (mPTP) opening

mPTP opening was assessed by a commercial Kit (Beyotime, C2009S). Cells were incubated with Calcein AM and CoCl_2_ at 37 °C for 30 min in the dark. After replacing with pre-warmed complete medium, cells were incubated for additional 30 min to ensure complete cytosolic Calcein AM cleavage. Mitochondrial Calcein retention (indicating closed mPTP) was imaged using a confocal laser scanning microscope (C2, Nikon), with CoCl_2_ quenching cytosolic/nuclear fluorescence.

### Detection of cytosolic mtDNA with immunofluorescent staining

Live keratinocytes were incubated with 400 nM MitoTracker™ Red CMXRos (ThermoFisher, M7512) for 15 min at 37 °C to label mitochondria. After fixation with 4% paraformaldehyde and permeabilization with 0.25% Triton X-100, cells were counterstained with 0.5 µg/mL PicoGreen (ThermoFisher, P7589) for double-stranded DNA (dsDNA) visualization. Fluorescent images were acquired using a confocal laser scanning microscope (C2, Nikon).

### Cellular fractionation and quantification of cytosolic mtDNA with qPCR

For comparative mtDNA quantification, approximately 6 × 10^6^ cells were equally divided for parallel DNA extraction. Total cellular DNA was isolated using the FastPure Cell/Tissue DNA Isolation Mini Kit (Vazyme, Nanjing, China, DC102-01), while cytosolic DNA was extracted by digitonin lysis buffer (50 mM HEPES pH 7.4, 150 mM NaCl, and 25 μg/mL digitonin). To determine mtDNA subcellular localization, purified mitochondria were treated with 20 mg/mL Proteinase K (Beyotime, ST533) to specifically degrade OMM proteins, enabling mtDNA analysis in the intermembrane space (IMS). Quantitative real-time PCR analysis was performed using mtDNA-specific primers, with data normalized to nuclear-encoded GAPDH from whole-cell lysates. Primer sequences are listed in Supplementary Table S1.

### mtDNA isolation and transfection

mtDNA was isolated from HaCaT cells and NHKs using a two-step purification procedure. First, mitochondria were isolated using a mitochondrial isolation kit (Beyotime, C3601) according to the manufacturer’s instructions. Subsequently, mtDNA was extracted from the isolated mitochondria using the FastPure Cell/Tissue DNA Isolation Mini Kit (Vazyme, DC102-01). For transfection, cells were seeded in six-well plates at a density of 5 × 10^5^ cells/well in antibiotic-free DMEM medium and cultured overnight. Transfection was carried out using Lipofectamine (Yeasen, Shanghai, China, 40802ES01) at a 1:1 ratio (μg DNA: μL reagent).

### Immunofluorescence analysis

Paraffin-embedded sections (human and mouse) were subjected to antigen retrieval in citrate buffer (95 °C, 15 min). Skin biopsies from vitiligo patients and healthy controls were incubated with anti-VDAC1 (Abcam, Cambridge, MA, UK, ab154856; 1:50). Mouse skin tissues were incubated with anti-TRP-1 (Abcam, ab178676; 1:100) to label melanocytes and anti-CD8 (Abcam, ab316778; 1:100) to detect cytotoxic T cells. Fixed HaCaT cells and NHKs were incubated with anti-CXCL9 (Abcam, ab290643; 1:50) and anti-CXCL10 (Abcam, ab318282; 1:50) overnight at 4 °C. All samples were then incubated with species-matched fluorophore-conjugated secondary antibodies (1:500) for 1 h at room temperature, counterstained with DAPI (1 μg/mL), and imaged using confocal laser scanning microscope (C2, Nikon). To minimize observer bias, histopathological and immunofluorescence assessments were performed independently by two observers who were blinded to the experimental groups.

### Masson-Fontana ammoniacal silver staining

To detect melanin pigment, formalin-fixed skin sections were stained as follows [27, 28]: slides were first washed three times with deionized water and then incubated in ammoniacal silver solution at room temperature for 12 h. After rinsed well in deionized water, slides were incubated in hypo solution for 5 min. Next, slides were rinsed again and counterstained with neutral red stain for another 5 min. Finally, following thorough rinsing, slides were observed under microscope.

### Transmission electron microscopy (TEM)

To examine mitochondrial ultrastructure, HaCaT cells were fixed overnight with 4% (w/v) glutaraldehyde in 0.1 M cacodylate buffer (pH 7.4) at 4 °C, followed by post-fixation with 2% (w/v) osmium tetroxide in the same buffer for 1 h at room temperature. After thorough rinsing with cacodylate buffer, samples were dehydrated through a graded ethanol series and embedded in epoxy resin. Ultrathin sections (70–90 nm) containing keratinocytes were cut using a diamond knife, stained with uranyl acetate and lead citrate, and observed under a transmission electron microscope (Hitachi HT7800, Tokyo, Japan).

### Quantitative real-time PCR analysis

Total RNA was extracted from cells using TRIzol reagent (Vazyme, R401-01) following the manufacturer’s instructions. RNA quality and concentration were determined by spectrophotometry (DeNovix, Delaware, USA). First-strand cDNA was synthesized from 1 μg of total RNA using HiScript IV RT SuperMix (Vazyme, R423-01). Quantitative real-time PCR was performed using ChamQ Blue Universal SYBR qPCR Master Mix Vazyme (Vazyme, Q312-02) on a real-time fluorescence quantitative PCR instrument (ThermoFisher, USA). The thermal cycling conditions consisted of an initial denaturation at 95 °C for 3 s followed by 40 cycles of 95 °C for 10 s and 60°C for 30 s. Relative gene expression was calculated by the 2^−^^ΔΔCt^ method with GAPDH as the endogenous control. Primer sequences are listed in Supplementary Table S1.

### Western blot analysis

Cell lysates were prepared using RIPA buffer (Beyotime, P0013) containing 1 mM PMSF (Beyotime, ST506), while skin tissue proteins were extracted using Histocyte Total Protein Extraction Kit (Applygen, Beijing, China, P1250) followed by centrifugation (12,000 rpm, 15 min, 4 °C). Protein concentrations were determined by BCA assay (Beyotime, P0011). Equal amounts of protein (40–60 μg) were separated by SDS-PAGE and transferred to PVDF/NC membranes via wet transfer system. After blocking with 5% (w/v) skimmed milk for 1.5 h at room temperature, membranes were incubated overnight at 4 °C with primary antibodies against target proteins (NLRP3, ab283819, 1:1000; Caspase-1, ab207802, 1:1000; human cGAS, sc-515777, 1:500; mouse cGAS, ab252416, 1:1000; human STING, ab239074, 1:1000; mouse STING, ab288157, 1:1000; p-NF-κB, ab76302, 1:1000; NF-κB, sc-8008, 1:200; GSDMD, HY-P83706, 1:1000; CXCL16, ab307694, 1:1000; human CXCL9, ab290643, 1:1000; mouse CXCL9, ab320827, 1:1000; human CXCL10, ab318282, 1:1000; mouse CXCL10, sc-374092, 1:200; IFN-γ, ab267369, 1:1000; VDAC1, ab186321, 1:1000; β-actin, AF0003, 1:1000). Next day, membranes were incubated with HRP-labeled Goat Anti-Rabbit IgG (A0208, 1:1000) and HRP-labeled Goat Anti-Mouse IgG (A0216, 1:1000) for 1 h at room temperature. Protein bands were visualized using ECL chemiluminescence. For data normalization, target proteins were normalized to β-actin, while phosphorylated proteins (p-NF-κB) were normalized to their total protein counterparts (NF-κB), oligomeric forms (VDAC1) to their monomeric states, and cleaved fragments (Caspase-1 p20, GSDMD-N) to their full-length proteins (pro-Caspase-1, GSDMD-FL).

### Cross-linking experiments

To assess VDAC1 oligomerization, cells were collected in PBS and incubated with 500 μM ethylene glycol bis (succinimidyl succinate) (EGS, Aladdin, E59534) for 20 min at 37 °C. The crosslinking reaction was terminated by adding 1.5 M Tris-HCl (pH 7.8) to a final concentration of 20 mM. Protein samples extracted from cell lysates were incubated at 37 °C for 10 min under non-denaturing conditions prior to Western blot analysis [29, 30].

### Enzyme-linked immunosorbent assay (ELISA)

The levels of CXCL9 and CXCL10 in human samples were quantified using the corresponding ELISA kits (CXCL9: Elabscience, Hubei, China, E-EL-H6062; CXCL10: Elabscience, E-EL-H0050), following the manufacturer’s protocols. Absorbance was recorded at 450 nm, and the concentrations of the samples were determined by comparison with standard curves generated from known concentrations.

### Small interfering RNA transfection

NHKs were transfected with VDAC1-targeting siRNA (Santa Cruz, Dallas, TX, USA, sc-42355) according to the manufacturer’s recommended protocol. Briefly, cells were transfected with siRNA complexed with siRNA Transfection Reagent (Santa Cruz, sc-29528) in antibiotic-free medium. Following 24 h incubation at 37 °C under standard culture conditions, transfected cells were subjected to subsequent experimental treatments as indicated.

### Statistical analysis

Data are presented as mean ± standard deviation (SD) from at least three independent experiments. The sample size was chosen based on established conventions in the field, and no statistical power calculation was used to predetermine the sample size a priori. The normality of data distribution was assessed for each group using the Shapiro-Wilk test. The homogeneity of variances was verified using the Brown-Forsythe test. Based on these assessments, all datasets were found to meet the assumptions for parametric tests. Statistical analyses were performed using GraphPad Prism version 10.4.0 (GraphPad Software). Comparisons between two groups were analyzed using a two-tailed unpaired Student’s *t* test. Comparisons among multiple groups were performed using one-way ANOVA followed by Tukey’s post hoc test. A *p*-value of less than 0.05 was considered statistically significant.

## Results

### Oxidative stress induces mtDNA release and activates the cGAS-STING pathway in keratinocytes

**Fig. 1:**
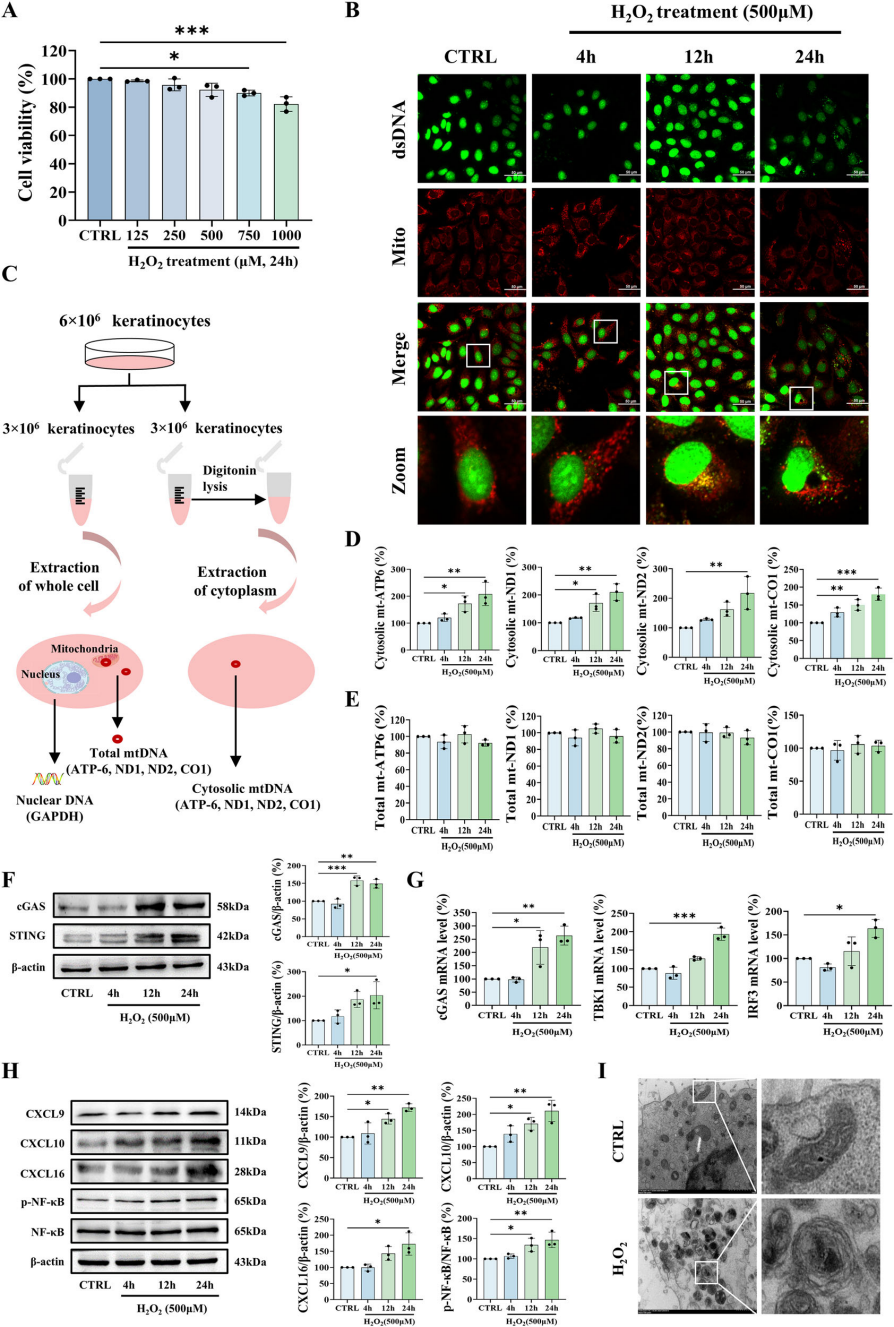
H_2_O_2_ treatment induces mtDNA release and activates the cGAS-STING pathway in HaCaT cells. **A** Cell viability measured by MTT assay after 24 h exposure to H_2_O_2_ (0–1000 μM). **B–H** Time-course analysis (4, 12, 24 h) of 500 μM H_2_O_2_-induced effects in HaCaT cells. **B** Double-immunofluorescence labeling of dsDNA (green) and mitochondria (red) in HaCaT cells. Scale bars, 50 μm. **C** Schematic workflow for cytosolic and total mtDNA extraction and quantification. Quantitative analysis of mtDNA (ATP6, ND1, ND2, CO1) in (**D**) cytosolic, and (**E**) whole-cell fractions by RT-qPCR. **F** Western blot analysis of cGAS and STING protein expression. **G** Transcript levels of cGAS, TBK1, and IRF3 measured by RT-qPCR. **H** Western blot analysis of CXCL9, CXCL10, CXCL16 and phosphorylated NF-κB (p-NF-κB). **I** Representative TEM images of mitochondria ultrastructure after 24 h H_2_O_2_ treatment. Data are presented as mean ± SD (*n* = 3). Asterisks ^*^ indicate a significant difference exists between indicated groups, ^*^P < 0.05, ^**^P < 0.01, ^***^P < 0.001. CTRL control, Mito mitochondria, TEM transmission electron microscopy.

In response to oxidative stress, mtDNA is released from mitochondria, triggering immune responses [14–16]. To uncover the role of mtDNA in keratinocytes under oxidative stress, we first assessed the levels of mtDNA released into the cytosol in HaCaT cells and NHKs. A concentration of 500 μM H_2_O_2_ was used according to the previous studies demonstrating its lack of cytotoxicity in keratinocytes [7, 8, 15]. Consistent with these findings, this treatment exhibited no significant cytotoxicity after 24 h (Figs. 1A and S1A), yet it effectively induced a marked increase in both intracellular ROS and mitochondrial superoxide (Fig. S2). Anti-dsDNA immunostaining showed an apparent increase in dsDNA signals outside mitochondria in both H_2_O_2_-treated HaCaT cells and NHKs compared to untreated controls (Figs. 1B and S1B). Subcellular fractionation was then used to quantify the increase in cytosolic mtDNA (Fig. 1C). RT-qPCR indicated that mtDNA levels in the cytosolic fraction were significantly increased, while total mtDNA remained stable (Figs. 1D, E and S1C, D).

Consistently, H_2_O_2_ activated the DNA-sensing cGAS-STING signaling pathway in HaCaT cells. Western blot analysis showed significant upregulation of cGAS and STING proteins (Fig. 1F), while qPCR revealed concomitant increases in mRNA levels of cGAS, TBK1 and IRF3 (Fig. 1G). This was accompanied by marked elevation of phosphorylated NF-κB (p-NF-κB) levels and chemokines (CXCL9, CXCL10, CXCL16) (Fig. 1H). Similar responses were observed in NHKs (Fig. S1E). Furthermore, transmission electron microscopy revealed pronounced cristae disorganization and mitochondrial vacuolization in H_2_O_2_-treated HaCaT cells, a phenomenon also linked to the release of mtDNA (Fig. 1I). Collectively, these results demonstrate that H_2_O_2_-induced oxidative stress causes mitochondrial damage and subsequent mtDNA release in both HaCaT cells and NHKs.

### Reduced cytosolic mtDNA attenuates oxidative stress-induced immune responses in keratinocytes

**Fig. 2:**
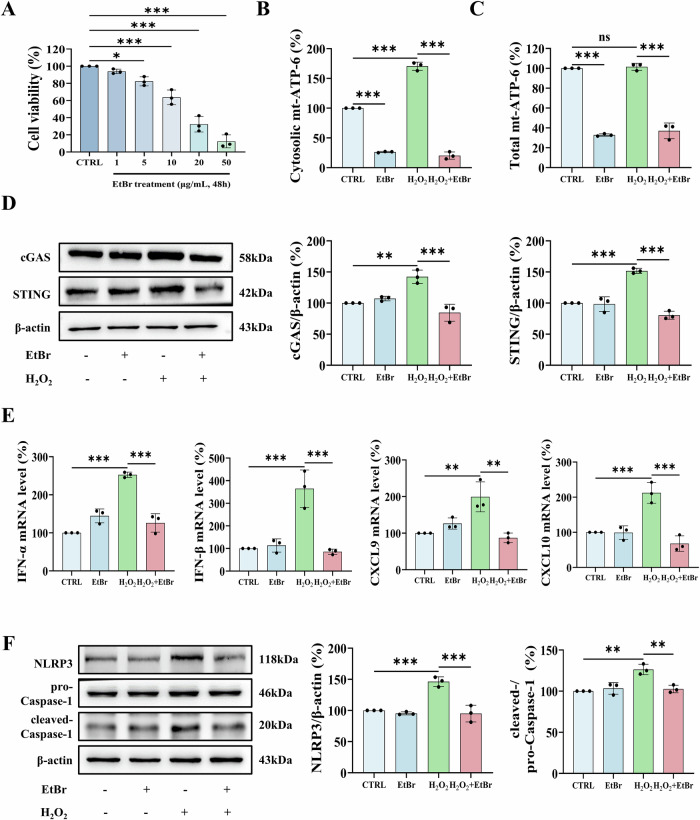
Cytosolic mtDNA reduction attenuates H_2_O_2_-induced immune responses in HaCaT cells. **A** Cytotoxicity assessment of EtBr (0–50 μg/mL, 48 h) by MTT assay. **B-F** HaCaT cells were pre-treated with 0.2 μg/mL EtBr (48 h) followed by 500 μM H_2_O_2_ (24 h). Quantitative analysis of mtDNA (ATP6) in (**B**) cytosolic, and (**C**) whole-cell fractions by RT-qPCR. **D** Western blot analysis of cGAS and STING protein expression. **E** Transcript levels of IFN-α, IFN-β, CXCL9 and CXCL10 measured by RT-qPCR. **F** Western blot analysis of NLRP3 and cleaved Caspase-1. Data are presented as mean ± SD (*n* = 3). Asterisks ^*^ indicate a significant difference exists between indicated groups, ^*^P < 0.05, ^**^P < 0.01, ^***^P < 0.001. CTRL control, EtBr ethidium bromide.

To investigate the role of cytosolic mtDNA in H_2_O_2_-treated keratinocytes, we depleted mtDNA using ethidium bromide (EtBr), which selectively inhibits mtDNA replication at low concentrations (0.1–2 μg/mL) without affecting nuclear DNA [31]. Cell viability was tested and the final concentration of 0.2 μg/mL EtBr for 48 h was used to minimize cytotoxicity (Figs. 2A and S3A). RT-qPCR analysis confirmed that EtBr treatment effectively reduced both cytosolic and total mtDNA levels in HaCaT cells and NHKs (Figs. 2B, C and S3B, S3C). Consistent with our hypothesis, mtDNA depletion abolished H_2_O_2_-induced activation of the cGAS-STING pathway (Figs. 2D and S3D), and significantly suppressed upregulation of type I interferons (IFN-α/β) and chemokines (CXCL9, CXCL10) (Figs. 2E and S3E). Furthermore, EtBr treatment inhibited H_2_O_2_-triggered NLRP3 inflammasome activation, as evidenced by reduced NLRP3 expression and caspase-1 cleavage (Figs. 2F and S3D). Collectively, these findings indicate that mtDNA is essential for initiating oxidative stress-induced immune responses in keratinocytes, and its depletion effectively attenuates inflammatory signaling.

### Cytosolic mtDNA directly activates immune responses in keratinocytes via the cGAS-STING pathway

**Fig. 3:**
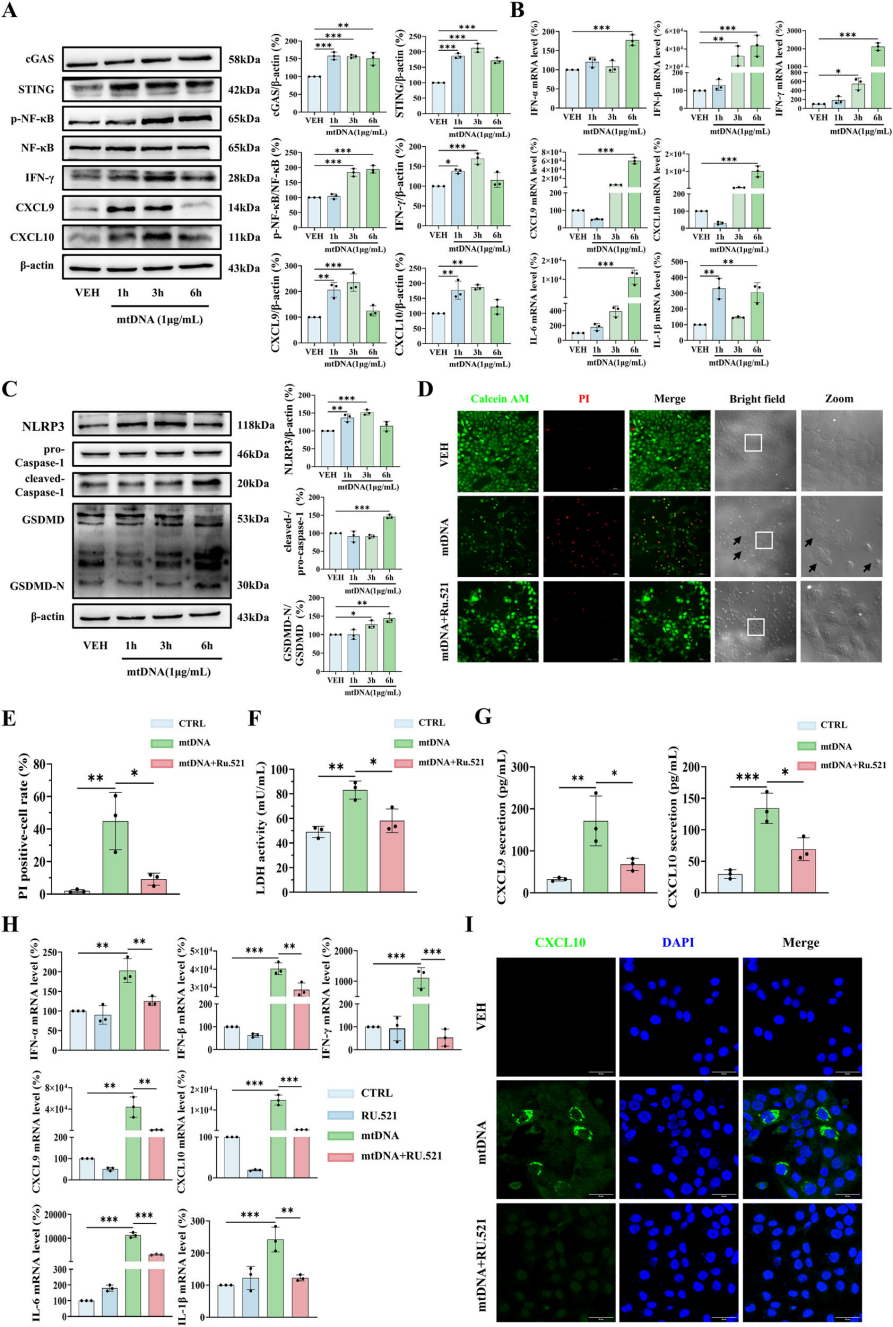
Cytosolic mtDNA activates immune responses via cGAS-STING-NLRP3 axis in HaCaT cells. **A–C** Time-course analysis (1, 3, 6 h) of 1 μg/mL mtDNA transfection-induced effects in HaCaT cells. **A** Western blot analysis of cGAS, STING, p-NF-κB, IFN-γ, CXCL9, and CXCL10. **B** Transcript levels of IFN-α, IFN-β, IFN-γ, CXCL9, CXCL10, IL-6, and IL-1β by RT-qPCR. **C** Western blot analysis of NLRP3, cleaved Caspase-1 and GSDMD-N protein expression. **D–I** HaCaT cells transfected with 1 μg/mL mtDNA ±10 μM RU.521 (cGAS inhibitor, 1 h pretreatment). **D** Representative image of PI/Calcein AM staining. Scale bars, 100 μm. **E** Quantification of PI-positive cells in three randomly chosen fields (one field per well). **F** LDH release in culture supernatants. **G** Secretion level of CXCL9 and CXCL10 in culture supernatants by ELISA. **H** Transcript levels of IFN-α, IFN-β, IFN-γ, CXCL9, CXCL10, IL-6, and IL-1β by RT-qPCR. **I** CXCL10 expression by immunofluorescence. Scale bars, 50 μm. Data are presented as mean ± SD (*n* = 3). Asterisks ^*^ indicate a significant difference exists between indicated groups, ^*^P < 0.05, ^**^P < 0.01, ^***^P < 0.001. VEH vehicle, GSDMD-N gasdermin D N-terminal fragment, PI propidium iodide, LDH lactate dehydrogenase.

To confirm the immunostimulatory effects of cytosolic mtDNA, we isolated and purified mtDNA from HaCaT cells (with <0.01% nuclear DNA contamination validated by RT-qPCR; Fig. S4A), and transfected it back into HaCaT cells. The transfected mtDNA activated both cGAS-STING and NF-κB pathways, leading to significantly increased protein expression of IFN-γ, CXCL9, and CXCL10 (Fig. 3A). Transcriptional analysis showed that mtDNA induced robust upregulation of IFN-β, IFN-γ, CXCL9, CXCL10, and IL-6, along with moderate increases in IFN-α and IL-1β (Fig. 3B). This immune response was dose-dependent, as indicated by escalating IFN-α, IFN-β, and CXCL9 mRNA levels (Fig. S4B).

Notably, mtDNA transfection triggered canonical NLRP3 inflammasome activation, caspase-1 and GSDMD cleavage in HaCaT cells (Fig. 3C), which mediates pyroptosis, a well-characterized inflammatory cell death mechanism [32]. Accordingly, following mtDNA exposure over a time course (1, 3, and 6 h), we observed hallmark features of pyroptosis including: (1) substantial depletion of intracellular IFN-γ, CXCL9 and CXCL10 (Fig. 3A); (2) increased propidium iodide (PI)-positive staining cells displaying characteristic pyroptotic morphology including membrane blebbing and cellular shrinkage (Figs. 3D, E); and (3) elevated LDH release alongside elevation of extracellular CXCL9 and CXCL10 levels (Fig. 3F, G), confirming pyroptosis-mediated protein efflux.

Furthermore, the central role of cGAS-STING signaling was confirmed through pharmacological inhibition, where pretreatment with the cGAS inhibitor RU.521 prevented cell pyroptosis and significantly attenuated the production of inflammatory cytokines and chemokines (Fig. 3D–I). Consistent with these findings, mtDNA transfection in NHKs also induced pyroptosis and LDH release after 24 h, which were effectively suppressed by RU.521 (Fig. S5A–C). Together, these findings demonstrate that cytosolic mtDNA acts as a potent endogenous immunostimulatory molecule in keratinocytes, triggering inflammatory responses and pyroptotic cell death through activation of the cGAS-STING-NLRP3 axis.

### Pharmacological inhibition of cGAS-STING pathway alleviates oxidative stress-induced immune responses in keratinocytes

**Fig. 4:**
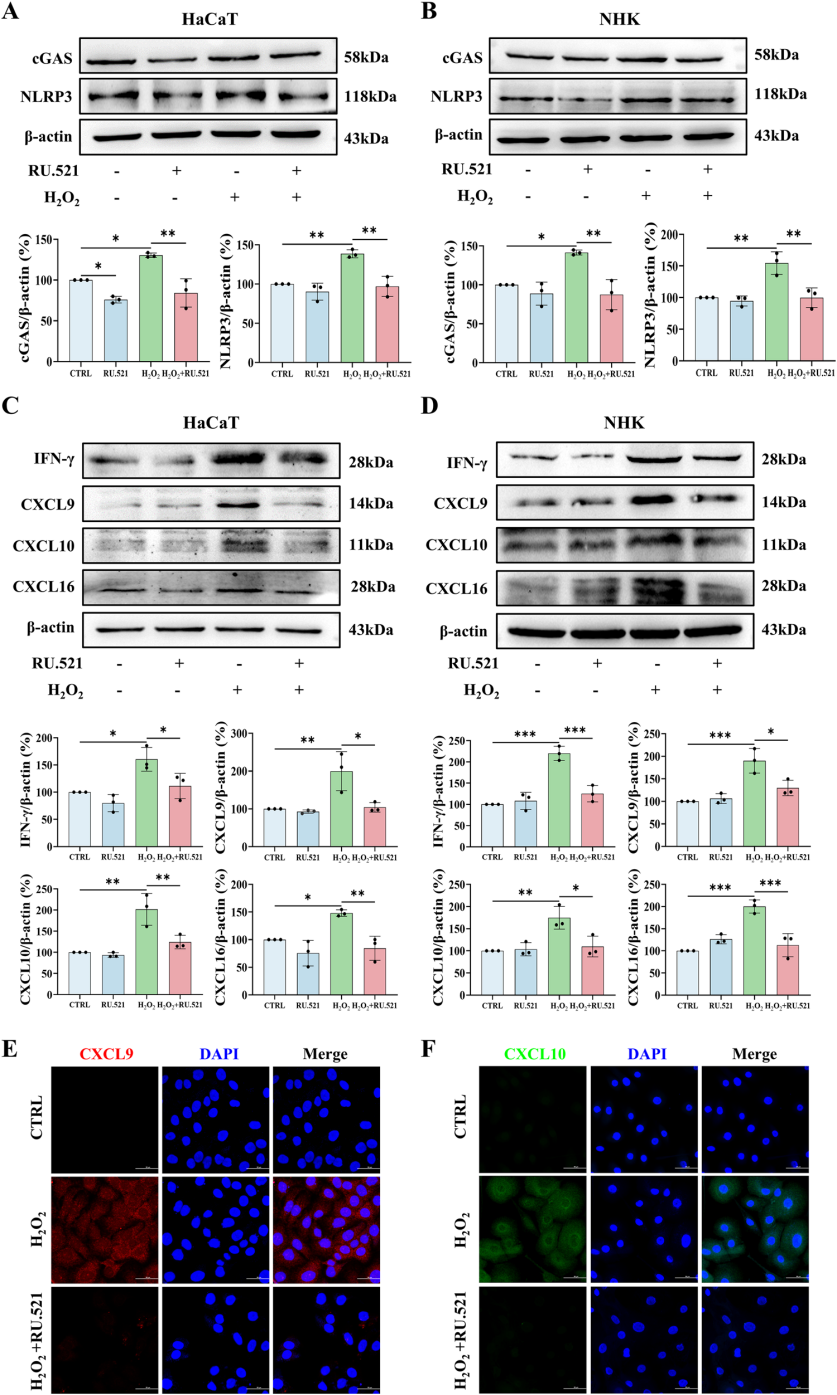
RU.521 pretreatment alleviates H_2_O_2_-induced immune responses in HaCaT cells and NHKs. Cells were pretreated with 10 μM RU.521 (1 h) prior to H_2_O_2_ stimulation. Western blot analysis of cGAS and NLRP3 in (**A**) HaCaT cells, and (**B**) NHKs. Western blot analysis of IFN-γ, CXCL9, CXCL10 and CXCL16 in (**C**) HaCaT cells, and (**D**) NHKs. **E** CXCL9, and **F** CXCL10 expression in NHKs by immunofluorescence. Scale bars, 50 μm. Data are presented as mean ± SD (*n* = 3). Asterisks ^*^ indicate a significant difference exists between indicated groups, ^*^P < 0.05, ^**^P < 0.01, ^***^P < 0.001.

To elucidate the downstream mechanism underlying mtDNA release-triggered immune responses, we focused on the cGAS-STING axis, a well-established sensor system for cytosolic DNA [25]. In keeping with this, H_2_O_2_ treatment robustly activated the cGAS-STING pathway in both HaCaT cells and NHKs (Figs. 1F, G and S1E). Notably, pharmacological inhibition of cGAS with RU.521 effectively suppressed H_2_O_2_-induced activation of both cGAS signaling and the NLRP3 inflammasome (Fig. 4A, B), and further attenuated upregulation of key inflammatory mediators, including IFN-γ and chemokines (CXCL9, CXCL10, and CXCL16) in HaCaT cells and NHKs (Fig. 4C–F). In summary, these findings demonstrate that the cGAS-STING-NLRP3 axis, triggered by cytosolic release of mtDNA, contributed to the immune responses in H_2_O_2_ -treated keratinocytes.

### VDAC1 oligomerization and mPTP opening mediate oxidative stress-induced mtDNA release in keratinocytes

**Fig. 5:**
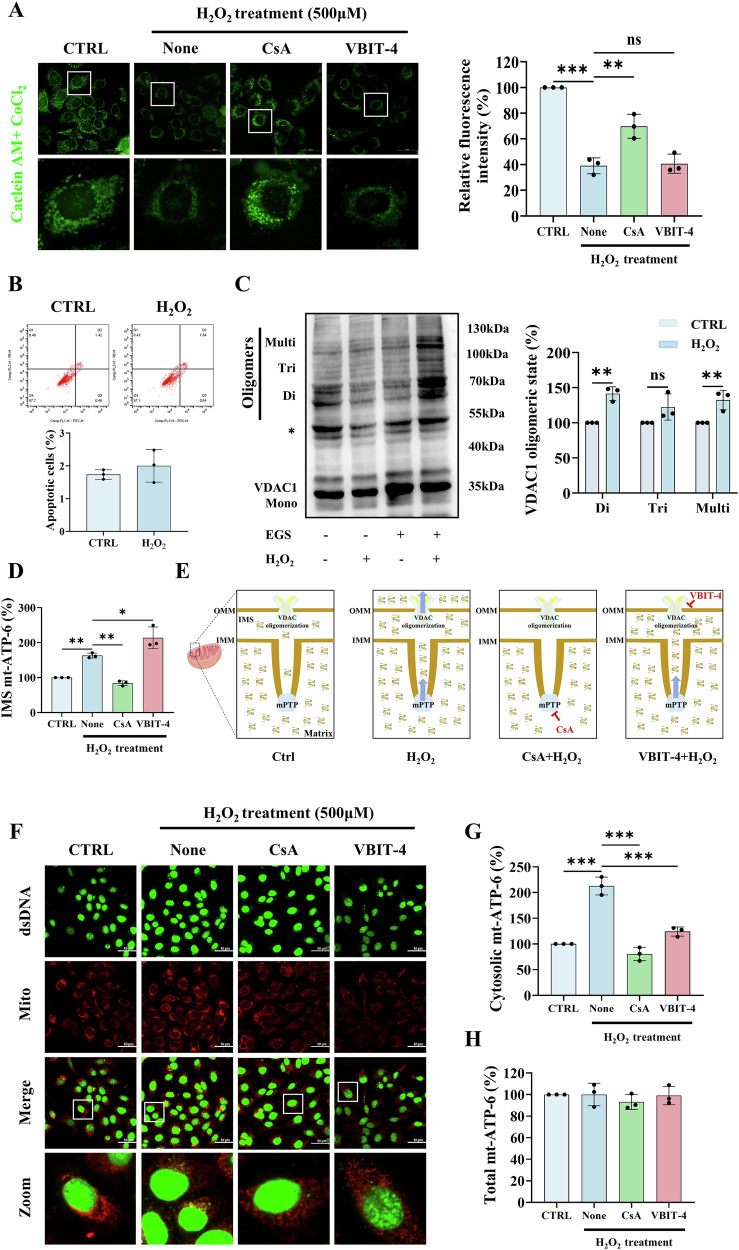
VDAC1 oligomerization and mPTP opening mediate H_2_O_2_-induced mtDNA release in HaCaT cells. **A** Immunofluorescence of Calcein AM staining in (i) CTRL, (ii) H_2_O_2_-only, (iii) H_2_O_2_ + CsA, and (iv) H_2_O_2_ + VBIT-4 groups. Scale bars, 50 μm. Fluorescence intensity was normalized relative to the CTRL group. **B** Flow cytometry analysis of cell apoptosis after H_2_O_2_ stimulation (500 μM, 24 h). **C** Western blot analysis of VDAC1 oligomerization after H_2_O_2_ treatment (500 μM, 24 h). Asterisk indicates a nonspecific band. **D** Quantification of mtDNA level (ATP6) in the IMS by RT-qPCR. **E** Four-panel schematic of mtDNA translocation. Panel1: untreated mitochondria; Panel2: H_2_O_2_-induced mPTP/VDAC1 activation; Panel3: CsA blocks IMM permeabilization; Panel4: VBIT-4 inhibits OMM export. **F** Immunofluorescence of dsDNA (green) and mitochondria (red) in (i) CTRL, (ii) H_2_O_2_-only, (iii) H_2_O_2_ + CsA, and (iv) H_2_O_2_ + VBIT-4 groups. Scale bars, 50 μm. Quantitative analysis of mtDNA (ATP6) in (**G**) cytosolic, and (**H**) whole-cell fractions by RT-qPCR. Data are presented as mean ± SD (*n* = 3). Asterisks ^*^ indicate a significant difference exists between indicated groups, ^*^P < 0.05, ^**^P < 0.01, ^***^P < 0.001. CsA cyclosporin A, mPTP mitochondrial permeability transition pore, IMS mitochondrial intermembrane space, IMM inner mitochondrial membrane, OMM outer mitochondrial membrane.

Having established the immunogenic role of cytosolic mtDNA in keratinocytes, we next investigated the mechanisms underlying mtDNA translocation across mitochondrial membranes. H_2_O_2_, a well-established inducer of both mPTP opening and VDAC1 oligomerization [21, 22], elicited distinct mitochondrial membrane permeabilization events in keratinocytes. We first observed excessive mPTP opening evidenced by calcein fluorescence quenching (Figs. 5A and S6A), with concurrent absence of apoptotic markers (Figs. 5B and S6B), confirming the apoptosis-independent nature of these events. Importantly, VDAC1 oligomerization was significantly induced (Figs. 5C and S6C), demonstrating that H_2_O_2_ treatment induces both IMM (via mPTP) and OMM (via VDAC1 oligomers) permeabilization under non-apoptotic conditions.

To delineate the mechanistic relationship between these events, we employed pharmacological inhibitors targeting distinct mitochondrial components: cyclosporin A (CsA) for mPTP inhibition and VBIT-4 for VDAC1 oligomerization blockade. Pretreatment with CsA, but not VBIT-4, prevented the H_2_O_2_-induced decrease in calcein fluorescence (Figs. 5A and S6A), establishing mPTP opening as the upstream event. This temporal hierarchy was further substantiated by mitochondrial intermembrane space (IMS) mtDNA quantification after proteinase K treatment (20 μg/mL) [20], which showed opposing effects: CsA decreased IMS mtDNA (indicating impaired matrix-to-IMS translocation), whereas VBIT-4 increased IMS accumulation (demonstrating OMM export blockade) (Figs. 5D and S6D). This opposing action of CsA and VBIT-4 was captured in a schematic model (Fig. 5E).

Correspondingly, immunofluorescence analysis confirmed reduced cytosolic dsDNA signals with CsA or VBIT-4 pretreatment (Fig. 5F). Quantification assays revealed that while total cellular mtDNA levels remained unchanged (Figs. 5H and S6F), CsA and VBIT-4 reduced H_2_O_2_-induced cytosolic mtDNA release by 50–60% and 40%, respectively (Figs. 5G and S6E). Together, these in vitro results demonstrate a mechanism by which H_2_O_2_ induces mPTP opening, ultimately resulting in VDAC1-dependent mtDNA release into the cytoplasm.

### VDAC1 inhibition ameliorates the immune responses in vitro and in vivo

**Fig. 6:**
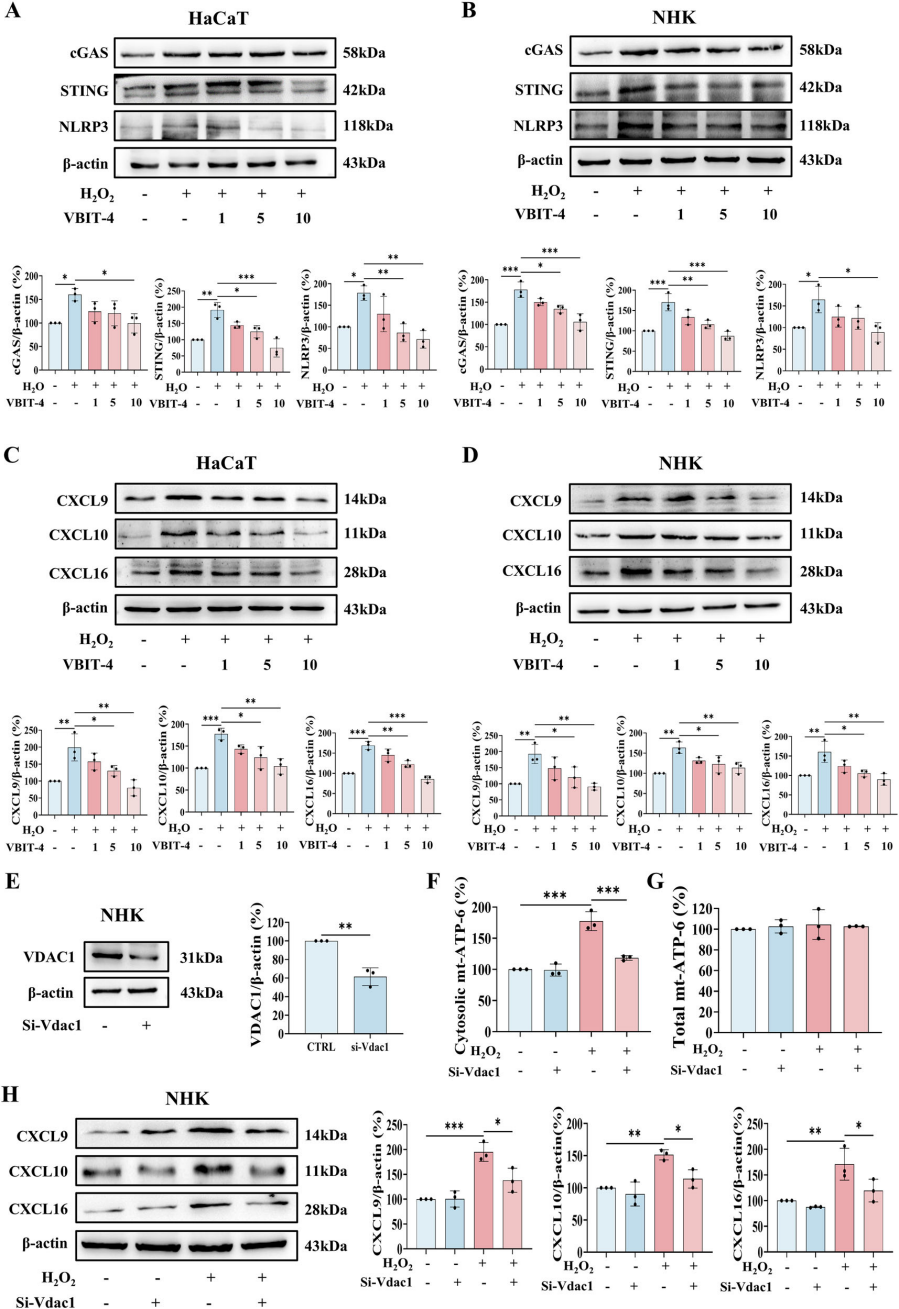
Inhibition of VDAC1 attenuates H_2_O_2_-induced immune responses in keratinocytes. **A**–**D** Cells were pretreated with VBIT-4 (1, 5, or 10 μM, 1 h) prior to H_2_O_2_ stimulation. Western blot analysis of cGAS, STING, and NLRP3 in (**A**) HaCaT cells, and (**B**) NHKs. Western blot analysis of CXCL9, CXCL10, and CXCL16 in (**C**) HaCaT cells, and (**D**) NHKs. **E**–**H** Vdac1 were genetically silenced by siRNA in NHKs. **E** Western blot validation of Vdac1 knockdown efficiency in NHKs. Quantitative analysis of mtDNA (ATP6) in (**F**) cytosolic, and (**G**) whole-cell fractions by RT-qPCR. **H** Western blot analysis of CXCL9, CXCL10, and CXCL16 in NHKs. Data are presented as mean ± SD (*n* = 3). Asterisks ^*^ indicate a significant difference exists between indicated groups, ^*^P < 0.05, ^**^P < 0.01, ^***^P < 0.001.

We next examined whether pharmacological inhibition of VDAC1 oligomerization could ameliorate H_2_O_2_-induced immune responses in keratinocytes. In both HaCaT cells and NHKs, treatment with VBIT-4 remarkably inhibited the cGAS-STING pathway and NLRP3 inflammasome induced by H_2_O_2_ (Fig. 6A, B). Consistent with these findings, VBIT-4 treatment also markedly reduced the expression of downstream chemokines (CXCL9, CXCL10, and CXCL16) (Fig. 6C, D).

To further validate the role of VDAC1, we genetically silenced *Vdac1* in NHKs using siRNA, achieving approximately 50% knockdown efficiency (Fig. 6E). Notably, *Vdac1* deficiency significantly attenuated H_2_O_2_-induced cytosolic mtDNA accumulation (Fig. 6F, G), accompanied by a reduction in the expression of CXCL9, CXCL10, and CXCL16 (Fig. 6H). These results collectively demonstrate that VDAC1 oligomerization inhibition, either pharmacologically or genetically, effectively dampens H_2_O_2_-triggered innate immune responses in keratinocytes by restraining mtDNA release and subsequent cGAS-STING/NLRP3 inflammasome activation.

**Fig. 7:**
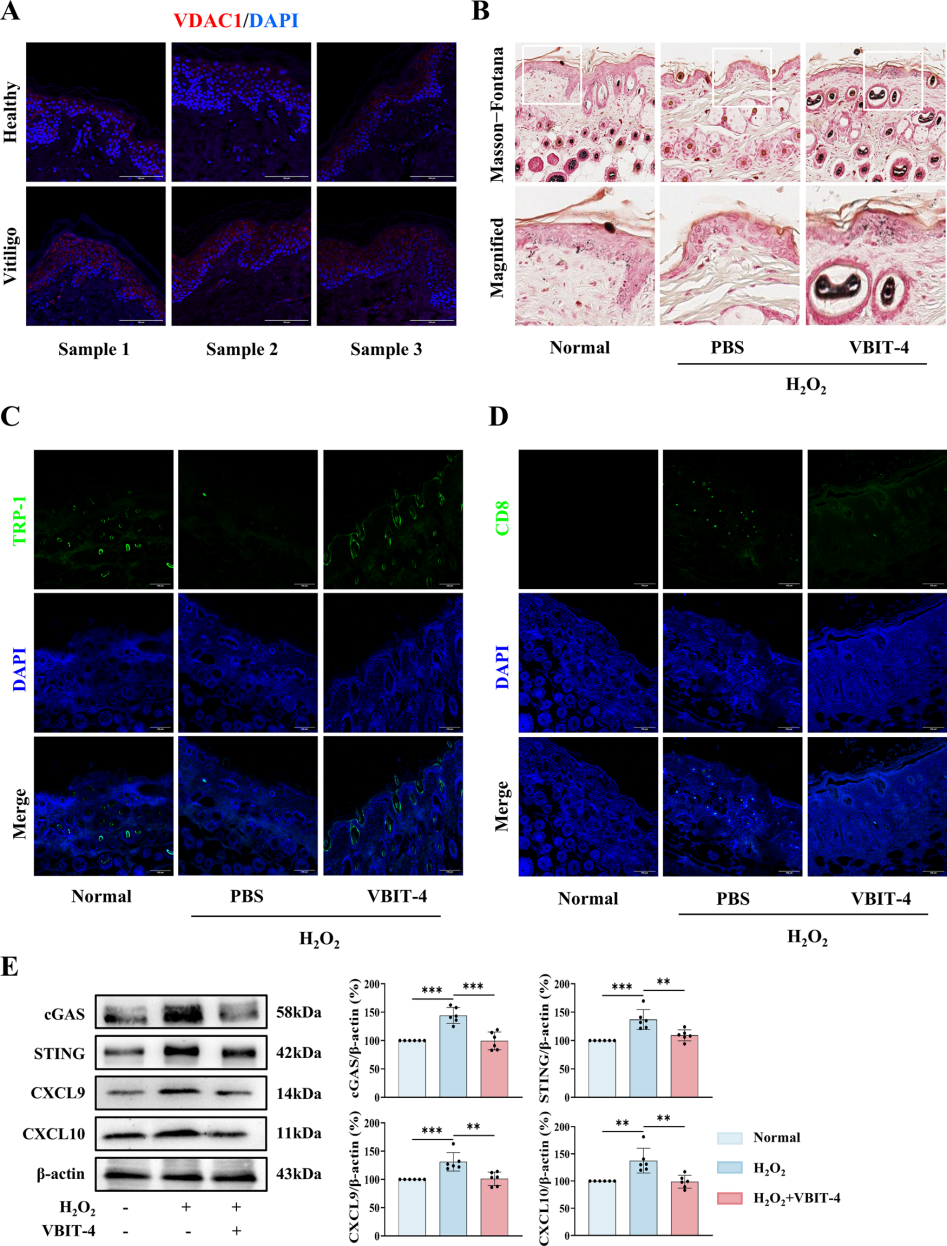
VBIT-4 alleviates vitiligo progression by targeting VDAC1-mediated melanocyte loss and immune activation. **A** VDAC1 expression in perilesional skin of vitiligo patients versus healthy controls, assessed by IHC. Scale bars, 100 μm. **B**–**E** H_2_O_2_-induced vitiligo mice were used to determine the effects of VBIT-4. **B** Representative images of Masson-Fontana ammoniacal silver staining of skin biopsies. **C** Immunofluorescence staining of TRP-1^+^ melanocytes. Scale bars, 100 μm. **D** Immunofluorescence staining of CD8^+^ T cells. Scale bars, 100 μm. **E** Western blot analysis of cGAS, STING, CXCL9, CXCL10 in lesional skin. Data are presented as mean ± SD (*n* = 6). Asterisks ^*^ indicate a significant difference exists between indicated groups, ^**^P < 0.01, ^***^P < 0.001^.^ IHC immunohistochemistry.

Building upon our in vitro findings that VDAC1 oligomerization mediates mtDNA-induced immune responses, we first confirmed the clinical relevance of VDAC1 upregulation by demonstrating its significant increase in vitiligo patient perilesions versus healthy controls (Fig. 7A). In H_2_O_2_-induced vitiligo mice, VBIT-4 treatment markedly attenuated depigmentation, as evidenced by Masson-Fontana ammoniacal silver staining (Fig. 7B). Importantly, immunofluorescence analysis revealed that VBIT-4 restored the density of TRP-1^+^ melanocytes in lesion skin, confirming its protective effect on melanocyte survival (Fig. 7C). Furthermore, VBIT-4 significantly suppressed CD8⁺ T cell infiltration, as shown by diminished CD8^+^ staining in treated mice (Fig. 7D). Mechanistically, VBIT-4 inhibited the activation of the cGAS-STING pathway, leading to reduced production of downstream chemokines CXCL9 and CXCL10 in perilesional skin (Fig. 7E). In conclusion, intradermal administration of VBIT-4 effectively alleviated skin depigmentation and reduced inflammation in the H_2_O_2_-induced vitiligo mouse model, suggesting its therapeutic potential for vitiligo treatment.

## Discussion

In vitiligo, while melanocytes are the primary targets of immune-mediated destruction, keratinocytes are equally pivotal in shaping the pathological immune microenvironment of the disease [4–6]. Recently, mtDNA has emerged as a key player in vitiligo pathogenesis. Sant’Anna et al. [14] showed that aberrant mtDNA release from vitiligo melanocytes activates the cGAS-STING pathway, inducing pro-inflammatory cytokines and chemokines for CD8^+^ T cell recruitment, while Xu et al. [15] found that such release also triggers melanocyte pyroptosis, further promoting CD8^+^ T cell activation. Furthermore, Wu et al. [16] found that elevated mtDNA from keratinocytes exacerbates vitiligo progression by activating the cGAS-STING-IFN-α/β axis in monocytes, amplifying IFN-γ^+^CD8^+^ T cells functions while decreasing regulatory Treg.

**Fig. 8:**
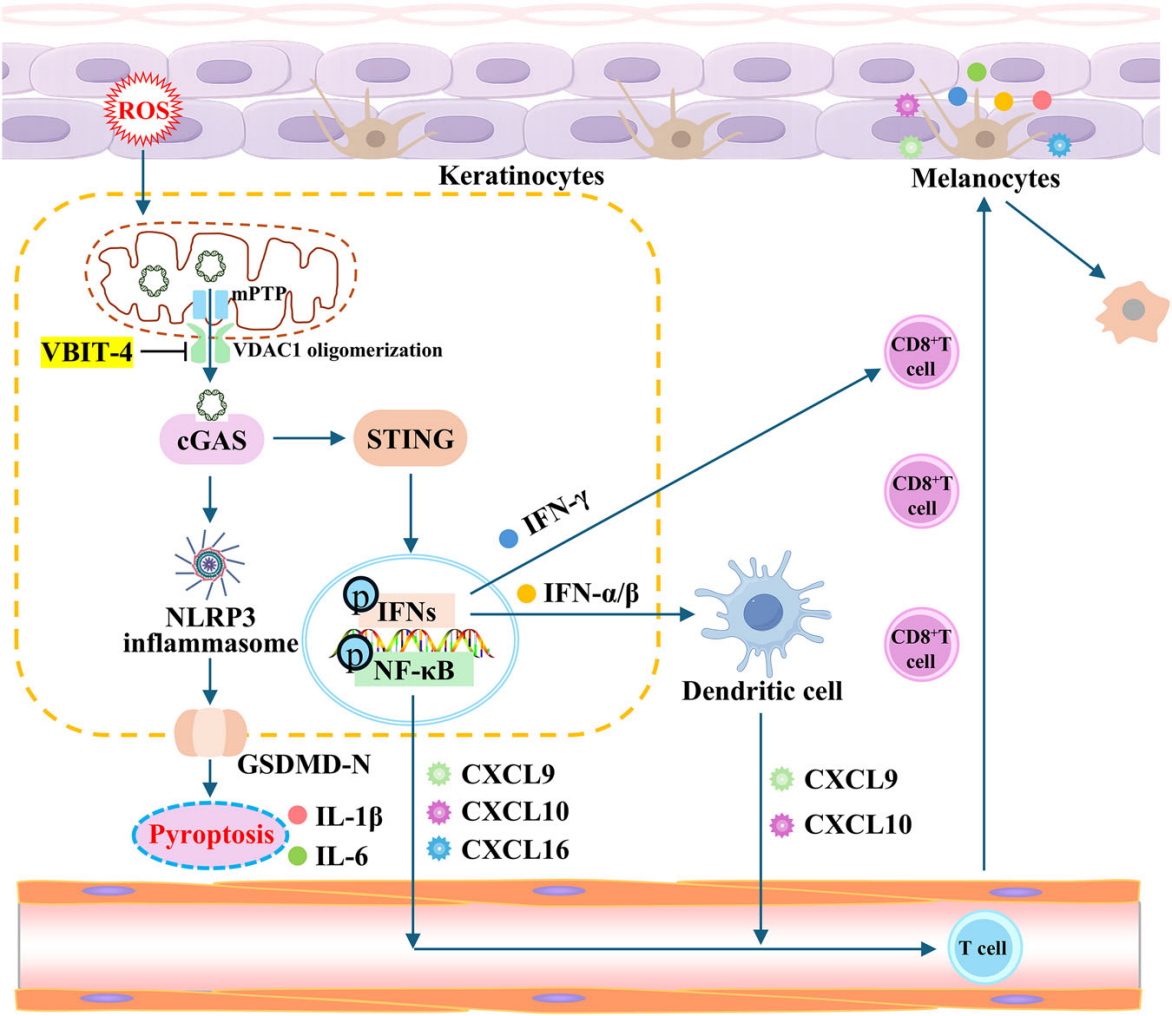
VDAC1-mediated mtDNA release activates keratinocyte innate immunity in vitiligo. Under oxidative stress, keratinocytes release mitochondrial DNA through sequential mPTP opening and VDAC1 oligomerization. The escaped cytosolic mtDNA then acts as a danger signal that concurrently activates both the cGAS-STING axis and the NLRP3 inflammasome, driving the production of type I (IFN-α/β) and type II (IFN-γ) interferons, the release of chemokines, and pyroptosis. These mediators shape a pro-inflammatory microenvironment that promotes the recruitment and retention of CD8⁺ T cells and amplifies local immune activation in vitiligo. Pharmacological inhibition of VDAC1 oligomerization by VBIT-4 disrupts this upstream event, suggesting a potential therapeutic strategy to limit disease progression.

Consistent with previous reports [15, 16], our study confirmed oxidative stress-induced mitochondrial damage and mtDNA release in keratinocytes (Figs. 1 and S1). However, the novelty of our research lies in further elucidating both the mechanism of oxidative stress-induced mtDNA release and its downstream immunological consequences in keratinocytes (Fig. 8). We identified a sequential mitochondrial membrane permeabilization process in which H_2_O_2_ initially induces mPTP-dependent IMM opening followed by VDAC1 oligomerization at the OMM, a distinct pathway from classical apoptotic mtDNA release that enables selective mtDNA escape during sub-lethal stress. Importantly, we demonstrated that released mtDNA coordinately activates both the cGAS-STING axis and NLRP3 inflammasome in keratinocytes, establishing an auto-amplifying inflammatory circuit. This dual signaling equips keratinocytes with a powerful, two-pronged immune-activating program: cGAS-STING drives T-cell recruitment [14, 16], while NLRP3-mediated pyroptosis licenses inflammation and disrupts barrier integrity [32–34]. Together, they create a self-perpetuating inflammatory loop that transforms keratinocytes into active instigators of vitiligo pathogenesis.

Although the mtDNA-cGAS-STING pathway is classically associated with type I interferon (IFN-α/β) and NF-κB activation [25], our findings reveal its broader role in vitiligo through the dual induction of both type I and type II interferon pathways in keratinocytes. While IFN‑γ is the established central effector cytokine that drives CD8⁺ T‑cell‑mediated melanocyte destruction [35], our data demonstrate that aberrant mtDNA simultaneously upregulates both IFN-α/β and IFN-γ expression in keratinocytes (Fig. 3). Notably, the more pronounced inhibition of IFN-γ compared to IFN-α/β by the cGAS inhibitor RU.521 suggests either a greater dependence of IFN-γ on cGAS-STING signaling or the existence of an amplification mechanism specific to this pathway. Building on evidence that IFN‑α/β can initiate early immune priming [36, 37], our results support a two-phase model mediated by keratinocytes via the mtDNA–cGAS–STING axis. In the initiation phase, keratinocyte-derived IFN‑α/β activates dendritic cells and induces early CXCL9/10 production to recruit T cells. In the amplification phase, sustained cGAS–STING-driven IFN‑γ promotes cytotoxic T‑cell recruitment and melanocyte destruction (Fig. 8). Thus, the keratinocyte-intrinsic mtDNA–cGAS–STING axis coordinates both the initiation and amplification of vitiligo pathogenesis through temporally distinct interferon programs. The precise temporal dynamics and cross-talk between these pathways warrant further study.

Due to the similarities in the cGAS-STING signaling axis and NLRP3 inflammasome in response to cellular stress as well as the downstream effects, recent studies have focused on their relationship. Indeed, the cGAS-STING-NLRP3 axis has emerged as a well-characterized pathway mediating inflammation and pyroptosis [38, 39]. Mechanistically, cytosolic mtDNA itself represents a key upstream ligand that can converge on both pathways [24]. In the present study, depletion of mtDNA using EtBr attenuated activation of both cGAS-STING and NLRP3 (Fig. 2), indicating that mtDNA functions as a shared trigger. In addition, inhibition of the mtDNA sensor cGAS with RU.521 partially suppressed NLRP3 inflammasome activation (Fig. 4), suggesting that the two pathways are not strictly parallel, and that cGAS-STING signaling contributes to NLRP3 activation. To further delineate the specificity of the mtDNA signal, we compared its effects with generic oxidative stress. Notably, exogenous mtDNA transfection not only amplified the inflammatory responses but also triggered significant pyroptotic cell death (Figs. 3 and S5). In contrast, H_2_O_2_ treatment induced robust cytokine production without causing cell death (Figs. 5 and S6), consistent with prior reports [8]. While Li et al. [8] proposed that “NLRP3 activation and pyroptosis may be uncoupled,” our results suggest an alternative interpretation: H_2_O_2_ may be insufficient to fully engage pyroptotic machinery, whereas mtDNA provides a stronger and more direct activation signal. Collectively, these findings highlight that mtDNA-driven inflammation and pyroptosis as a novel mechanism contributing to the keratinocyte damage. Further studies should explore whether threshold-dependent activation or temporal dynamics of inflammasome signaling account for the observed differences between H_2_O_2_ and mtDNA.

Accumulating evidence has established mtDNA as a key DAMP driving immune activation in vitiligo [14–16], yet the mechanism for this release remains poorly understood. Here, our study elucidates a sequential mechanism by which oxidative stress triggers mtDNA release in keratinocytes: mPTP opening at the IMM precedes VDAC1 oligomerization at the OMM, ultimately leading to cytosolic mtDNA release (Figs. 5 and S6). This finding aligns with emerging evidence that mtDNA release is tightly regulated by mitochondrial membrane dynamics, where VDAC1 serves as a critical gatekeeper under non-apoptotic stress conditions [23, 40, 41]. Importantly, this mechanism differs fundamentally from apoptosis-associated mtDNA release, which is initiated by BAX/BAK macropore formation that allows the simultaneous release of mtDNA together with apoptotic factors [17, 18]. As a result, apoptotic mtDNA release is largely immunologically silent because caspase activation suppresses mtDNA-triggered cGAS-STING signaling [17]. In contrast, the VDAC1-oligomerization-dependent pathway enables keratinocytes to release immunogenic mtDNA as a potent danger signal, thereby sustaining the chronic immune activation characteristic of vitiligo.

Most importantly, the therapeutic potential of targeting VDAC1 is highlighted by its ability to suppress mtDNA-driven immune activation. Pharmacological (VBIT-4) or genetic deletion of VDAC1 not only blocks mtDNA release but also attenuates downstream cGAS-STING and NLRP3 inflammasome activation in vitro, thereby disrupting the inflammatory cascade central to vitiligo pathogenesis (Fig. 6). Furthermore, the efficacy of VDAC1 inhibition was confirmed in vivo (Fig. 7), suggesting a promising translational avenue. Unlike broad immunosuppressants (e.g., CsA), therapeutic targeting of VDAC1 precisely intercepts the disease process at its origin by blocking mtDNA release while preserving physiological immune functions. Our findings align with recent advances in mitochondrial medicine, where VDAC1 inhibition has shown promise in several inflammation-related diseases [41, 42], and suggest its potential repurposing for vitiligo treatment, particularly in early disease stages where mitochondrial dysfunction precedes established autoimmunity.

## Conclusion

In conclusion, our study elucidates a novel VDAC1-dependent mechanism by which oxidative stress triggers mtDNA release from keratinocytes, subsequently activating both cGAS-STING and NLRP3 inflammasome pathways to drive vitiligo-associated inflammation. We demonstrate that this mtDNA release initiates a dual interferon (IFN-α/β and IFN-γ) response and promotes keratinocyte pyroptosis, thereby shaping the autoimmune microenvironment. Our findings establish VDAC1-mediated mtDNA release as a critical pathogenic event and highlight the therapeutic promise of targeting mitochondrial membrane dynamics. However, key questions remain regarding extracellular mtDNA’s role in melanocyte damage and the precise triggers of VDAC1 oligomerization. These findings highlight the potential of mitochondrial membrane regulation as a promising intervention point for vitiligo, while underscoring the need to explore mitochondrial-nuclear crosstalk in disease progression.

## Supplementary information

Supplementary Material

Supplementary Table S1

Supplementary Data-Original Images for Blots

Supplementary Data-Cell images

Supplementary Data-Flow cytometry data

## Data Availability

The data supporting the findings of this study are available from the corresponding author upon reasonable request.
