# Supplementary material for: Mitochondrial DNA release via VDAC1 in keratinocytes: a key driver of innate immunity and vitiligo pathogenesis
Source: Cell Death Dis. 2026 Mar 18;17(1):318. doi: 10.1038/s41419-026-08585-5 (PMC13039960; doi:10.1038/s41419-026-08585-5)
Supplement: Supplementary file 1 — Supplementary Material [file 41419_2026_8585_MOESM1_ESM.docx]

**Supporting Information for Original article**

**Mitochondrial DNA Release via VDAC1 in Keratinocytes: A Key Driver of Innate Immunity and Vitiligo Pathogenesis**

**Supplementary Figures and Legends**





**Fig. S1. H_2_O_2_ treatment induces mtDNA release and activates the cGAS-STING pathway in NHKs.** **(A)** Cell viability measured by MTT assay after 24 h exposure to H_2_O_2_ (0-1000 μM). **(B)** Double-immunofluorescence labeling of dsDNA (green) and mitochondria (red) after 24 h H_2_O_2_ treatment. Scale bars, 50 μm. **(C-E)** Time-course analysis (4, 12, 24 h) of 500 μM H_2_O_2_-induced effects in NHKs. **(C-D)** Quantitative analysis of mtDNA (ATP6, ND1) in (C) cytosolic, and (D) whole-cell fractions by RT-qPCR. **(E)** Western blot analysis of CXCL9, CXCL10, CXCL16, cGAS, STING, phosphorylated NF-κB (p-NF-κB), protein expression. Data are presented as mean ± SD (n=3). Asterisks ^*^ indicate a significant difference exists between indicated groups, ^*^P<0.05, ^**^P<0.01, ^***^P<0.001. NHKs: normal human keratinocytes.


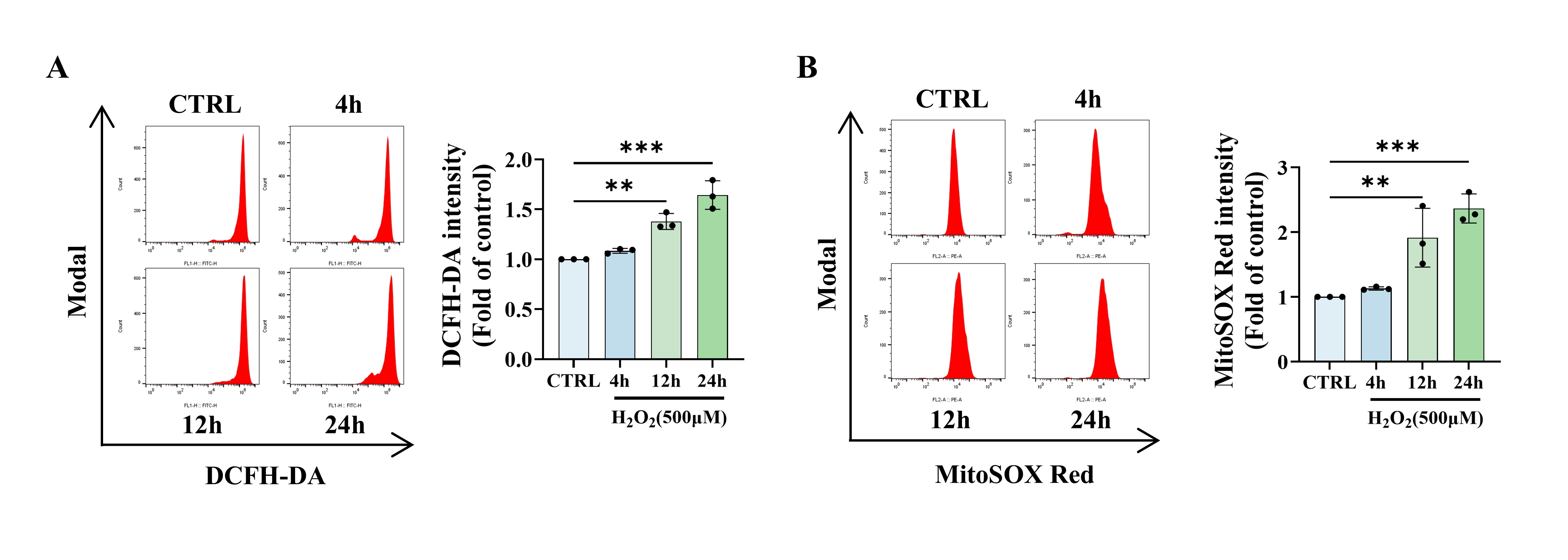


**Fig. S2. H_2_O_2_ treatment induces time-dependent accumulation of total and mitochondrial ROS in HaCaT cells.** **(A)** Quantification of **intracellular**ROS levels by flow cytometry using DCFH-DA in HaCaT cells treated with or without (CTRL) 500 μM H_2_O_2_ for the indicated times (4, 12, and 24 h). **(B)** Quantification of **mitochondrial superoxide** levels by flow cytometry using MitoSOX Red under the same treatment conditions as in (A). Data are presented as mean ± SD (n=3). Asterisks ^*^ indicate a significant difference exists between indicated groups, ^**^P<0.01, ^***^P<0.001.


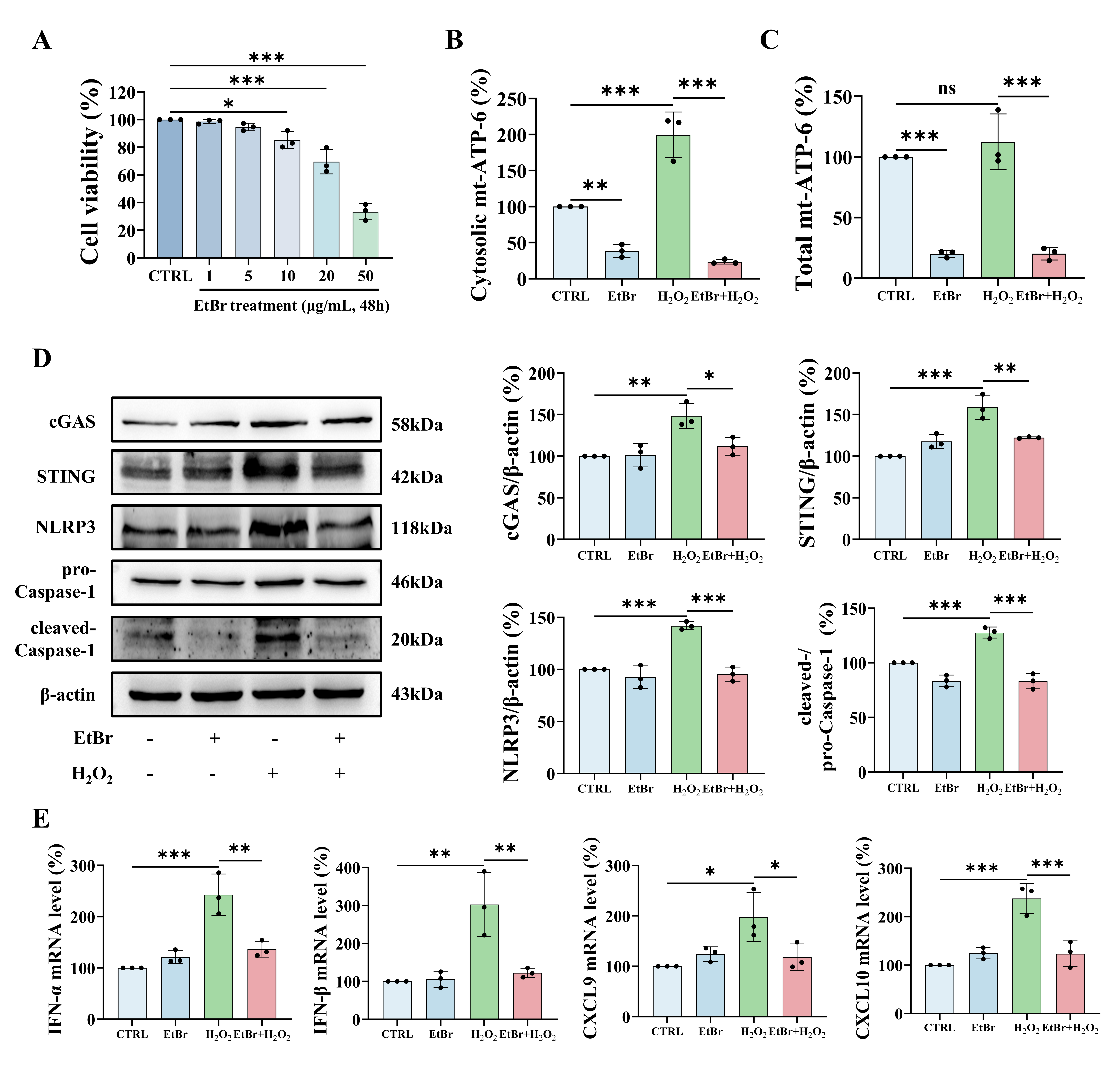


**Fig. S3. Cytosolic mtDNA reduction attenuates H_2_O_2_-induced immune responses in NHKs.** **(A)** Cytotoxicity assessment of EtBr (0-50 μg/mL, 48 h) by MTT assay. **(B-E)** NHKs were pre-treated with 0.2 μg/mL EtBr (48 h) followed by 500 μM H_2_O_2_ (24 h). **(B-C)** Quantitative analysis of mtDNA (ATP6) in (B) cytosolic, and (C) whole-cell fractions by RT-qPCR. **(D)** Western blot analysis of cGAS, STING, NLRP3 and cleaved Caspase-1 protein expression. **(E)** Transcript levels of IFN-α, IFN-β, CXCL9 and CXCL10 measured by RT-qPCR. Data are presented as mean ± SD (n=3). Asterisks ^*^ indicate a significant difference exists between indicated groups, ^*^P<0.05, ^**^P<0.01, ^***^P<0.001. NHKs: normal human keratinocytes, EtBr: ethidium bromide.


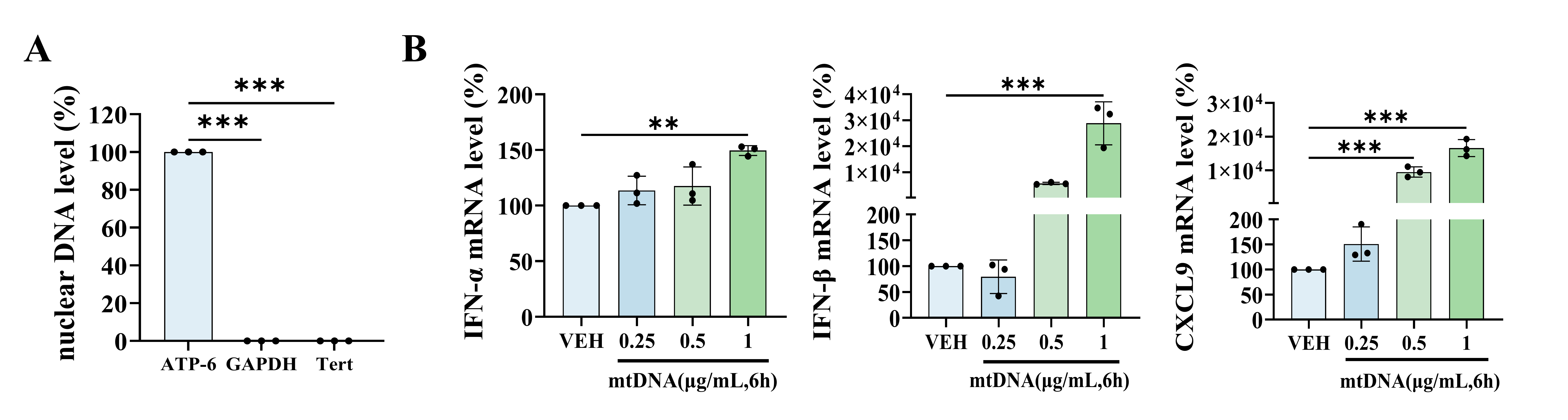


**Fig. S4. Validation of mtDNA purity and dose-response effects. (A)** Nuclear DNA contamination assessment (GAPDH and TERT normalized to mtDNA-ATP6 by RT-qPCR). **(B)** Transcript levels of IFN-α/β and CXCL9 transfected with mtDNA (0.25, 0.5, 1 μg/mL) for 6 h. Data are presented as mean ± SD (n=3). Asterisks ^*^ indicate a significant difference exists between indicated groups, ^**^P<0.01, ^***^P<0.001.


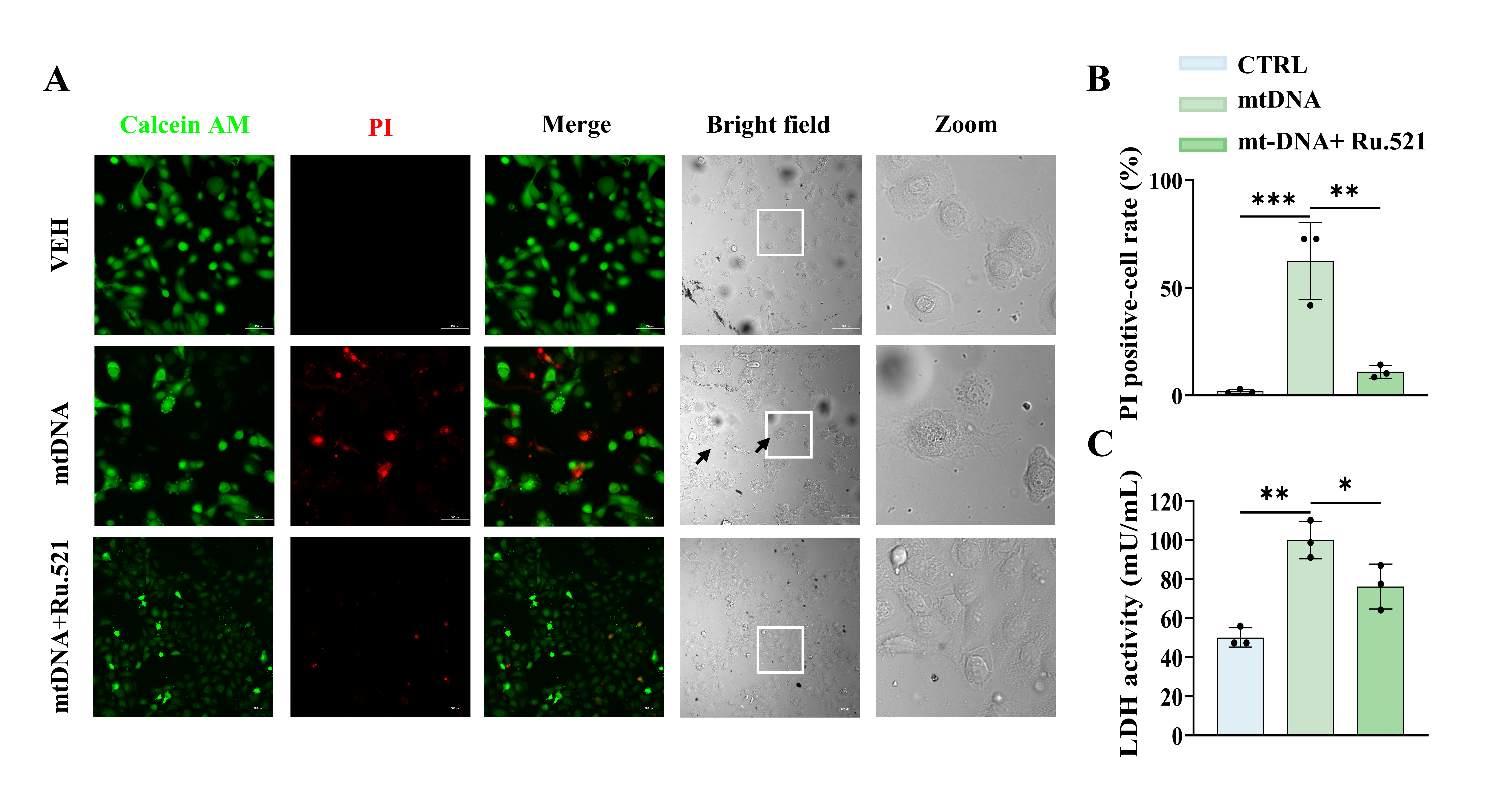


**Fig. S5. Cytosolic mtDNA induces pyroptosis in NHKs.** **(A-C)** NHKs transfected with 1 μg/mL mtDNA ± 10 μM RU.521 (cGAS inhibitor, 1 h pretreatment). **(A)** Representative image of PI/Calcein AM staining. Scale bars, 100 μm. **(B)** Quantification of PI-positive cells in three randomly chosen fields (one field per well). **(C)** LDH release in culture supernatants. Data are presented as mean ± SD (n=3). Asterisks ^*^ indicate a significant difference exists between indicated groups, ^*^P<0.05, ^**^P<0.01, ^***^P<0.001. VEH: vehicle, PI: propidium iodide, LDH: lactate dehydrogenase.





**Fig. S6. VDAC1 oligomerization and mPTP opening mediate H_2_O_2_-induced mtDNA release in NHKs. (A)** Immunofluorescence of Calcein AM staining in (i) CTRL, (ii) H_2_O_2_-only, (iii) H_2_O_2_+ CsA, and (iv) H_2_O_2_+ VBIT-4 groups. Scale bars, 50 μm. Fluorescence intensity was normalized relative to the CTRL group. **(B)** Flow cytometry analysis of cell apoptosis after H_2_O_2_ stimulation (500 μM, 24 h). **(C)** Western blot analysis of VDAC1 oligomerization after H_2_O_2_ treatment (500 μM, 24 h). Asterisk indicates a nonspecific band. **(D)** Quantification of mtDNA level (ATP6) in the IMS by RT-qPCR. **(E-F)** Quantitative analysis of mtDNA (ATP6) in (E) cytosolic, and (F) whole-cell fractions by RT-qPCR. Data are presented as mean ± SD (n=3). Asterisks ^*^ indicate a significant difference exists between indicated groups, ^*^P<0.05, ^**^P<0.01, ^***^P<0.001. CsA: cyclosporin A, mPTP: mitochondrial permeability transition pore, IMS: mitochondrial intermembrane space, IMM: inner mitochondrial membrane, OMM: outer mitochondrial membrane.
