# Supplementary material for: Mitochondrial DNA release via VDAC1 in keratinocytes: a key driver of innate immunity and vitiligo pathogenesis
Source: Cell Death Dis. 2026 Mar 18;17(1):318. doi: 10.1038/s41419-026-08585-5 (PMC13039960; doi:10.1038/s41419-026-08585-5)
Supplement: Supplementary file 2 — Supplementary Table S1 [file 41419_2026_8585_MOESM2_ESM.docx]

**Table S1. Primer sequences for quantitative real-time PCR**

| Primer | Sequence |
| --- | --- |
| Human GAPDH forward | GGACCTGACCTGCCGTCTAG |
| Human GAPDH reverse | GTAGCCCAGGATGCCCTTGA |
| Human Tert forward | CTGTCGGAAGCAGAGGTCAG |
| Human Tert reverse | TGAACAGTGCCTTCACCCTC |
| Human ATP-6 forward | ACCAATAGCCCTGGCCGTAC |
| Human ATP-6 reverse | GGTGGCGCTTCCAATTAGGT |
| Human MT-ND1 forward | CTCTTCGTCTGATCCGTCCT |
| Human MT-ND1 reverse | TGAGGTTGCGGTCTGTTAGT |
| Human MT-ND2 forward | GTAGACAGTCCCACCCTCAC |
| Human MT-ND2 reverse | TTGATCCCGTTTCGTGCAAG |
| Human MT-CO1 forward | AACCCAATACCAAACGCCCC |
| Human MT-CO1 reverse | GTGTTGAGGTTGCGGTCTGT |
| Human IFN-α forward | GAAATACAGCCCTTGTGCCTG |
| Human IFN-α reverse | AGTTATAGCAGGGGTGAGAGT |
| Human IFN-β forward | TGGCACAACAGGTAGTAGGC |
| Human IFN-β reverse | AGTGGAGAAGCACAACAGGAG |
| Human IFN-γ forward | TGAATGTCCAACGCAAAGCA |
| Human IFN-γ reverse | ACTGGGATGCTCTTCGACCT |
| Human CXCL9 forward | TGTTATGGGCAGGATGGCAA |
| Human CXCL9 reverse | CCCTGGTCCCTGTAGTGAGT |
| Human CXCL10 forward | GTGGATGTTCTGACCCTGCT |
| Human CXCL10 reverse | GGAGGATGGCAGTGGAAGTC |
| Human IL-6 forward | TGAGGAGACTTGCCTGGTGAA |
| Human IL-6 reverse | CAGCTCTGGCTTGTTCCTCAC |
| Human IL-1β forward | ACAGATGAAGTGCTCCTTCCA |
| Human IL-1β reverse | GTCGGAGATTCGTAGCTGGAT |
