# Supplementary material for: Mitochondrial DNA release via VDAC1 in keratinocytes: a key driver of innate immunity and vitiligo pathogenesis
Source: Cell Death Dis. 2026 Mar 18;17(1):318. doi: 10.1038/s41419-026-08585-5 (PMC13039960; doi:10.1038/s41419-026-08585-5)
Supplement: Supplementary file 3 — Supplementary Data-Original Images for Blots [file 41419_2026_8585_MOESM3_ESM.pdf]

Figure 1F

HaCaT

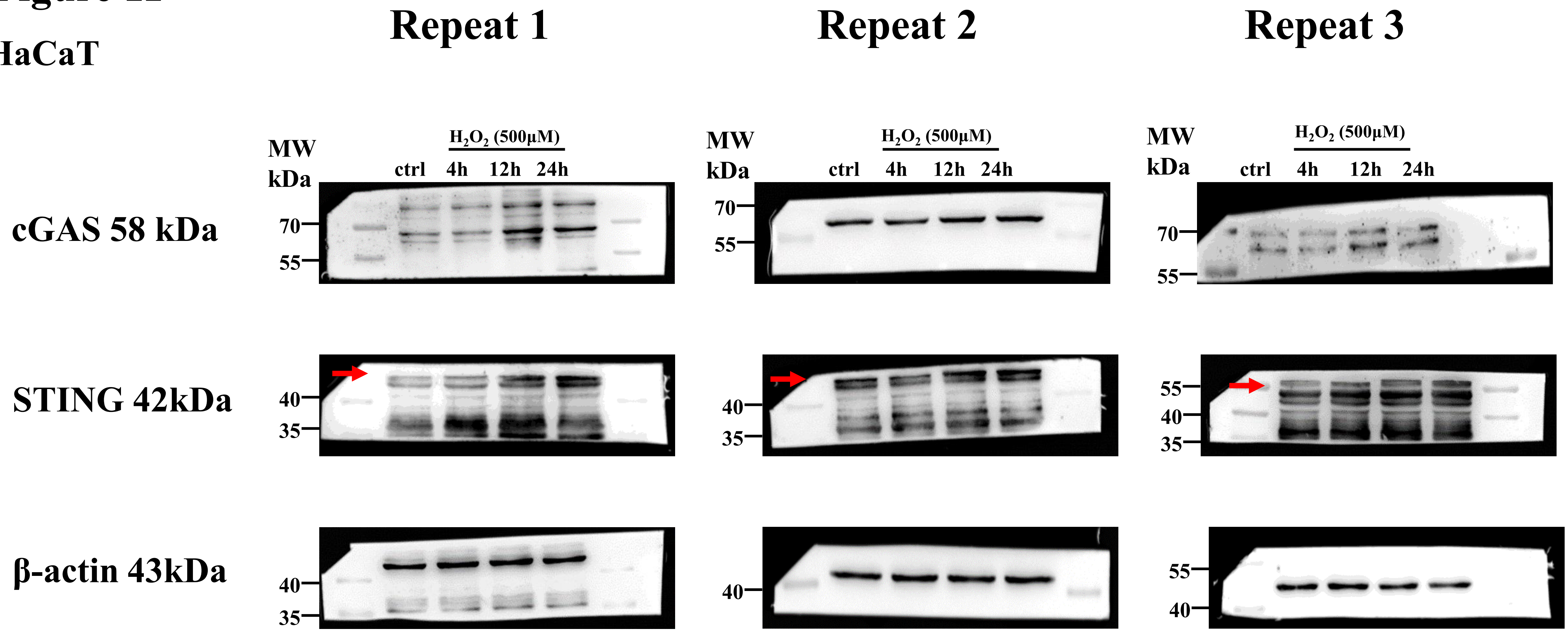

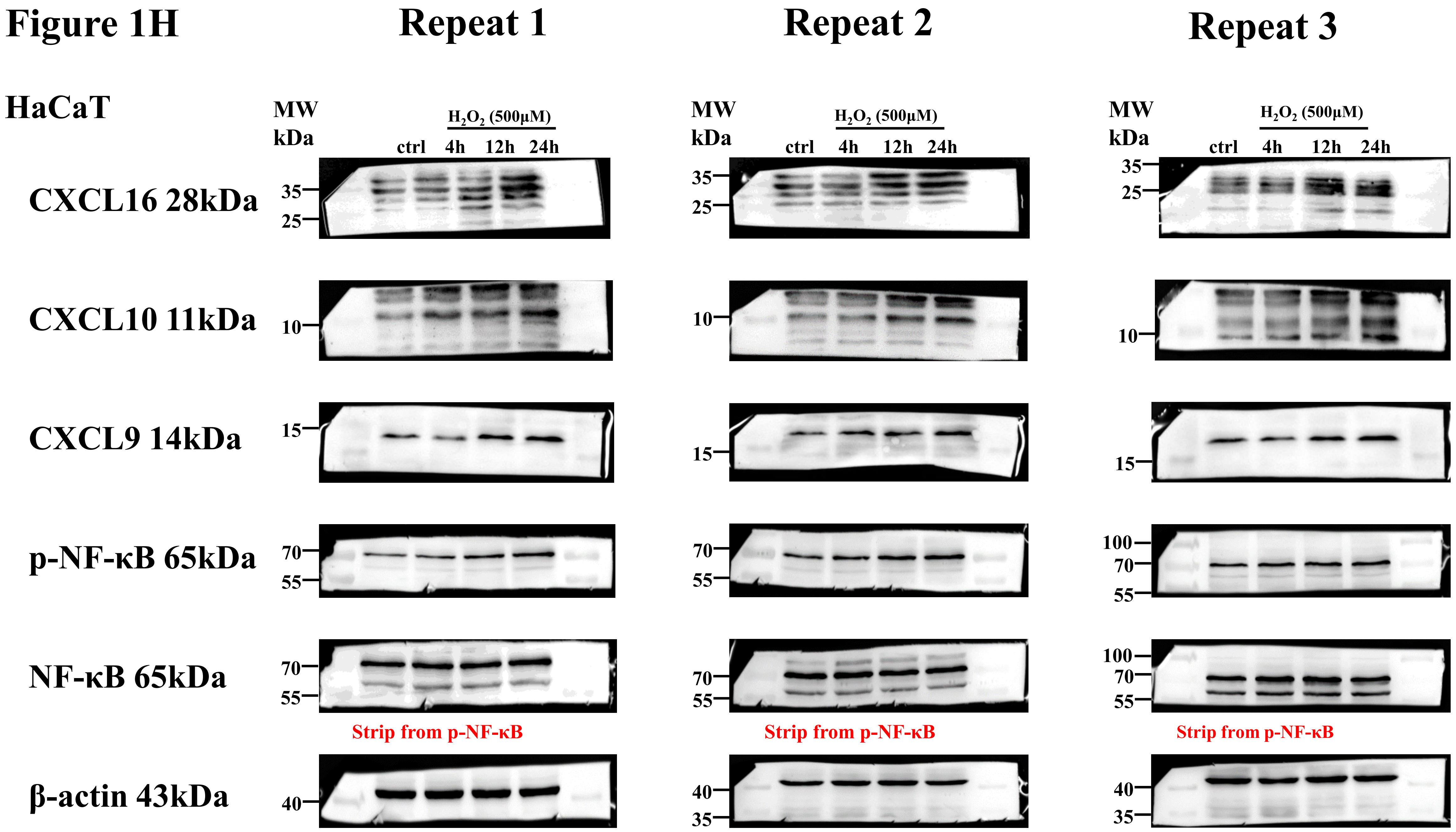
Figure 1H
Repeat 1
Repeat 2
Repeat 3
HaCaT
MW kDa
H_2O_2 (500μM)
ctrl 4h 12h 24h
CXCL16 28kDa
CXCL10 11kDa
CXCL9 14kDa
p-NF-κB 65kDa
NF-κB 65kDa
β-actin 43kDa
Strip from p-NF-κB
Strip from p-NF-κB
Strip from p-NF-κB
35
25
10
15
100
70
55
40
35

Figure S1E

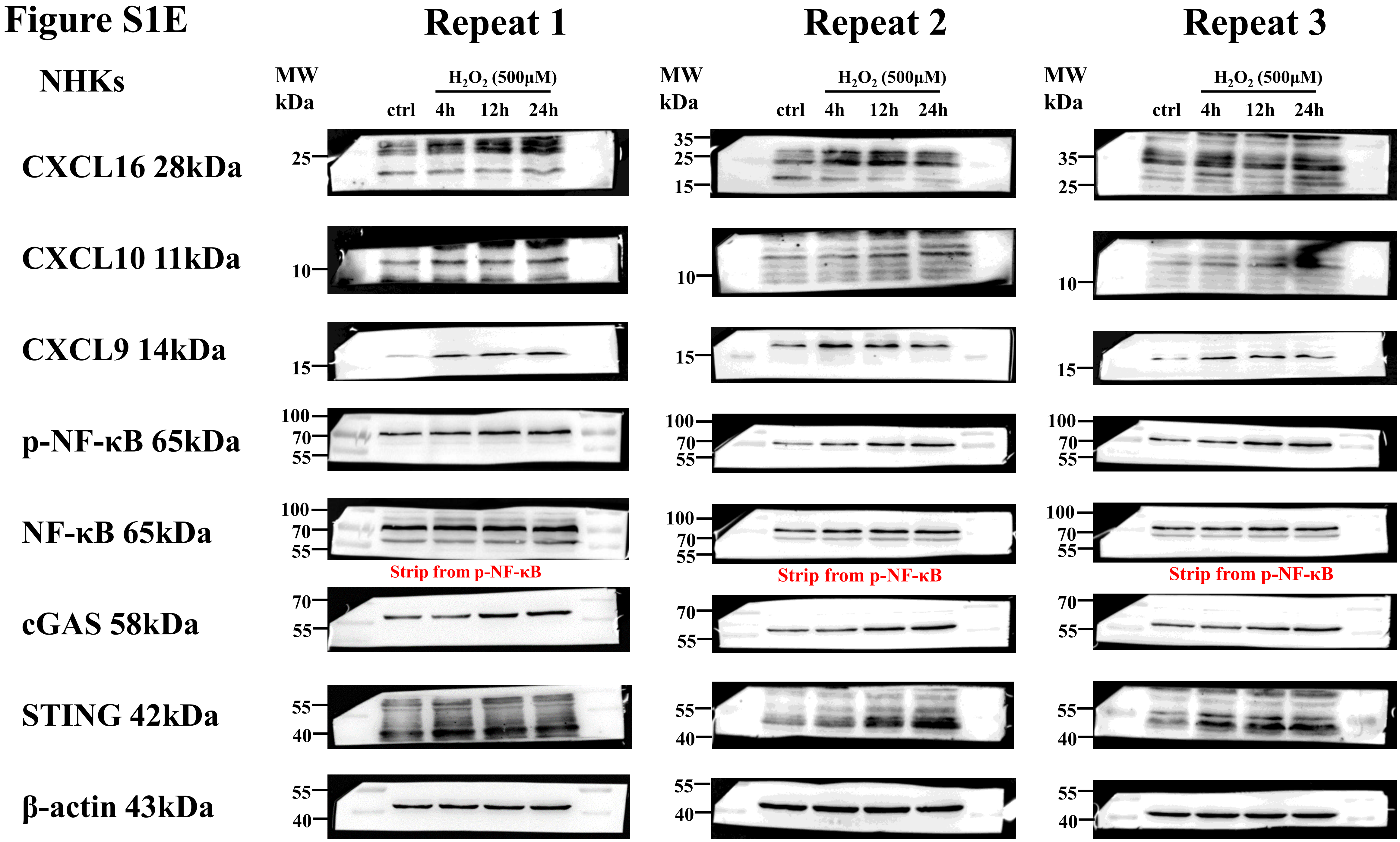
Repeat 1
Repeat 2
Repeat 3
NHKs
MW kDa
H2O2 (500μM)
ctrl
4h
12h
24h
CXCL16 28kDa
CXCL10 11kDa
CXCL9 14kDa
p-NF-κB 65kDa
NF-κB 65kDa
cGAS 58kDa
STING 42kDa
β-actin 43kDa
Strip from p-NF-κB
35
25
15
10
100
70
55
40

Figure 2D

HaCaT

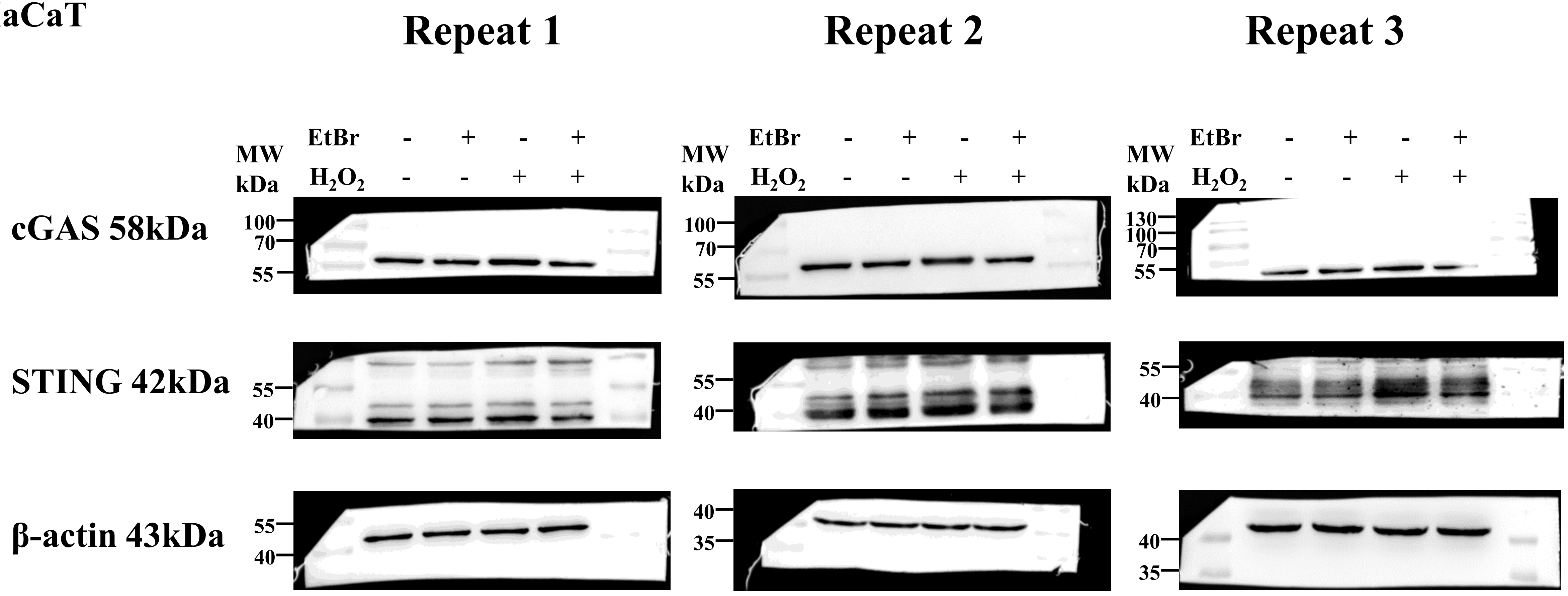

Figure 2F

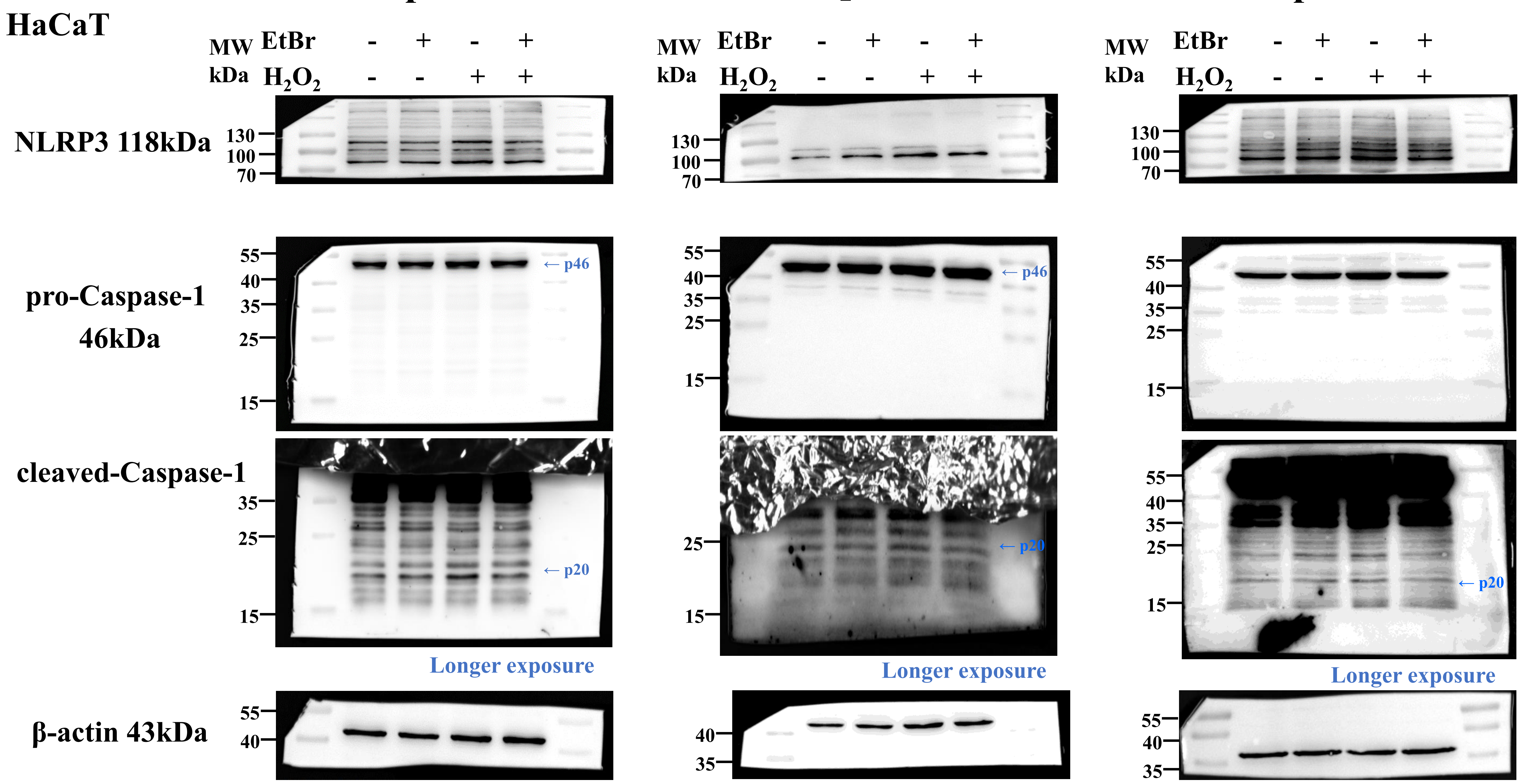
HaCaT
Repeat 1
Repeat 2
Repeat 3
MW kDa
EtBr - + - +
H_2O_2 - - + +
NLRP3 118kDa
130
100
70
pro-Caspase-1 46kDa
55
40
35
25
15
← p46
cleaved-Caspase-1
← p20
Longer exposure
β-actin 43kDa

Figure S3D

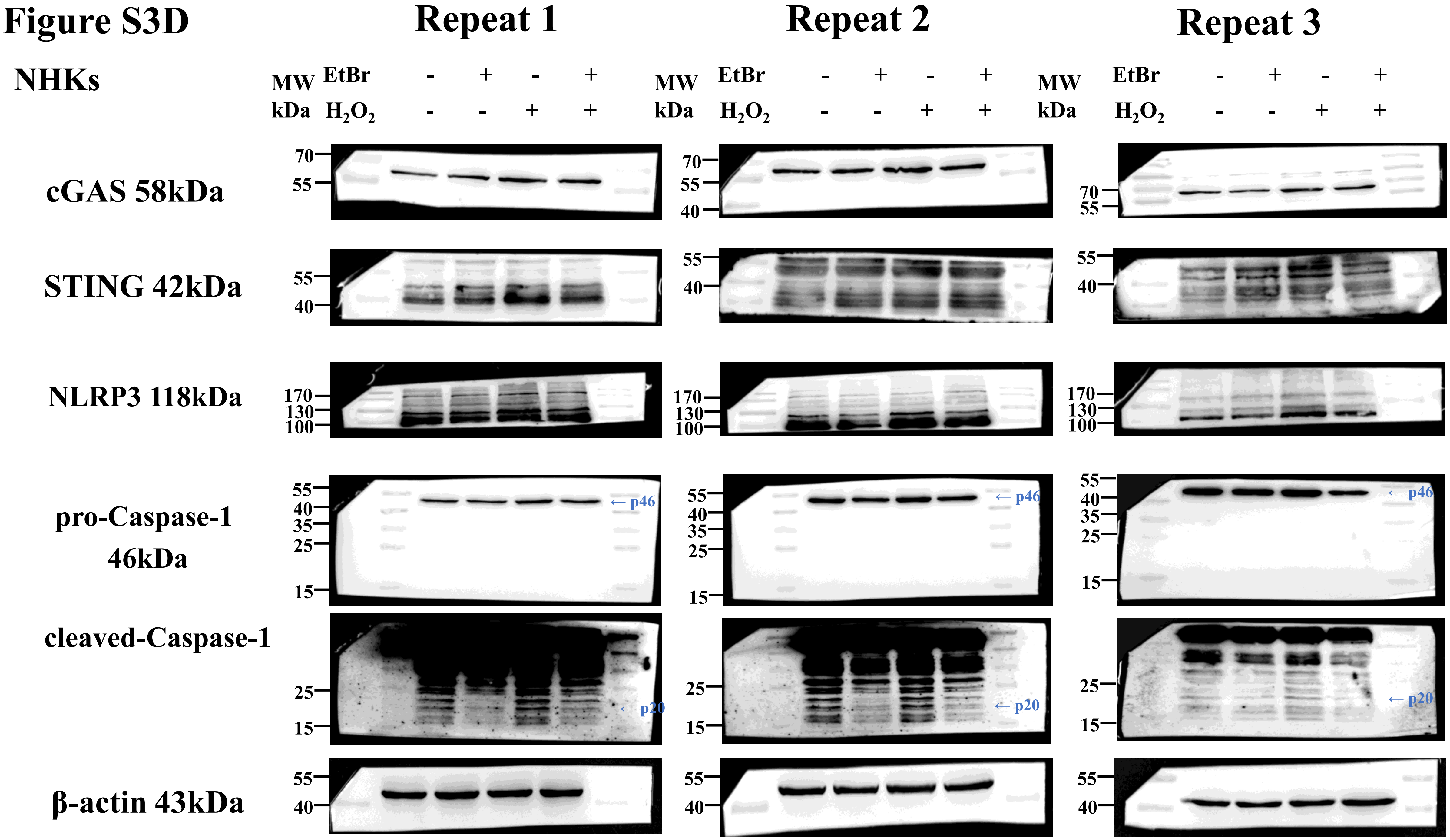

Repeat 1
Repeat 2
Repeat 3
NHKs
MW kDa
EtBr - + - +
H_2O_2 - - + +
cGAS 58kDa
STING 42kDa
NLRP3 118kDa
pro-Caspase-1 46kDa
cleaved-Caspase-1
β-actin 43kDa
70
55
40
170
130
100
35
25
15
← p46
← p20

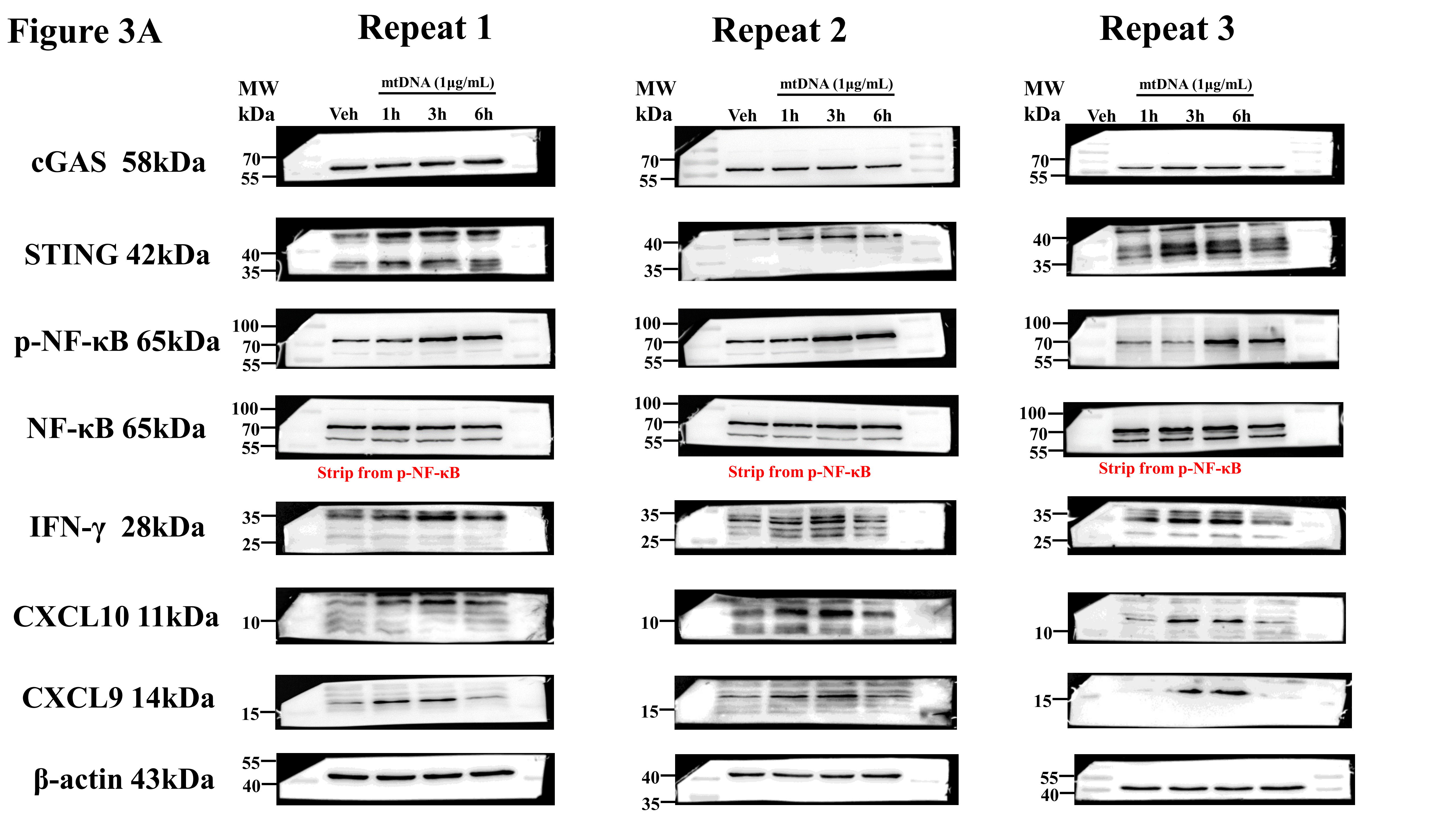
Figure 3A
Repeat 1
Repeat 2
Repeat 3
MW kDa
mtDNA (1μg/mL)
Veh
1h
3h
6h
cGAS 58kDa
STING 42kDa
p-NF-κB 65kDa
NF-κB 65kDa
Strip from p-NF-κB
IFN-γ 28kDa
CXCL10 11kDa
CXCL9 14kDa
β-actin 43kDa
100
70
55
40
35
25
15
10

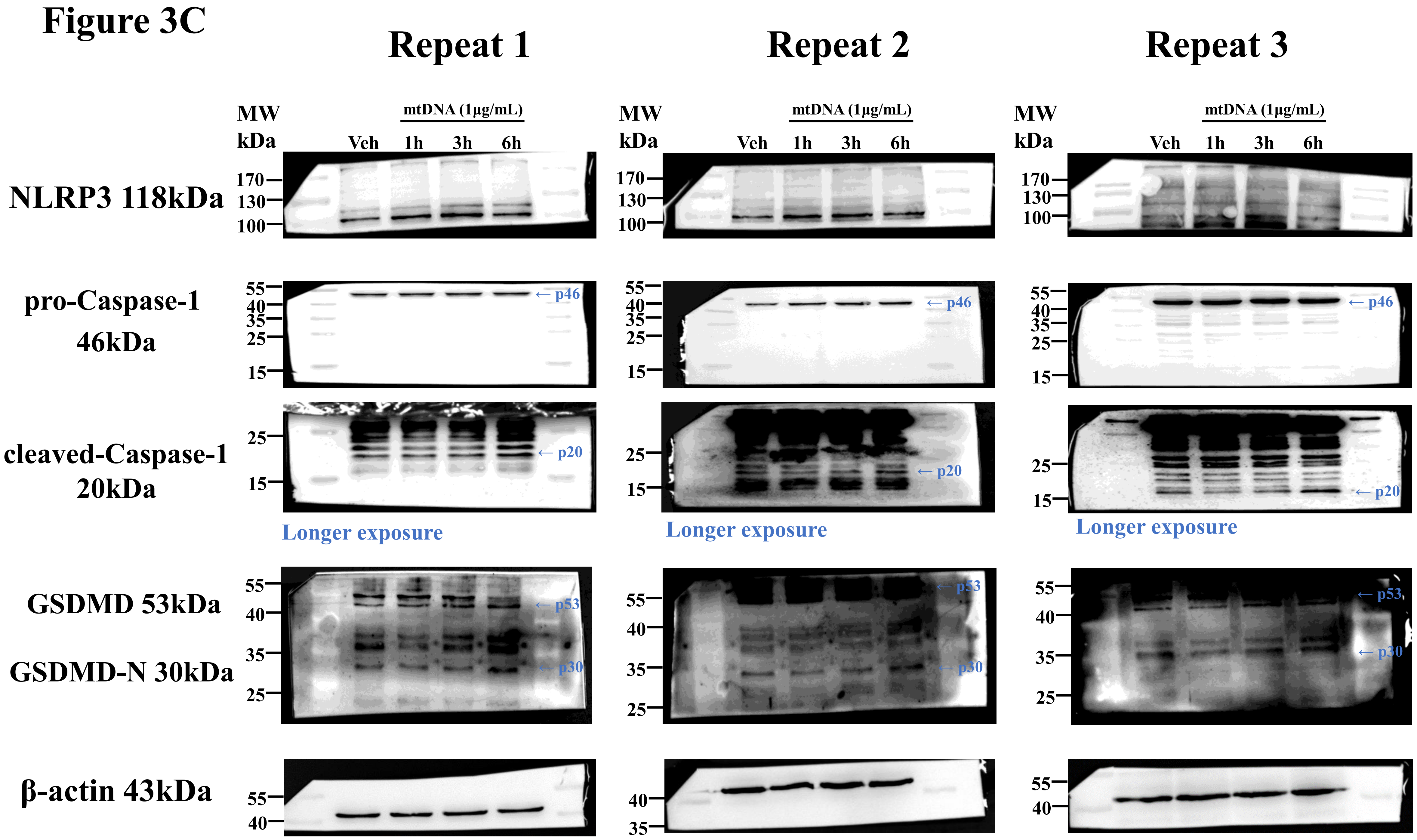
Figure 3C
Repeat 1
Repeat 2
Repeat 3
MW kDa
mtDNA (1μg/mL)
Veh
1h
3h
6h
NLRP3 118kDa
pro-Caspase-1 46kDa
cleaved-Caspase-1 20kDa
GSDMD 53kDa
GSDMD-N 30kDa
β-actin 43kDa
170
130
100
55
40
35
25
15
← p46
← p20
← p53
← p30
Longer exposure

Figure 4A

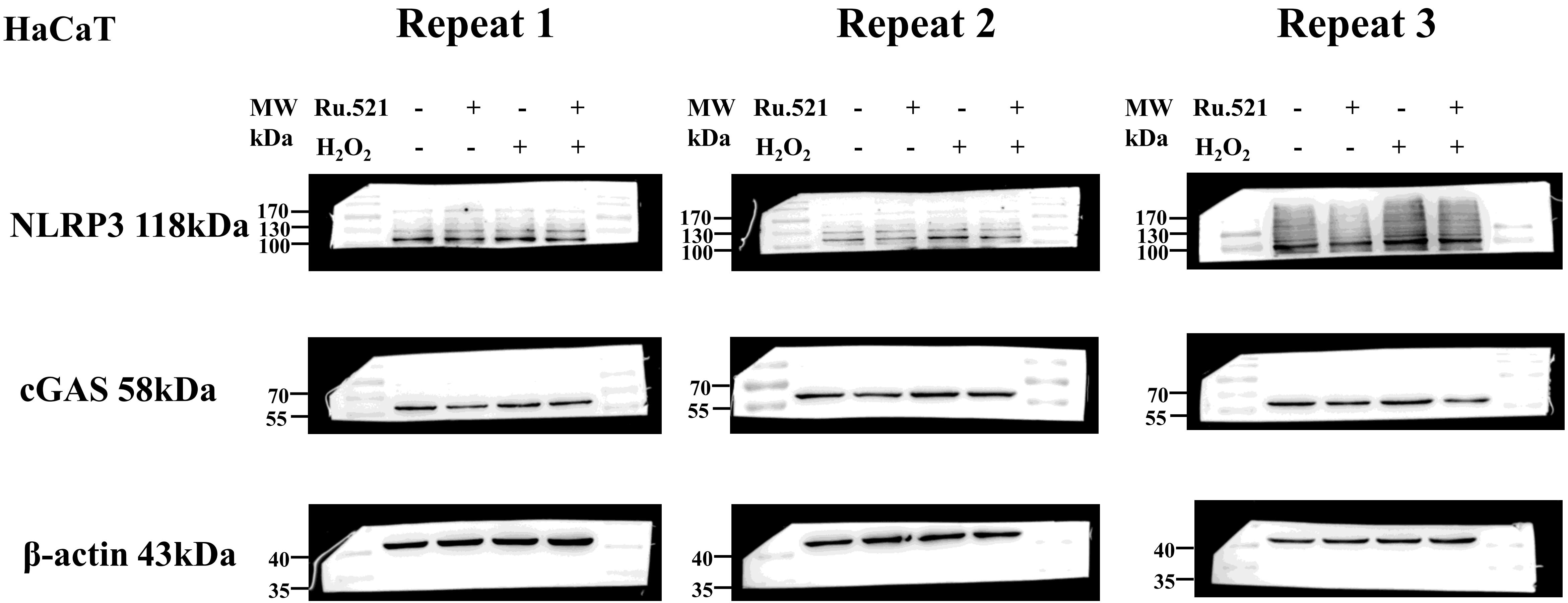
HaCaT
Repeat 1
Repeat 2
Repeat 3
MW kDa
Ru.521 - + - +
H_2O_2 - - + +
NLRP3 118kDa
170
130
100
cGAS 58kDa
70
55
β-actin 43kDa
40
35

Figure 4B

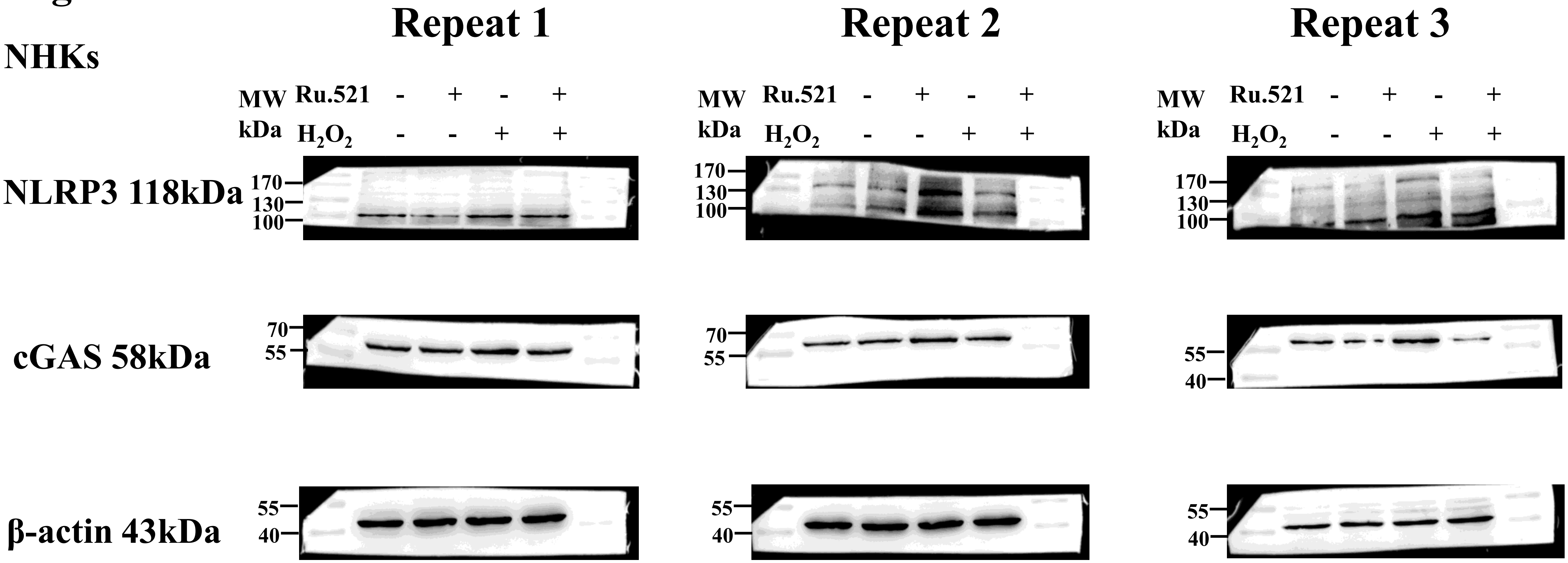
Repeat 1
Repeat 2
Repeat 3
NHKs
MW kDa
Ru.521 - + - +
H_2O_2 - - + +
NLRP3 118kDa
170
130
100
cGAS 58kDa
70
55
40
β-actin 43kDa
55
40

Figure 4C

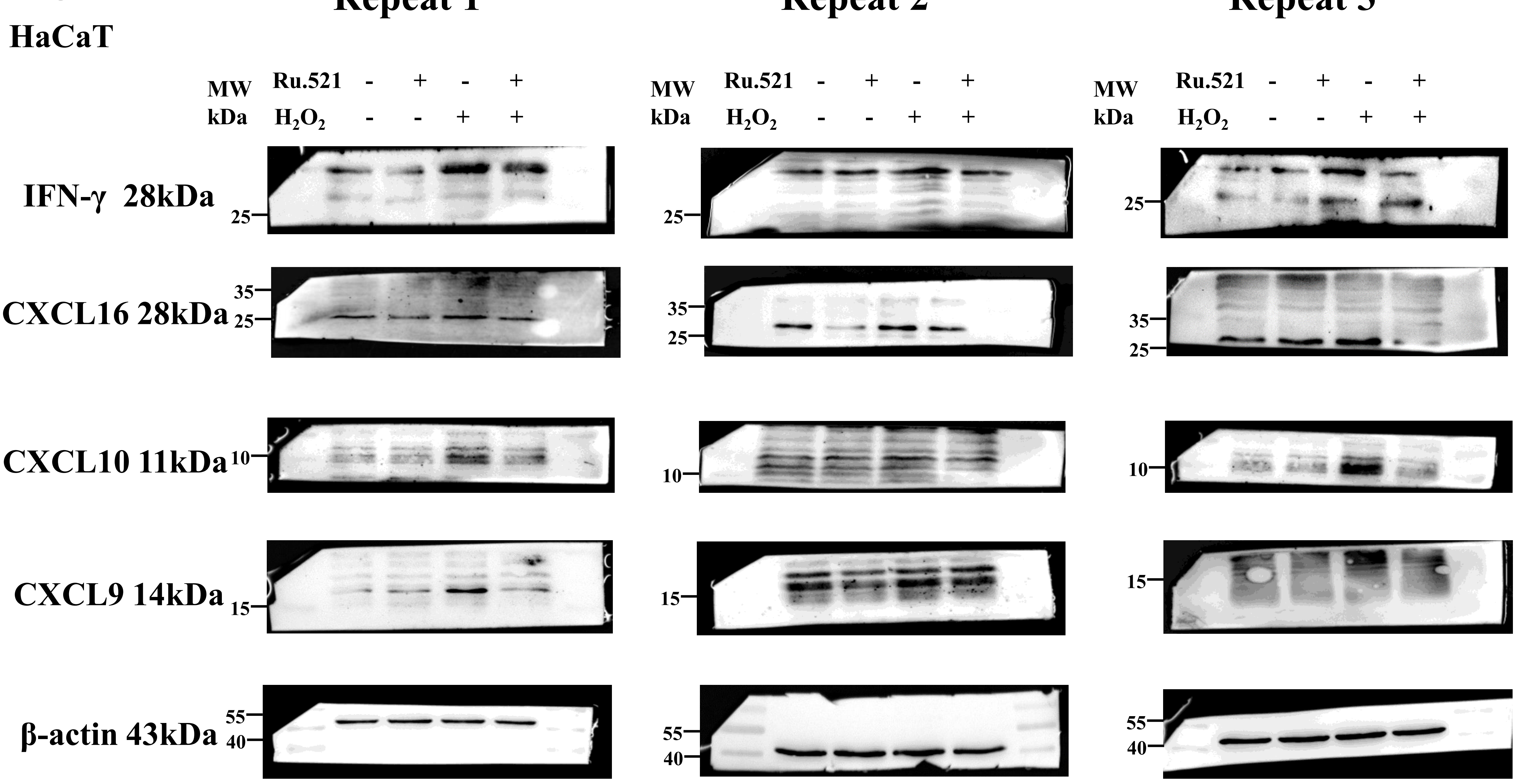

Repeat 1
Repeat 2
Repeat 3
HaCaT
MW kDa
Ru.521 - + - +
H2O2 - - + +
IFN-γ 28kDa
CXCL16 28kDa
CXCL10 11kDa
CXCL9 14kDa
β-actin 43kDa
25
35
10
15
55
40

Figure 4D

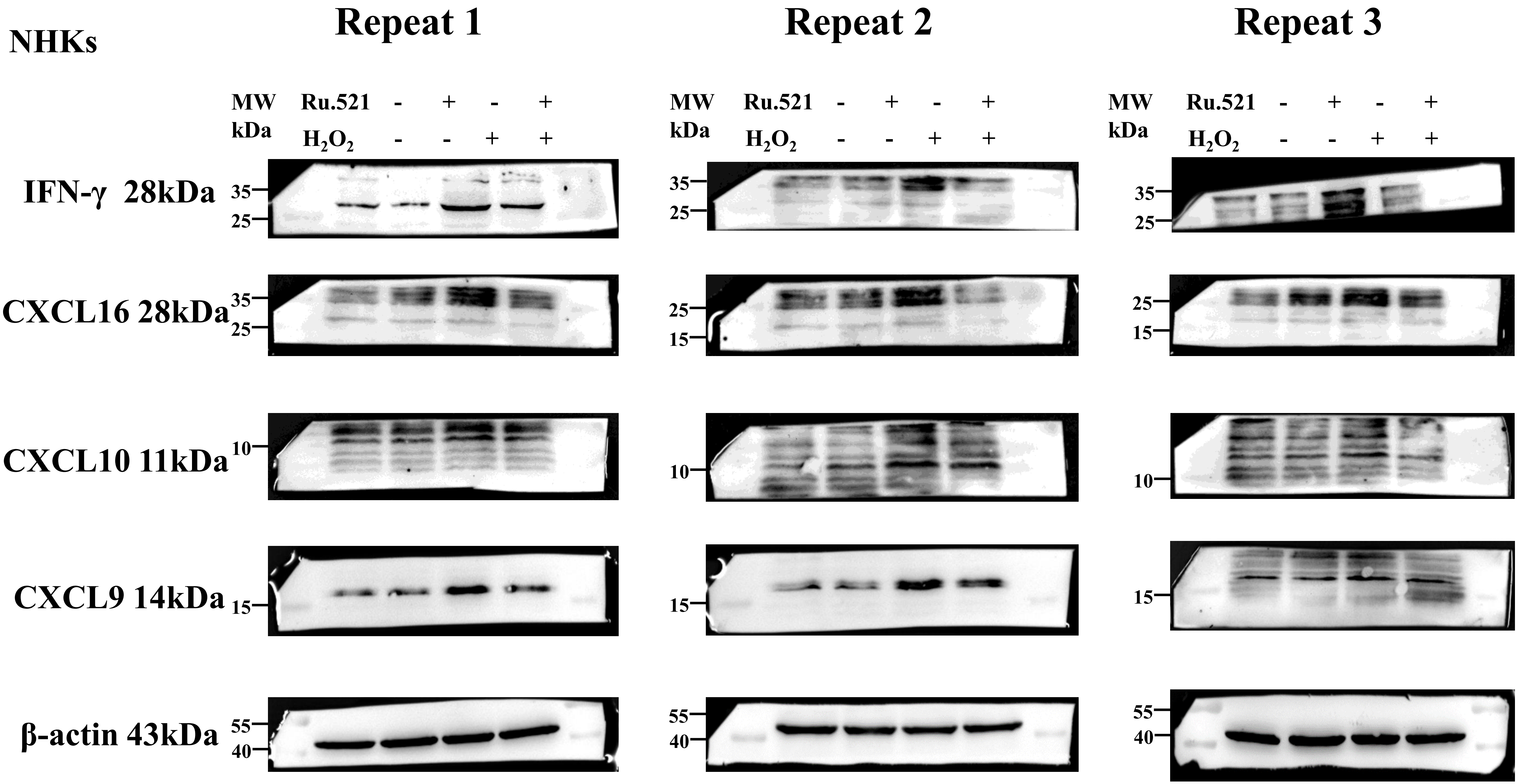
NHKs
Repeat 1
Repeat 2
Repeat 3
MW kDa
Ru.521 - + - +
H2O2 - - + +
IFN-γ 28kDa
CXCL16 28kDa
CXCL10 11kDa
CXCL9 14kDa
β-actin 43kDa
35
25
15
10
55
40

Figure 5C

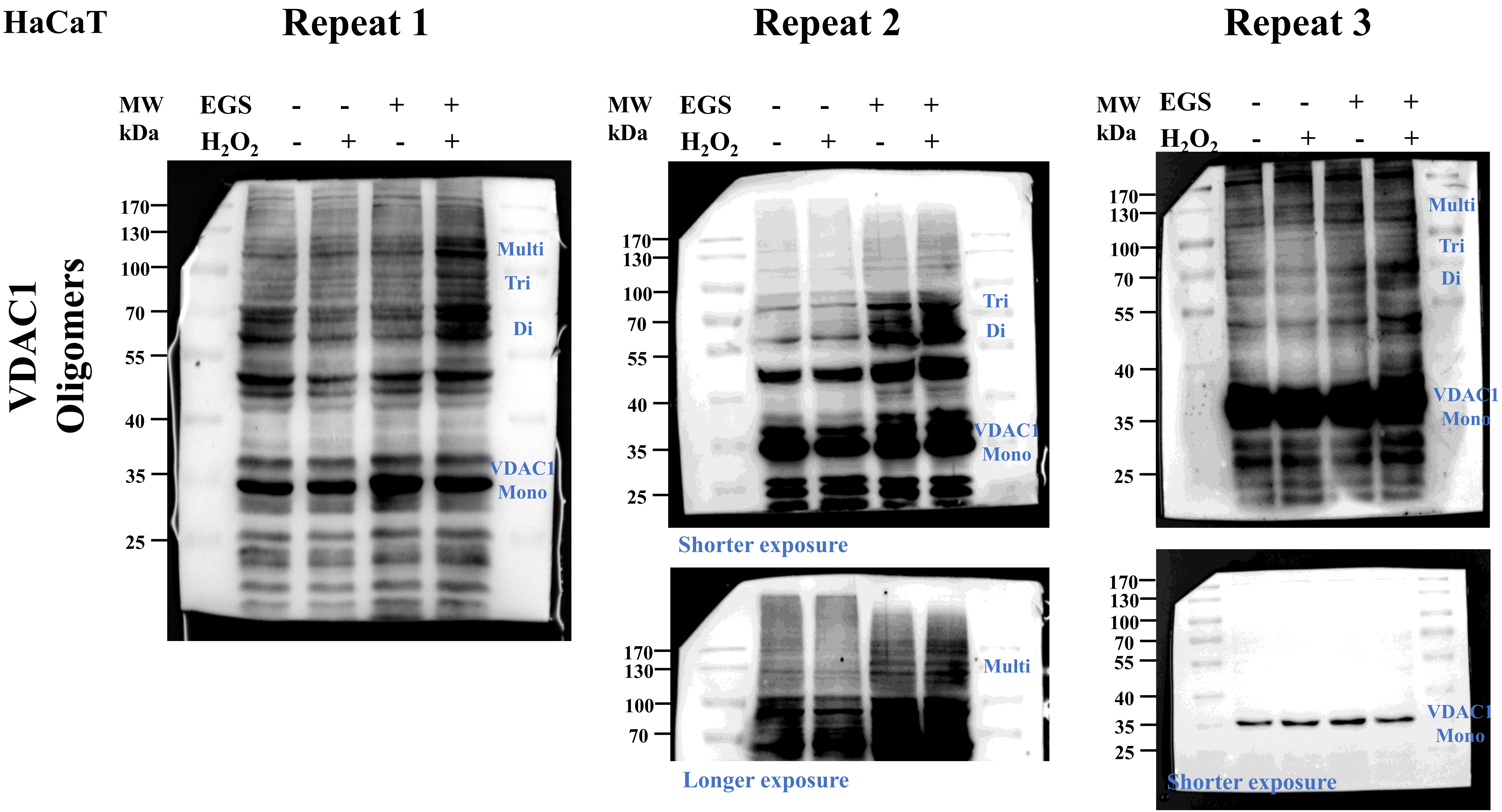
HaCaT
Repeat 1
Repeat 2
Repeat 3
VDAC1 Oligomers
MW kDa
EGS - - + +
H_2O_2 - + - +
170
130
100
70
55
40
35
25
Multi
Tri
Di
VDAC1 Mono
Shorter exposure
Longer exposure

Figure S6C

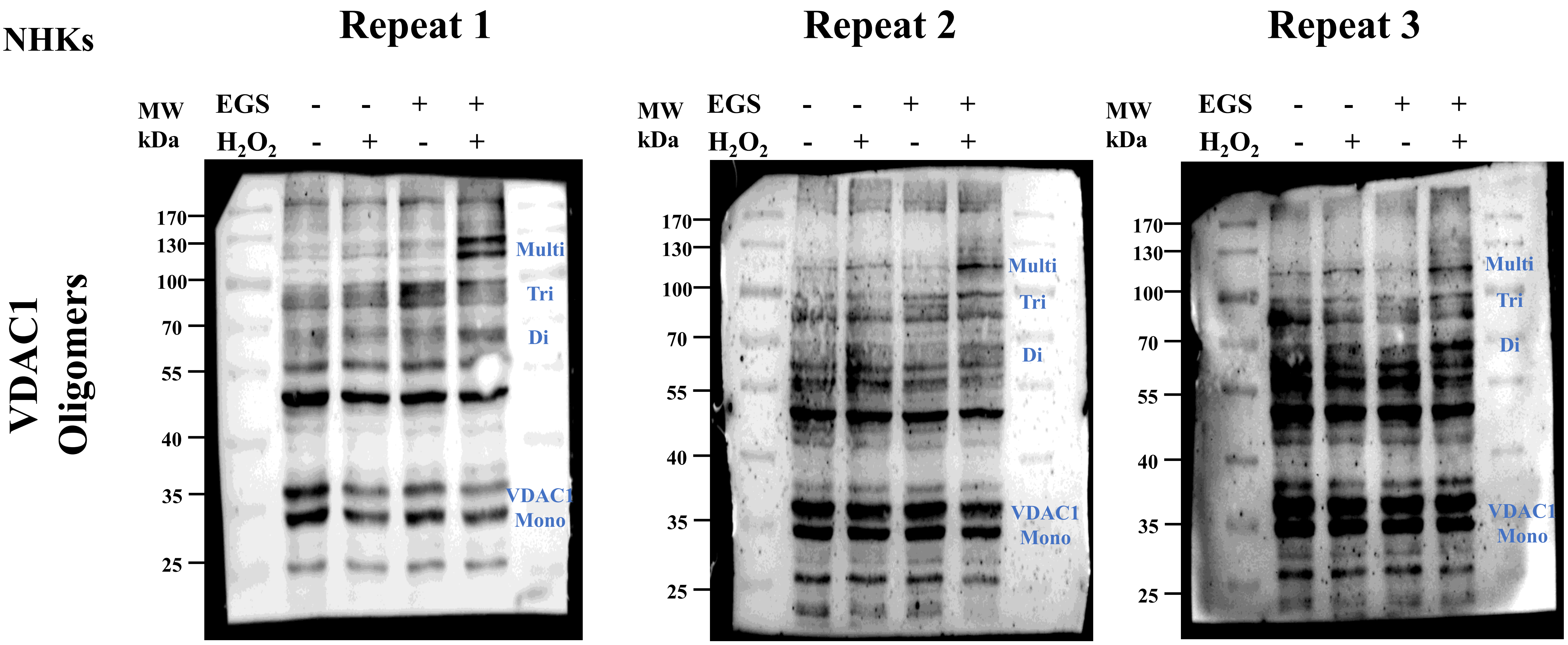
NHKs
Repeat 1
Repeat 2
Repeat 3
VDAC1
Oligomers
MW
kDa
EGS - - + +
H_2O_2 - + - +
170
130
100
70
55
40
35
25
Multi
Tri
Di
VDAC1
Mono

Figure 6A

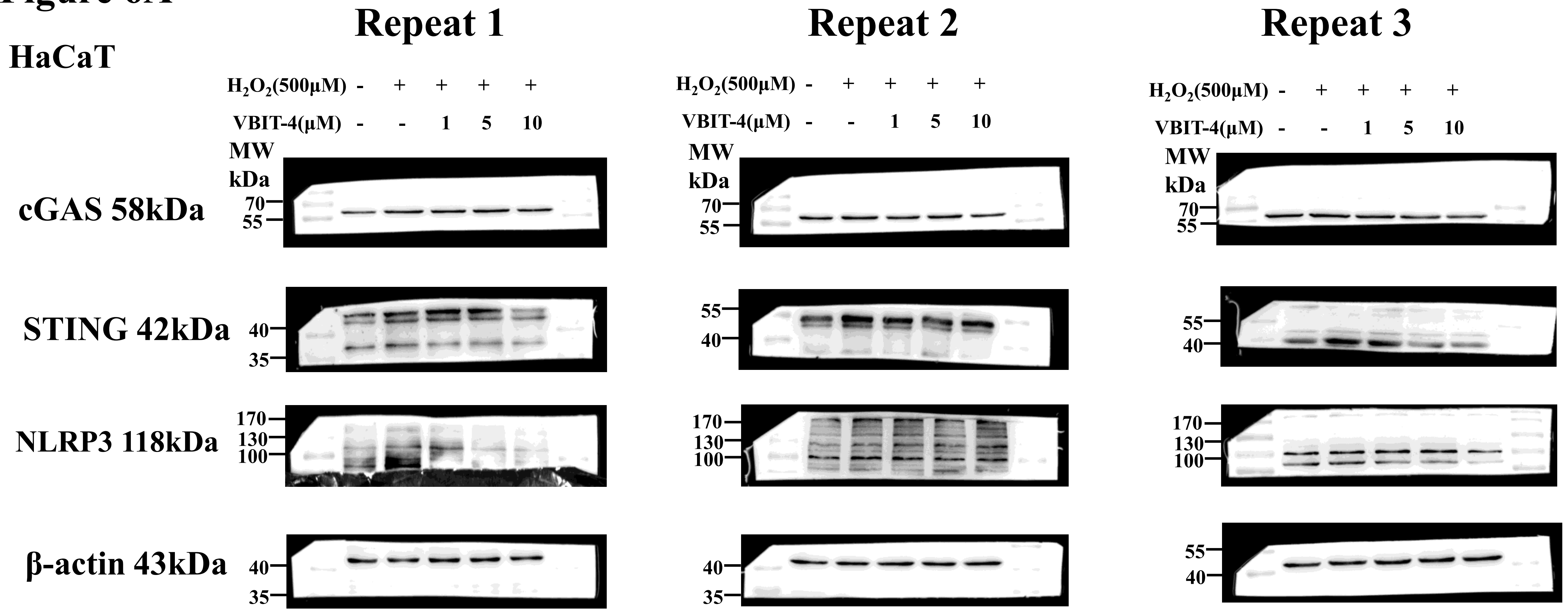
Repeat 1
Repeat 2
Repeat 3
HaCaT
H_2O_2(500μM) - + + + +
VBIT-4(μM) - - 1 5 10
MW
kDa
cGAS 58kDa
STING 42kDa
NLRP3 118kDa
β-actin 43kDa
70
55
40
35
170
130
100

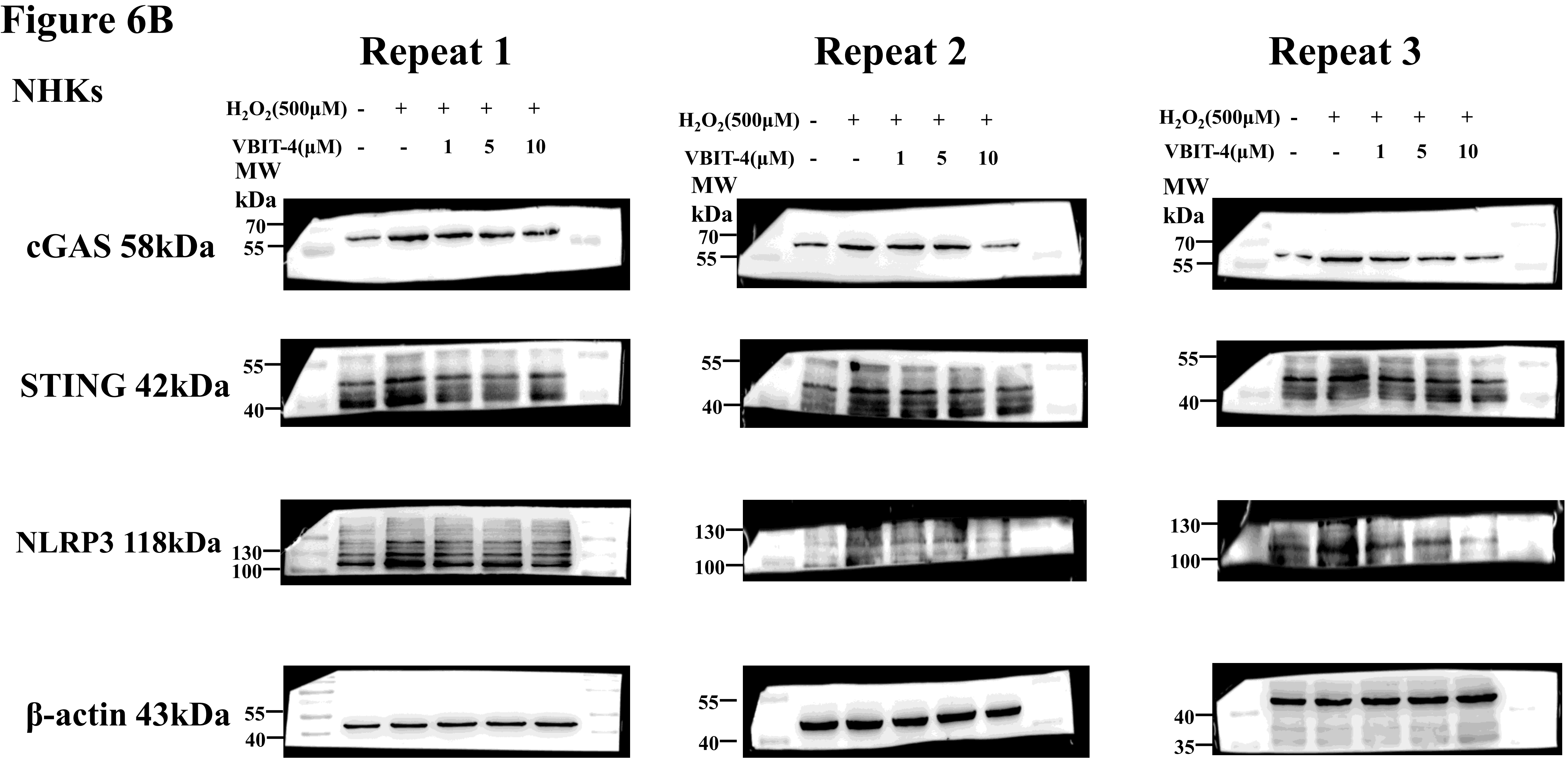
Figure 6B
Repeat 1
Repeat 2
Repeat 3
NHKs
H2O2(500μM) - + + + +
VBIT-4(μM) - - 1 5 10
MW
kDa
cGAS 58kDa
STING 42kDa
NLRP3 118kDa
β-actin 43kDa
70
55
40
130
100
35

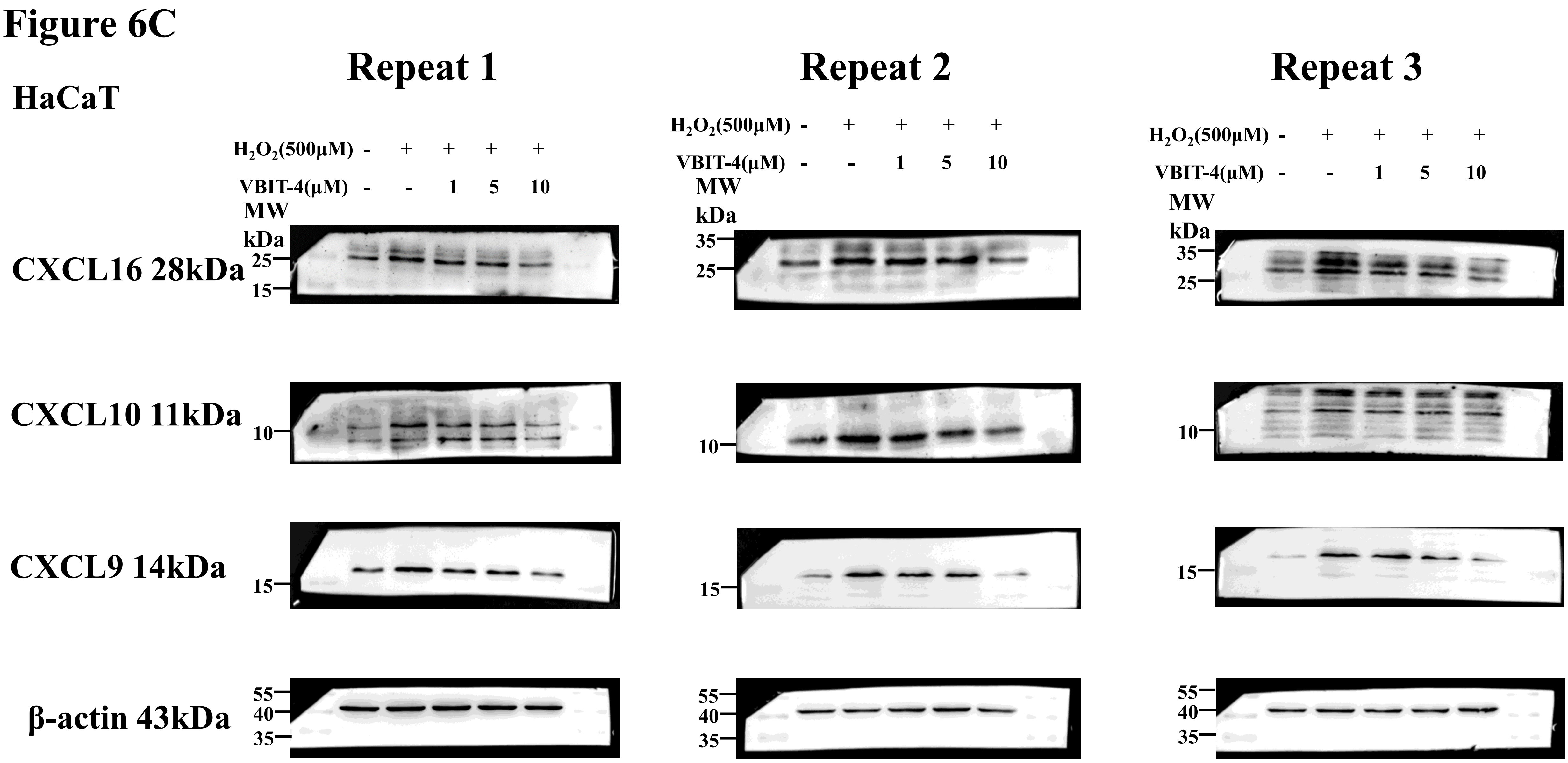
Figure 6C
HaCaT
Repeat 1
Repeat 2
Repeat 3
H2O2(500μM) - + + + +
VBIT-4(μM) - - 1 5 10
MW kDa
CXCL16 28kDa
CXCL10 11kDa
CXCL9 14kDa
β-actin 43kDa
35
25
15
10
55
40

Figure 6D

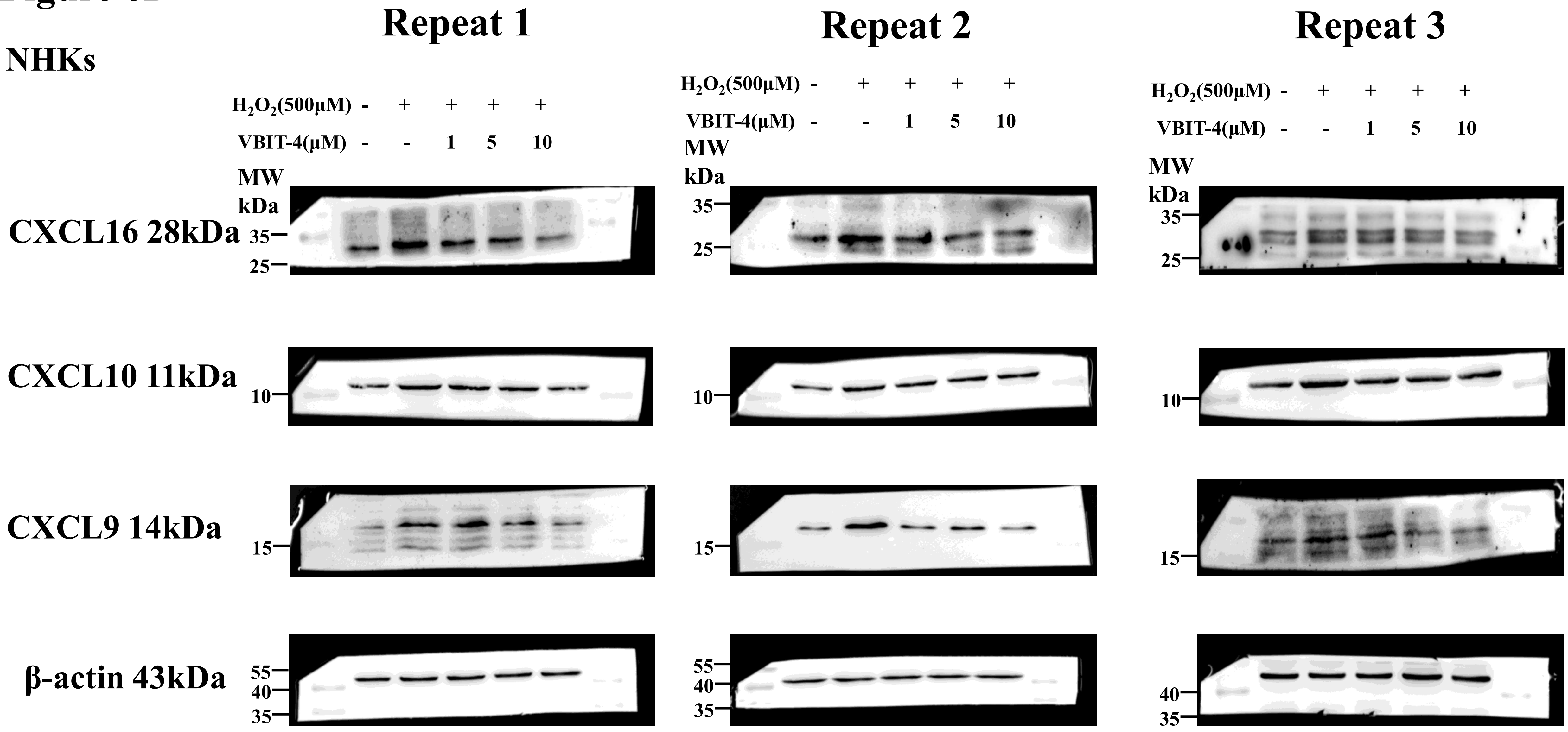

Repeat 1
Repeat 2
Repeat 3
NHKs
H2O2(500μM) - + + + +
VBIT-4(μM) - - 1 5 10
MW kDa
CXCL16 28kDa
35
25
CXCL10 11kDa
10
CXCL9 14kDa
15
β-actin 43kDa
55
40
35

Figure 6E

NHKs

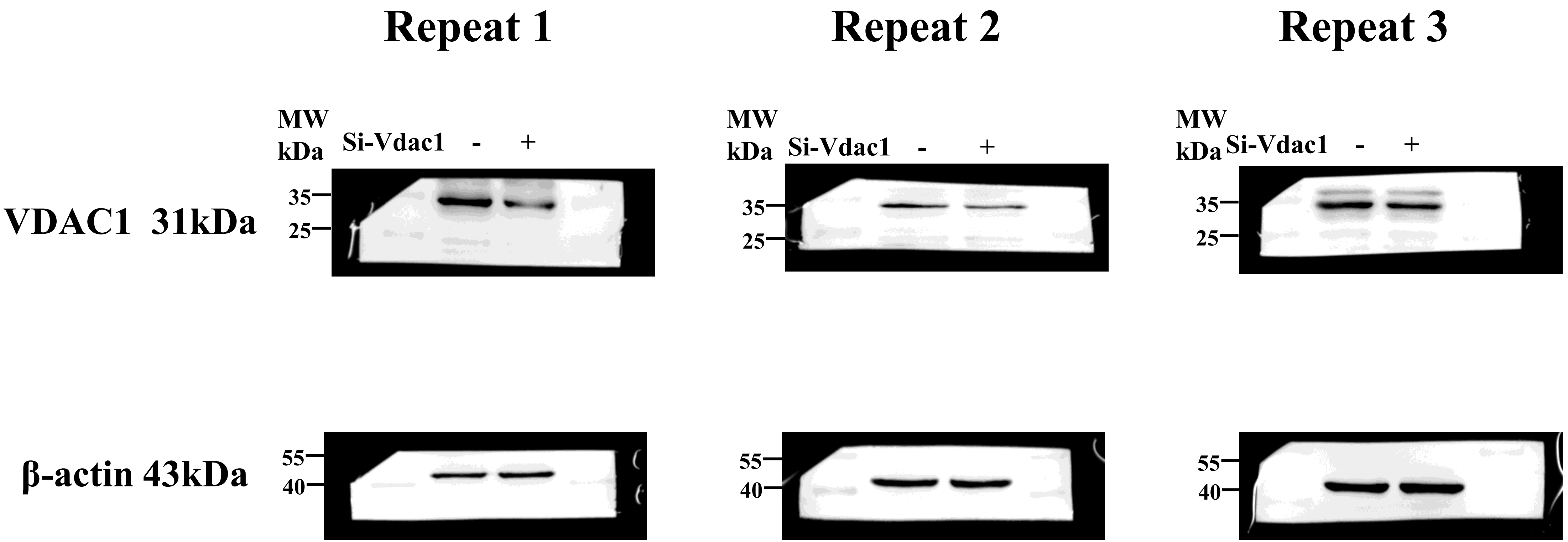

Figure 6H

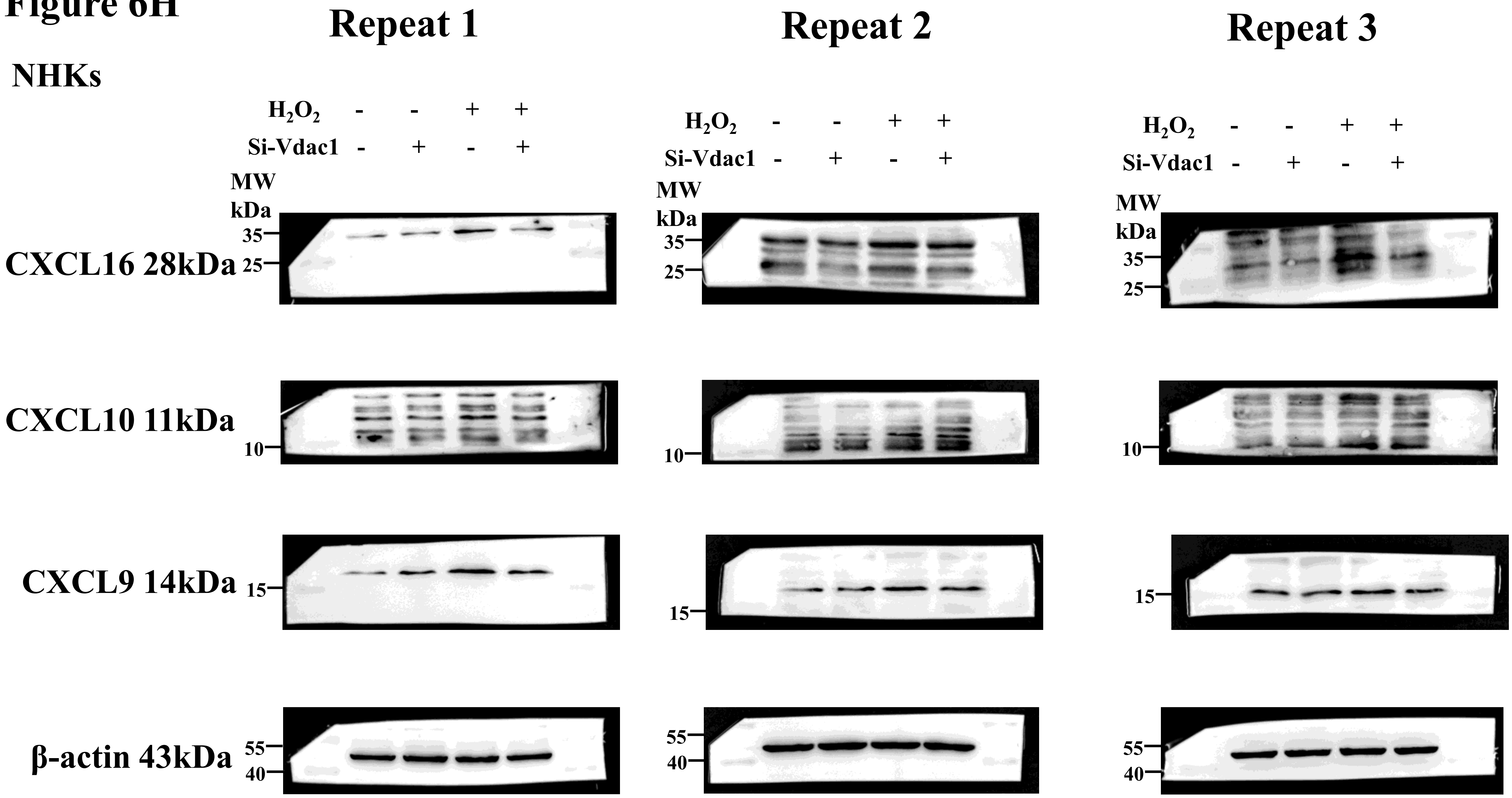

Repeat 1
Repeat 2
Repeat 3
NHKs
H2O2 - - + +
Si-Vdac1 - + - +
MW kDa
35
25
10
15
55
40
CXCL16 28kDa
CXCL10 11kDa
CXCL9 14kDa
β-actin 43kDa

Figure 7E

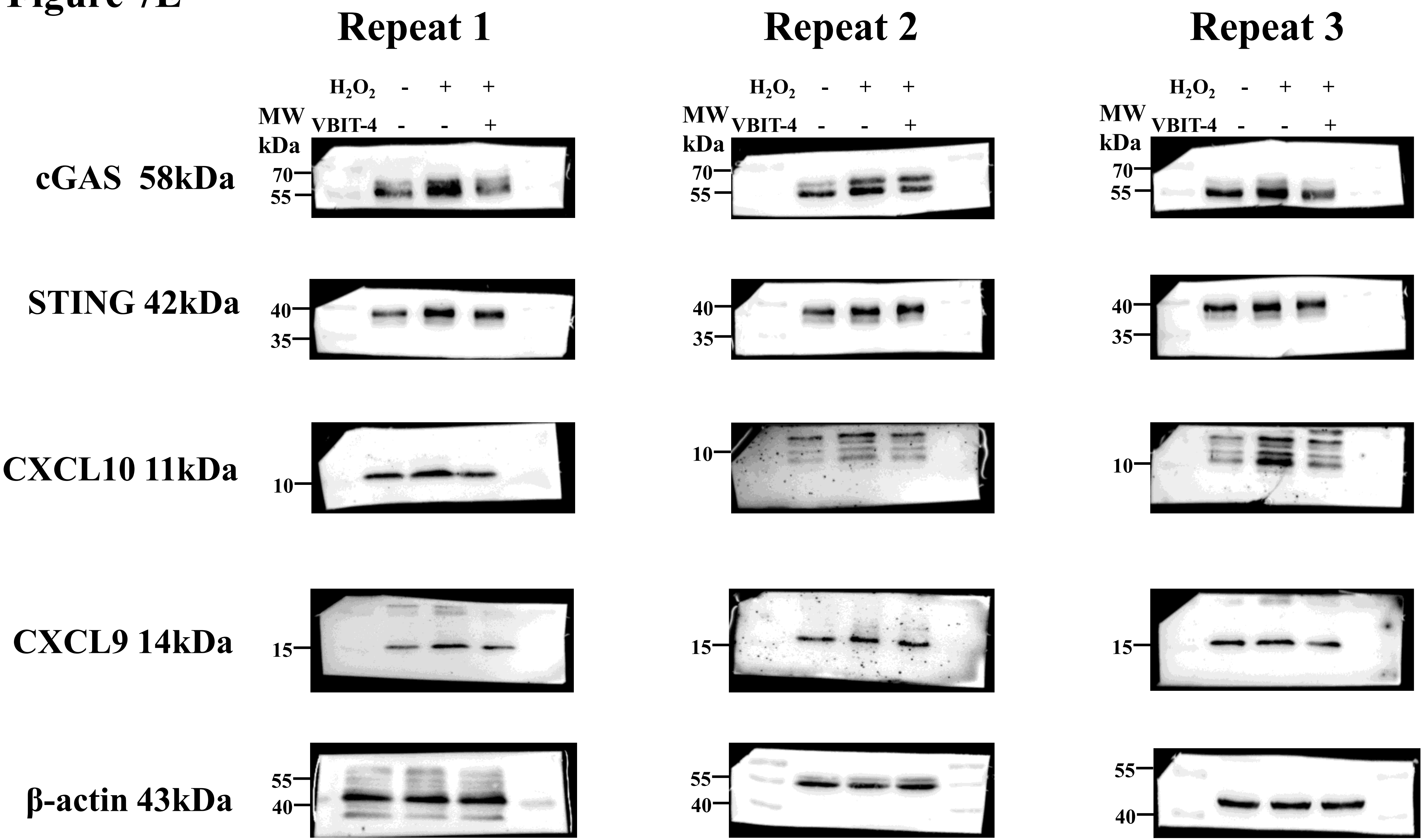
Repeat 1
Repeat 2
Repeat 3
H_2O_2 - + +
VBIT-4 - - +
MW kDa
cGAS 58kDa
STING 42kDa
CXCL10 11kDa
CXCL9 14kDa
β-actin 43kDa
70
55
40
35
10
15
55
40
