# Supplementary material for: Mitochondrial DNA release via VDAC1 in keratinocytes: a key driver of innate immunity and vitiligo pathogenesis
Source: Cell Death Dis. 2026 Mar 18;17(1):318. doi: 10.1038/s41419-026-08585-5 (PMC13039960; doi:10.1038/s41419-026-08585-5)
Supplement: Supplementary file 4 — Supplementary Data-Cell images [file 41419_2026_8585_MOESM4_ESM.pdf]

Figure 1B Repeat 1

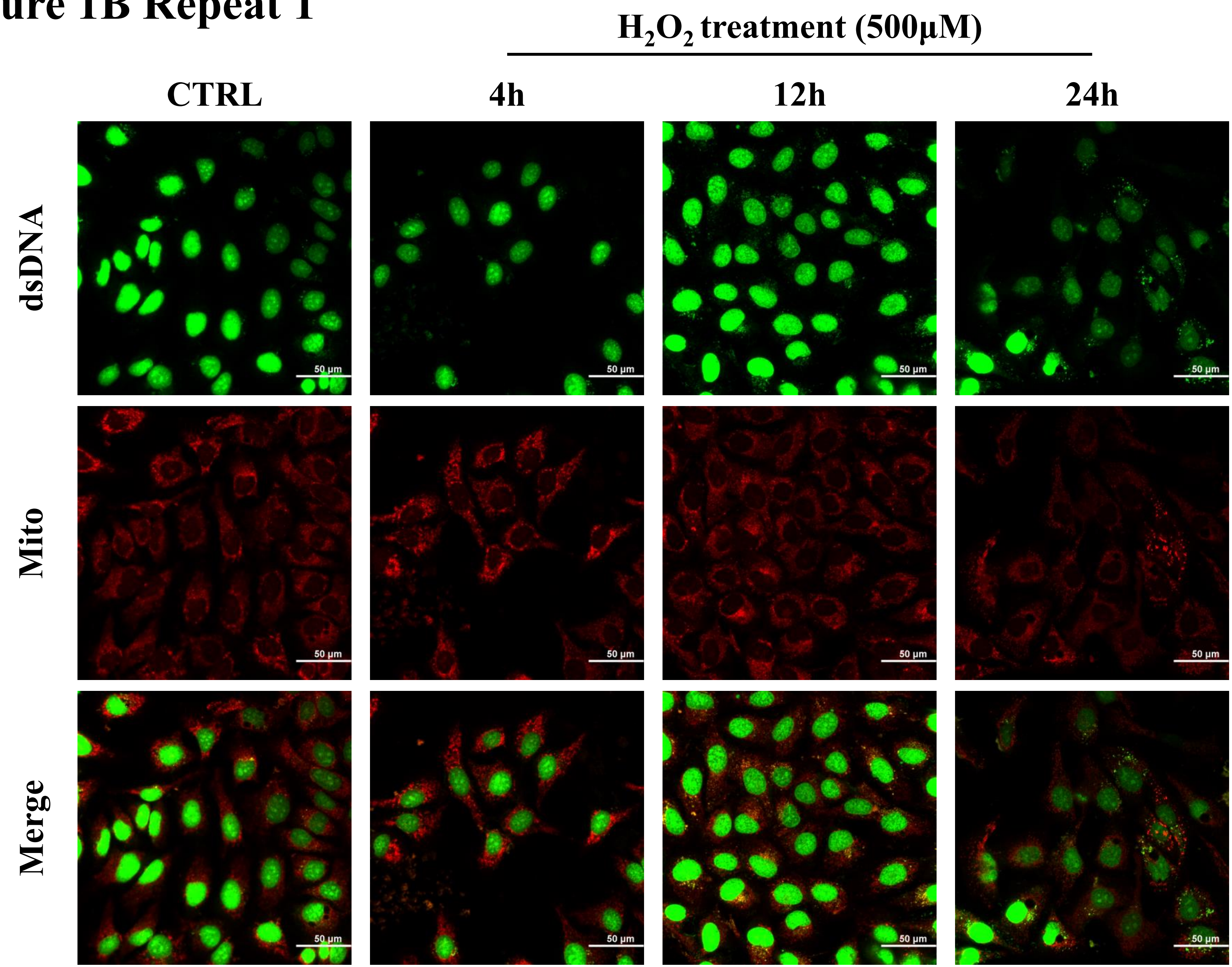

Figure 1B Repeat 2

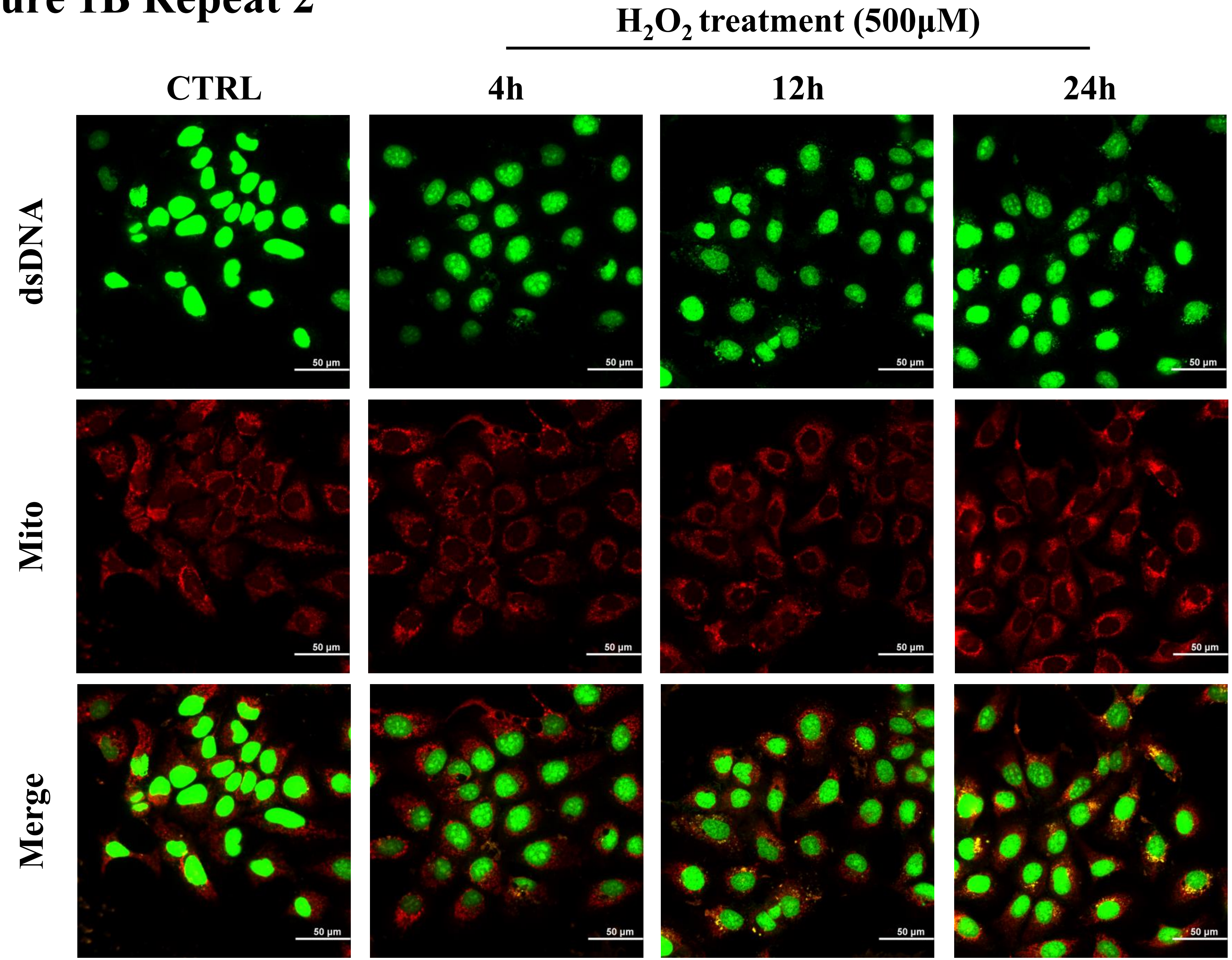

Figure 1B Repeat 3

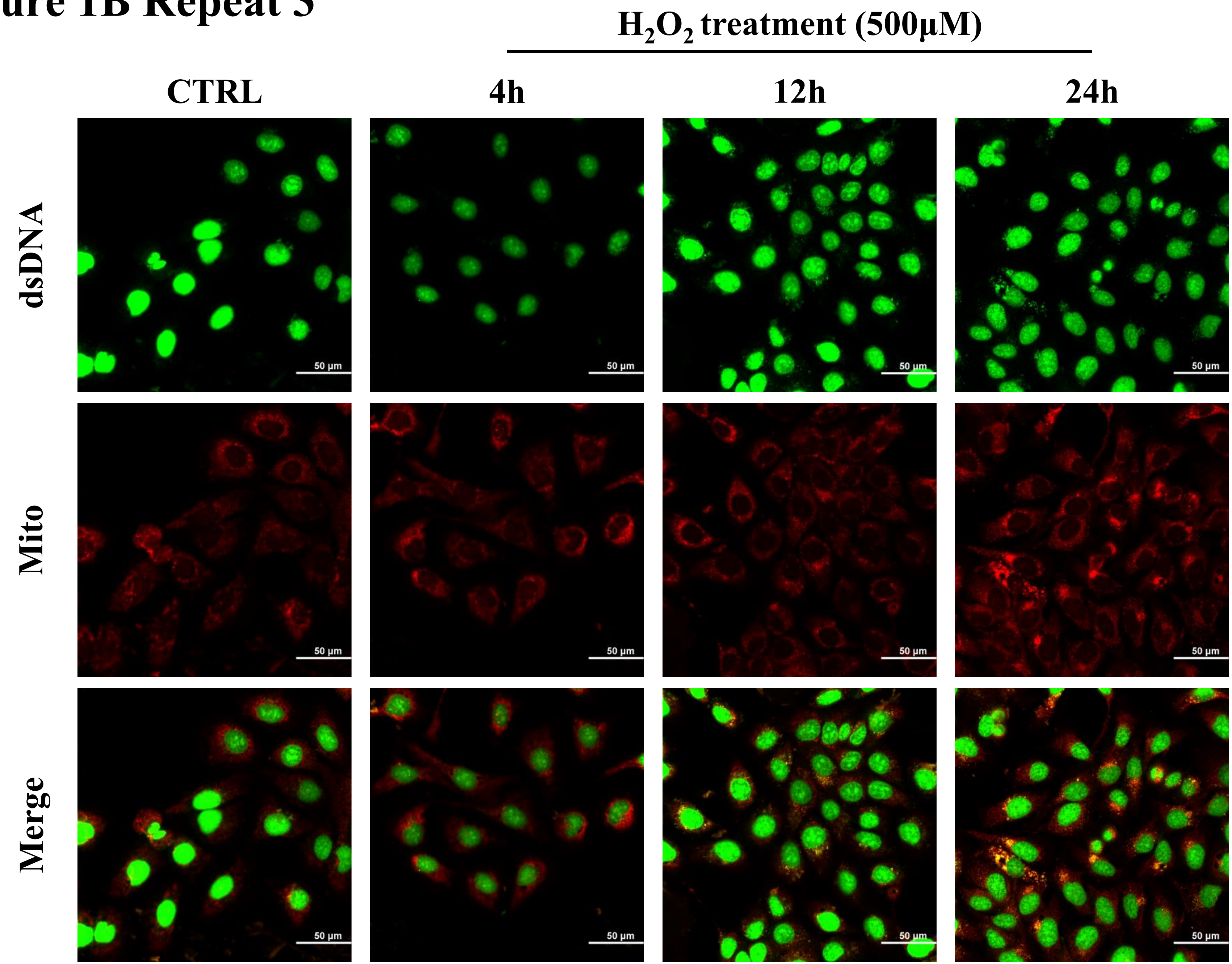

Figure 1I

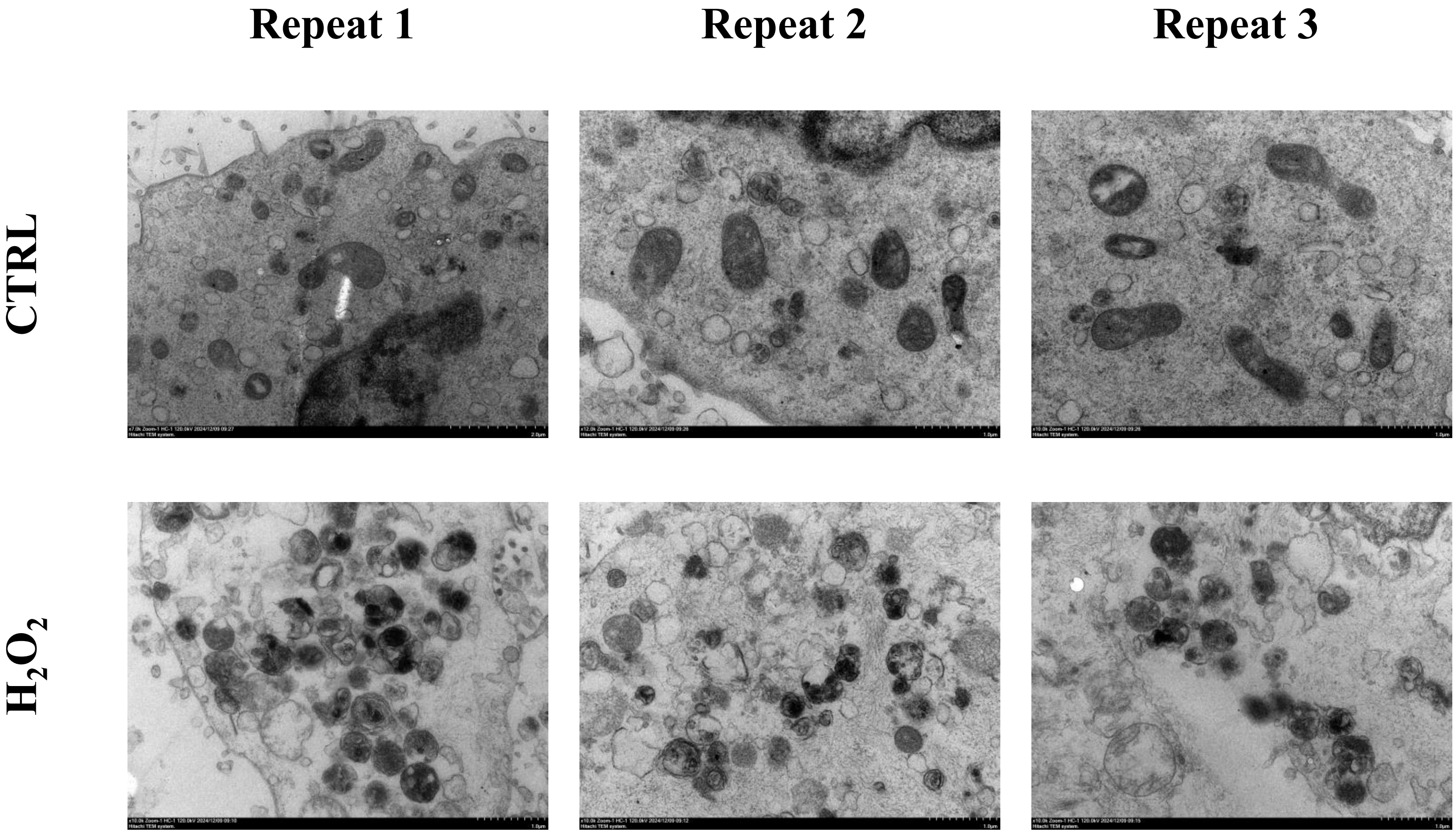

Figure 3D Repeat 1

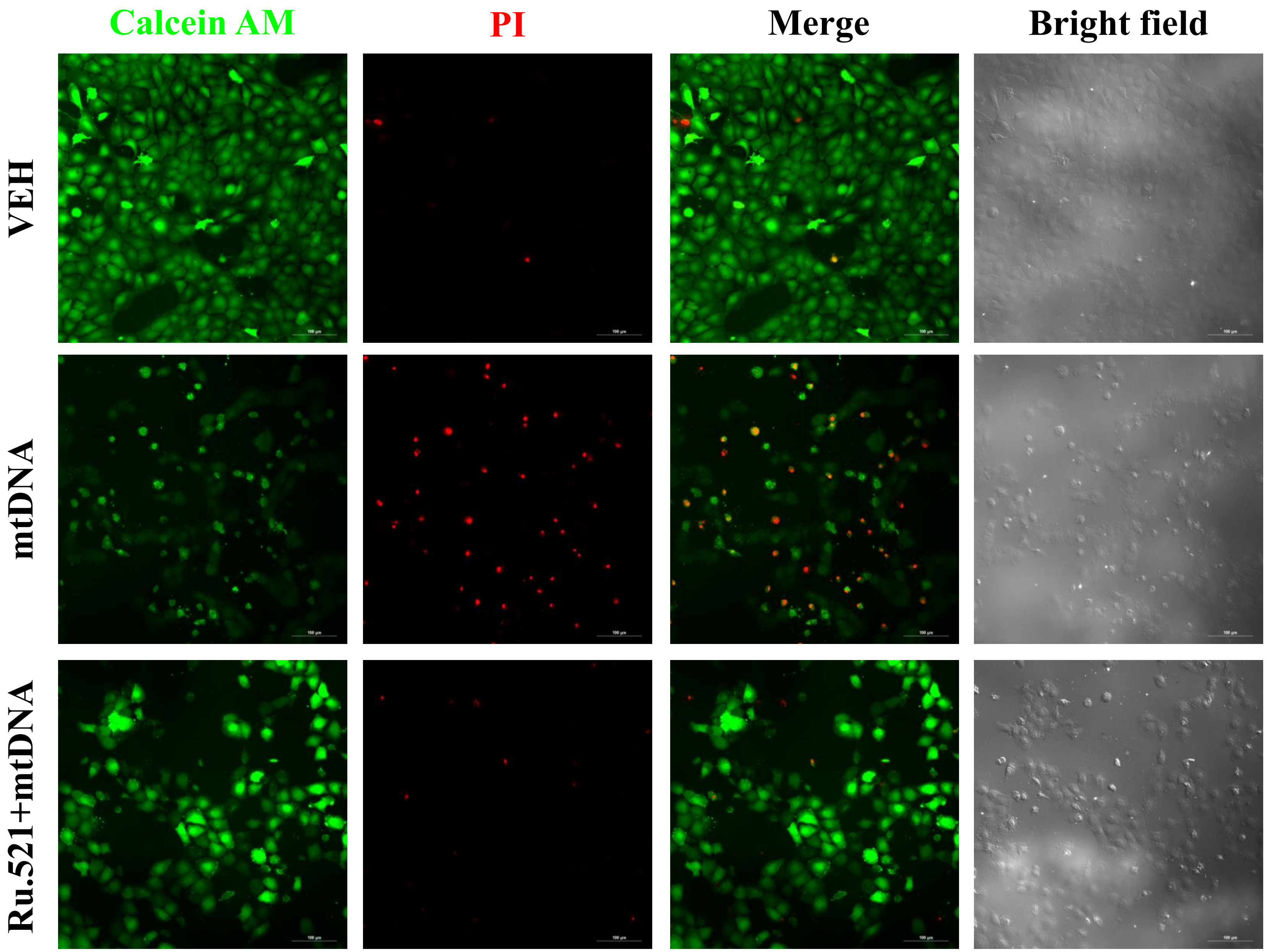

Figure 3D Repeat 2

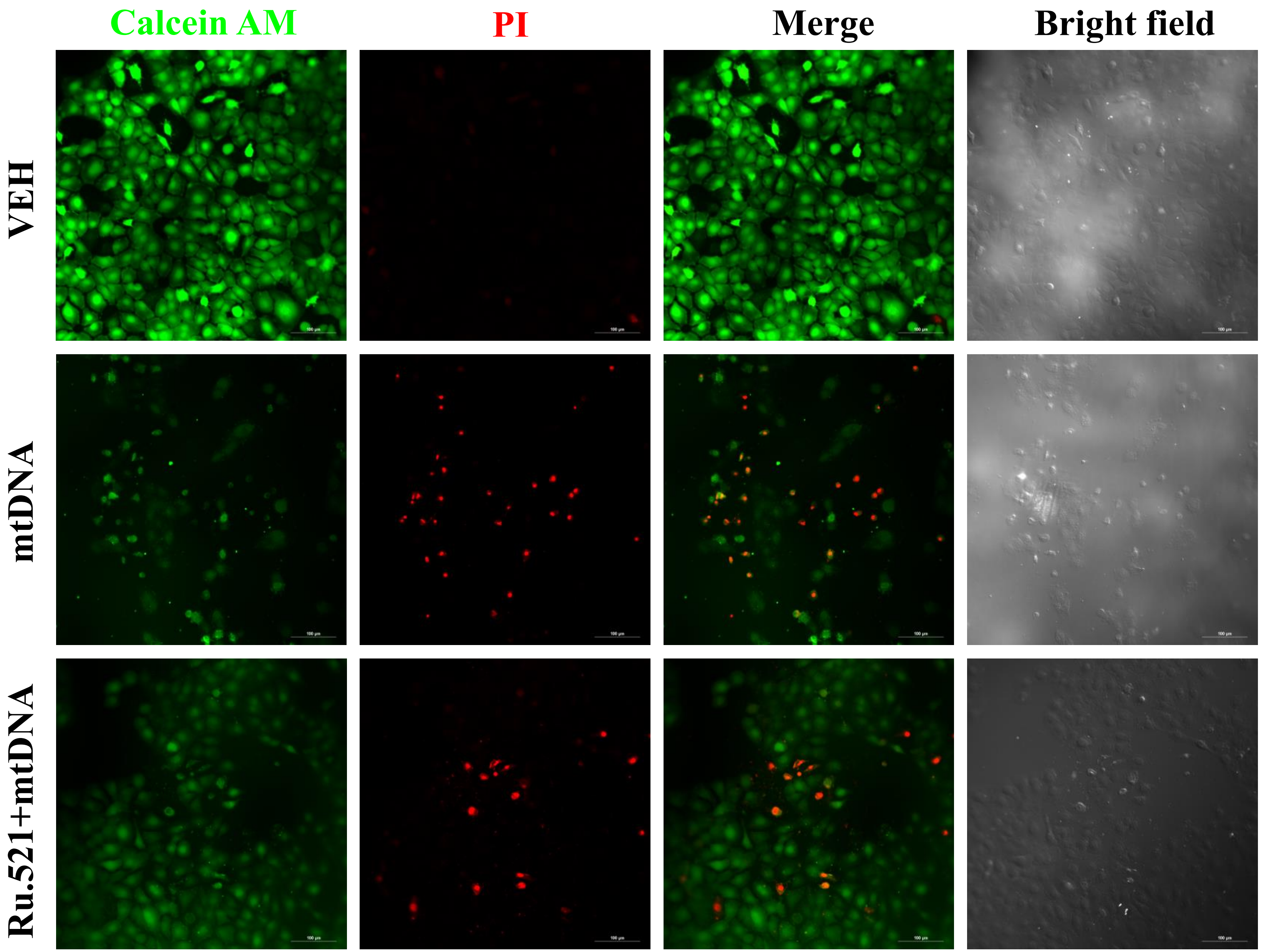

Figure 3D Repeat 3

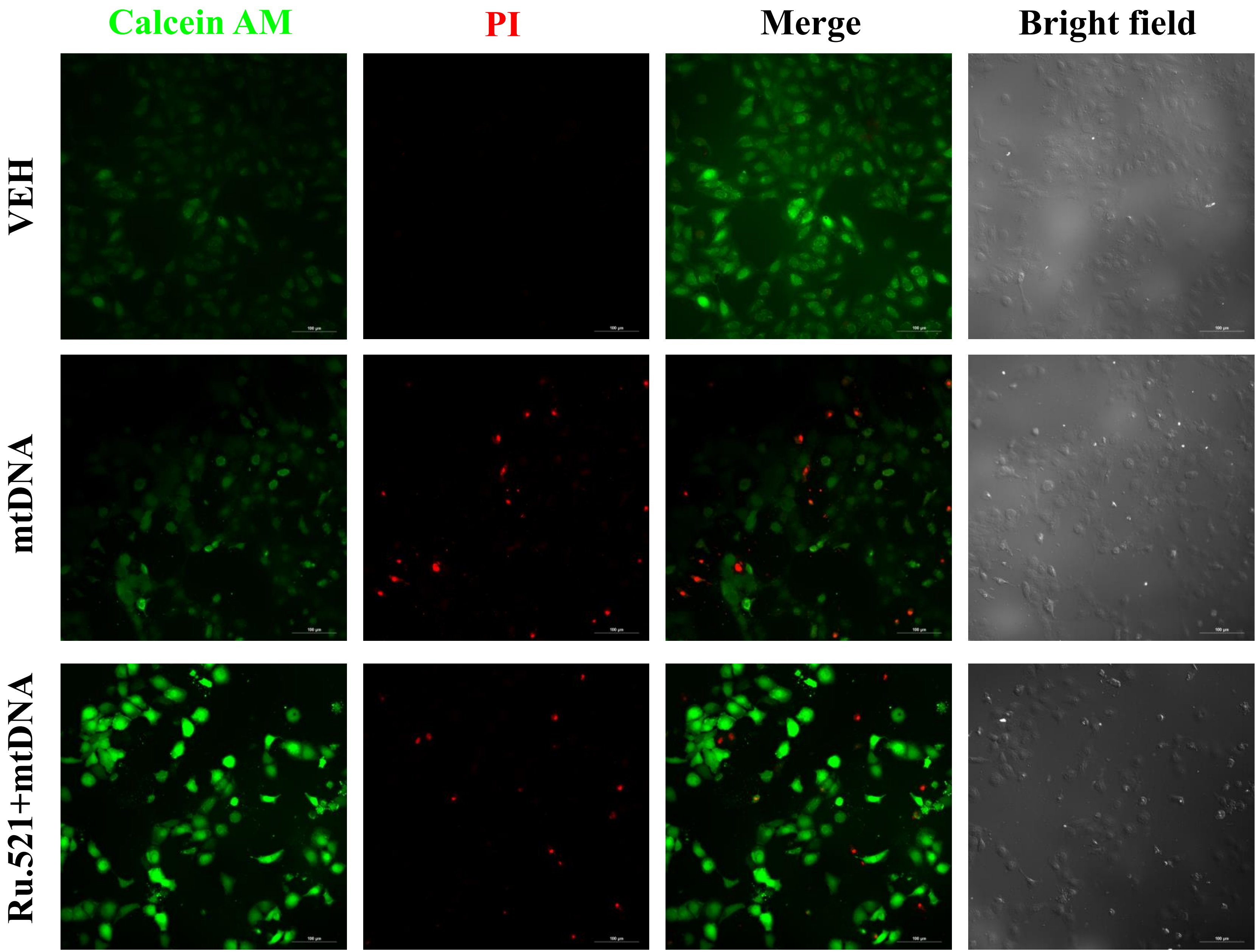

Figure 3E

**Repeat 1**		**VEH**	**mtDNA**	**Ru.521+mtDNA**
	PI	3	30	5
	Total	238	62	99
	%	1.26	48.39	5.05
**Repeat 2**		**VEH**	**mtDNA**	**Ru.521+mtDNA**
	PI	3	15	16
	Total	181	58	134
	%	1.66	25.86	11.94
**Repeat 3**		**VEH**	**mtDNA**	**Ru.521+mtDNA**
	PI	4	26	10
	Total	131	43	94
	%	3.05	60.47	10.64

Figure 3I Repeat 1

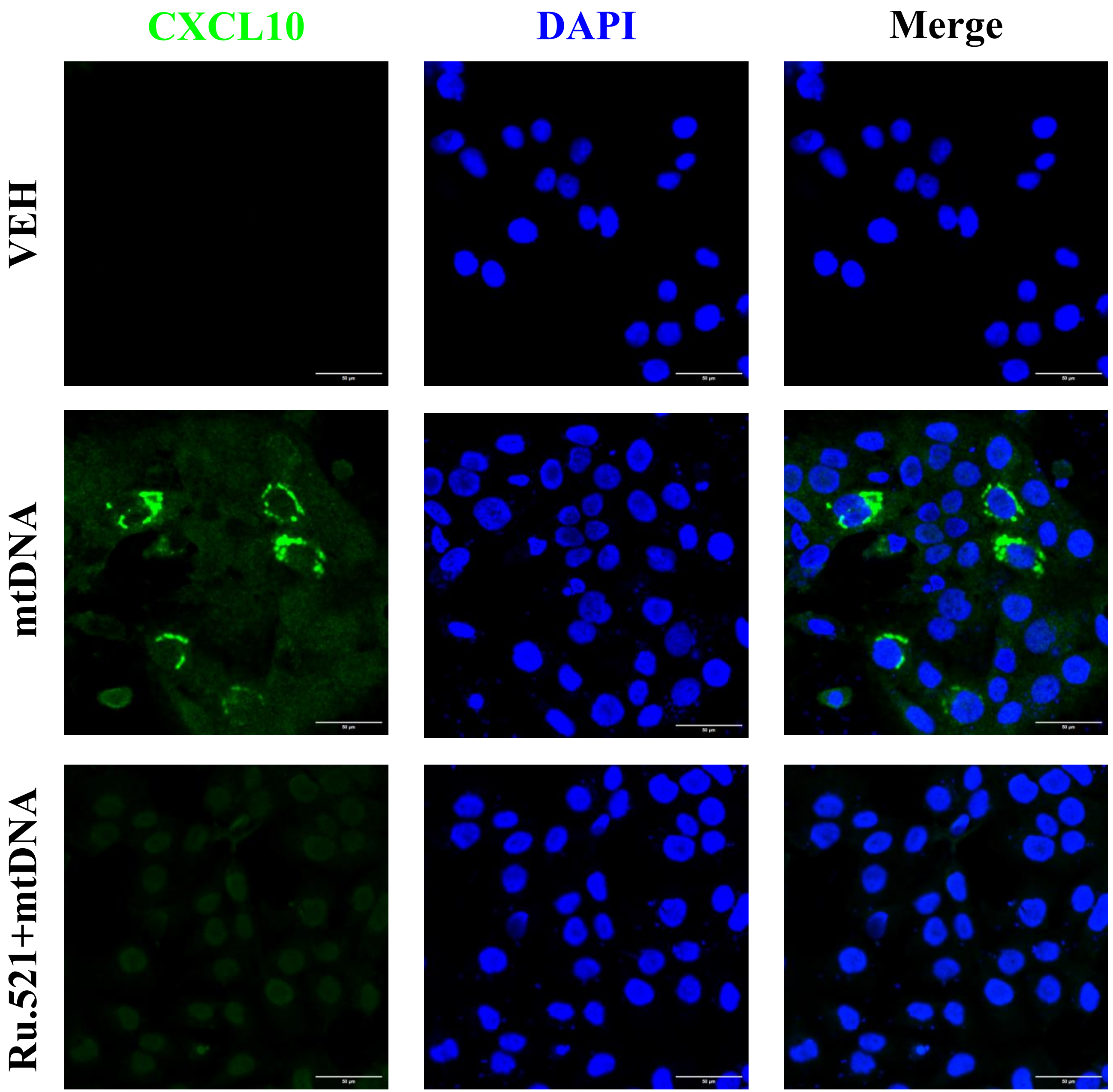

Figure 3I Repeat 2

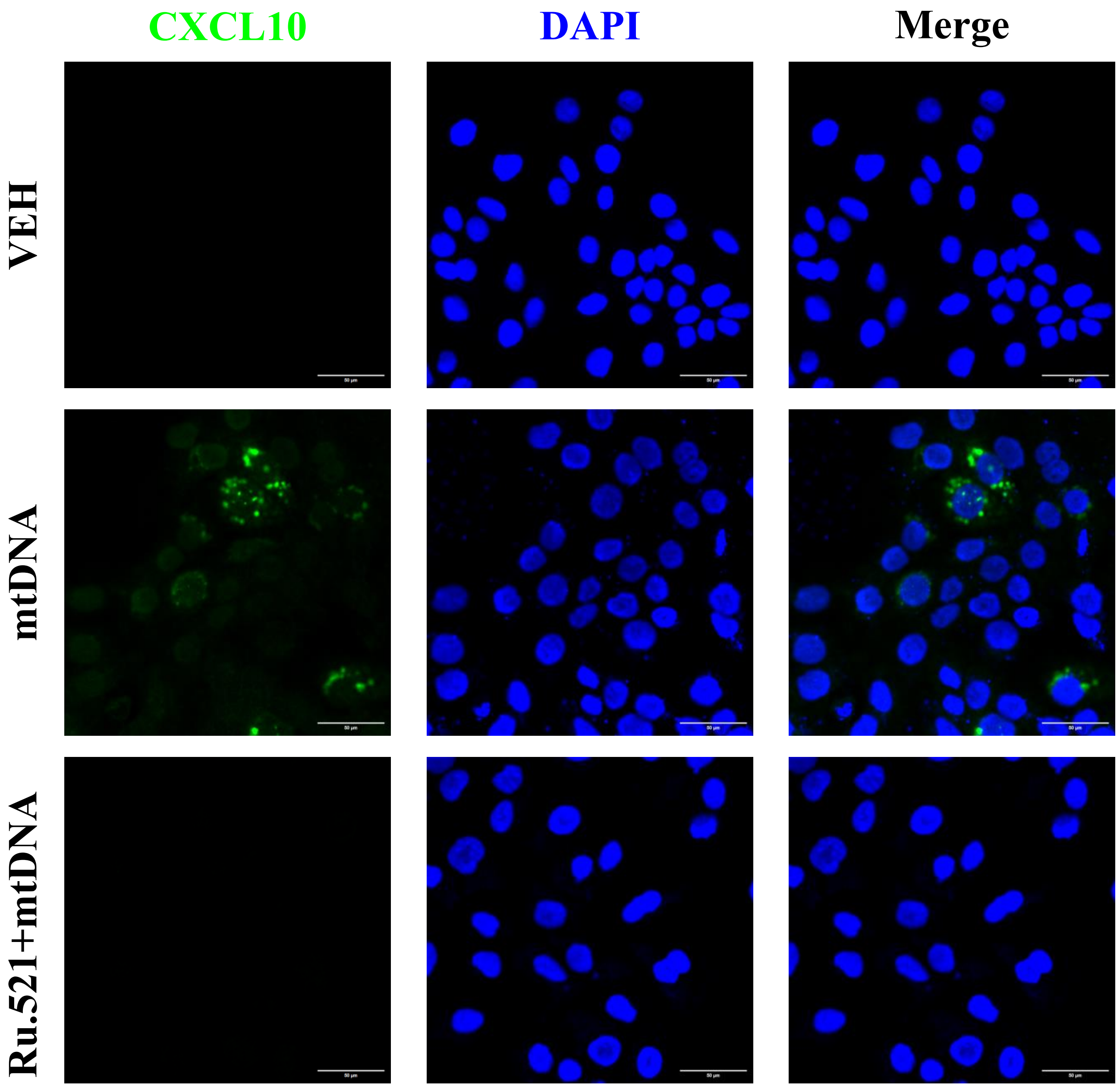

Figure 3I Repeat 3

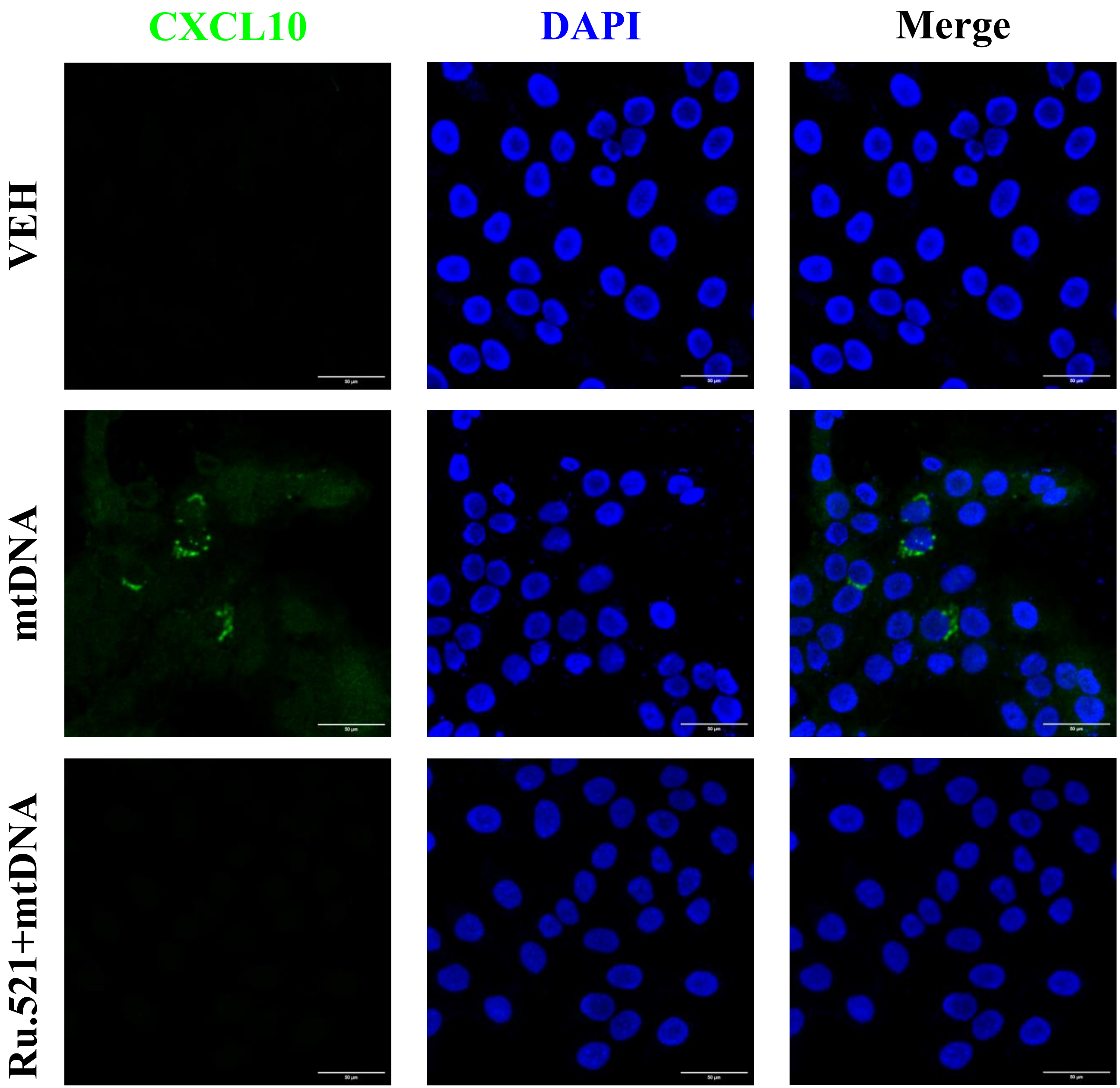

Figure 4E Repeat 1

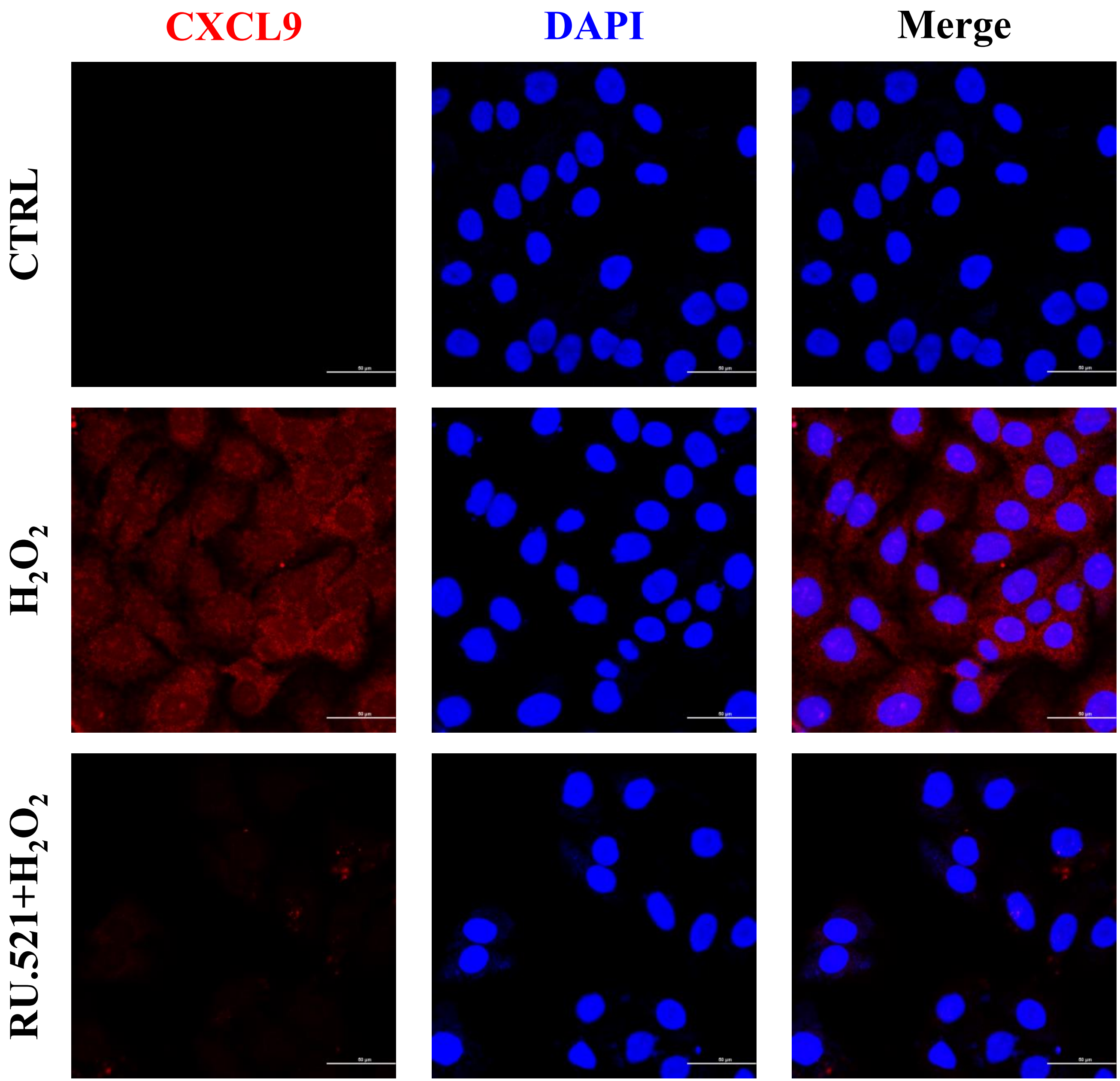

Figure 4E Repeat 2

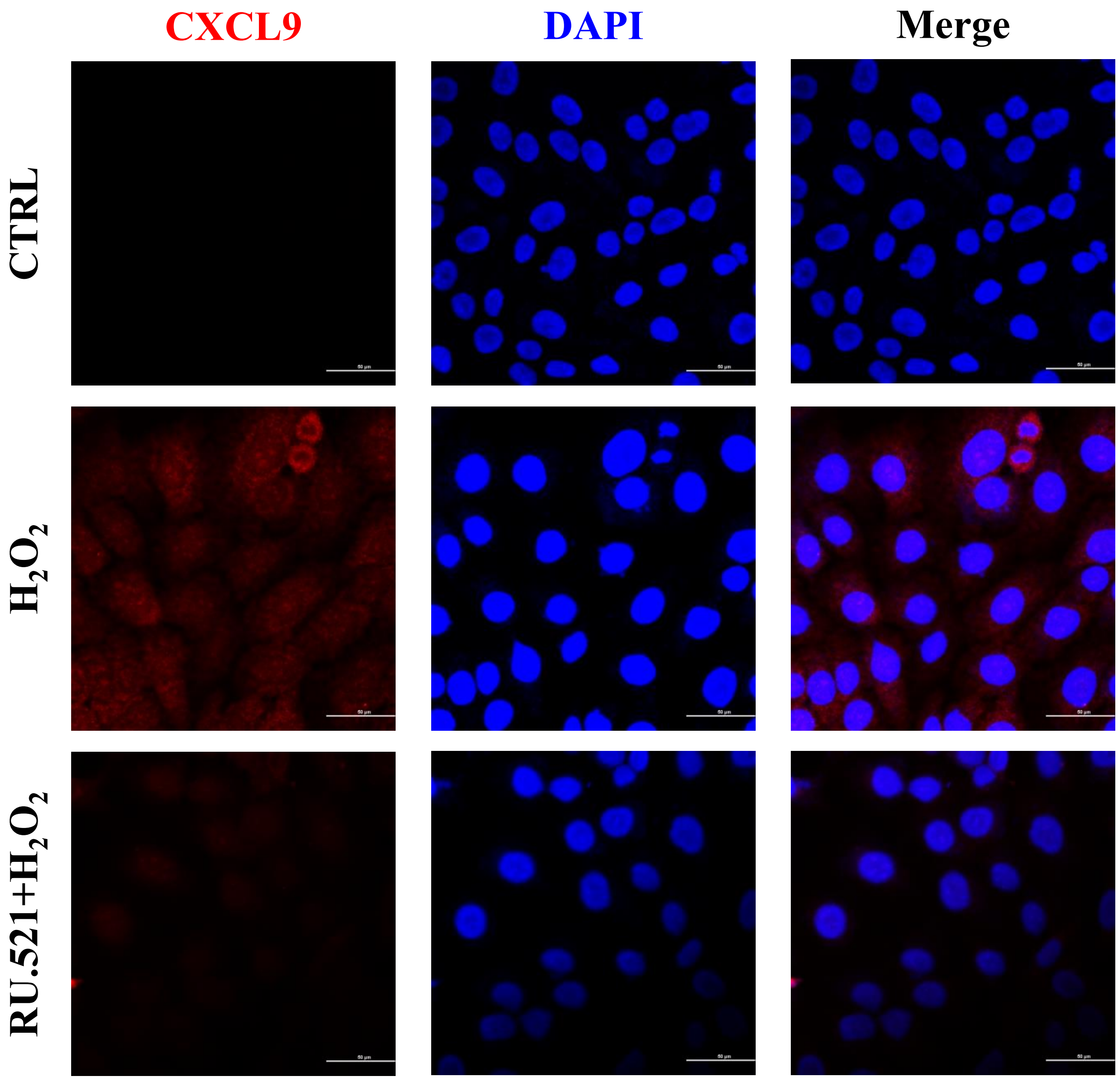

Figure 4E Repeat 3

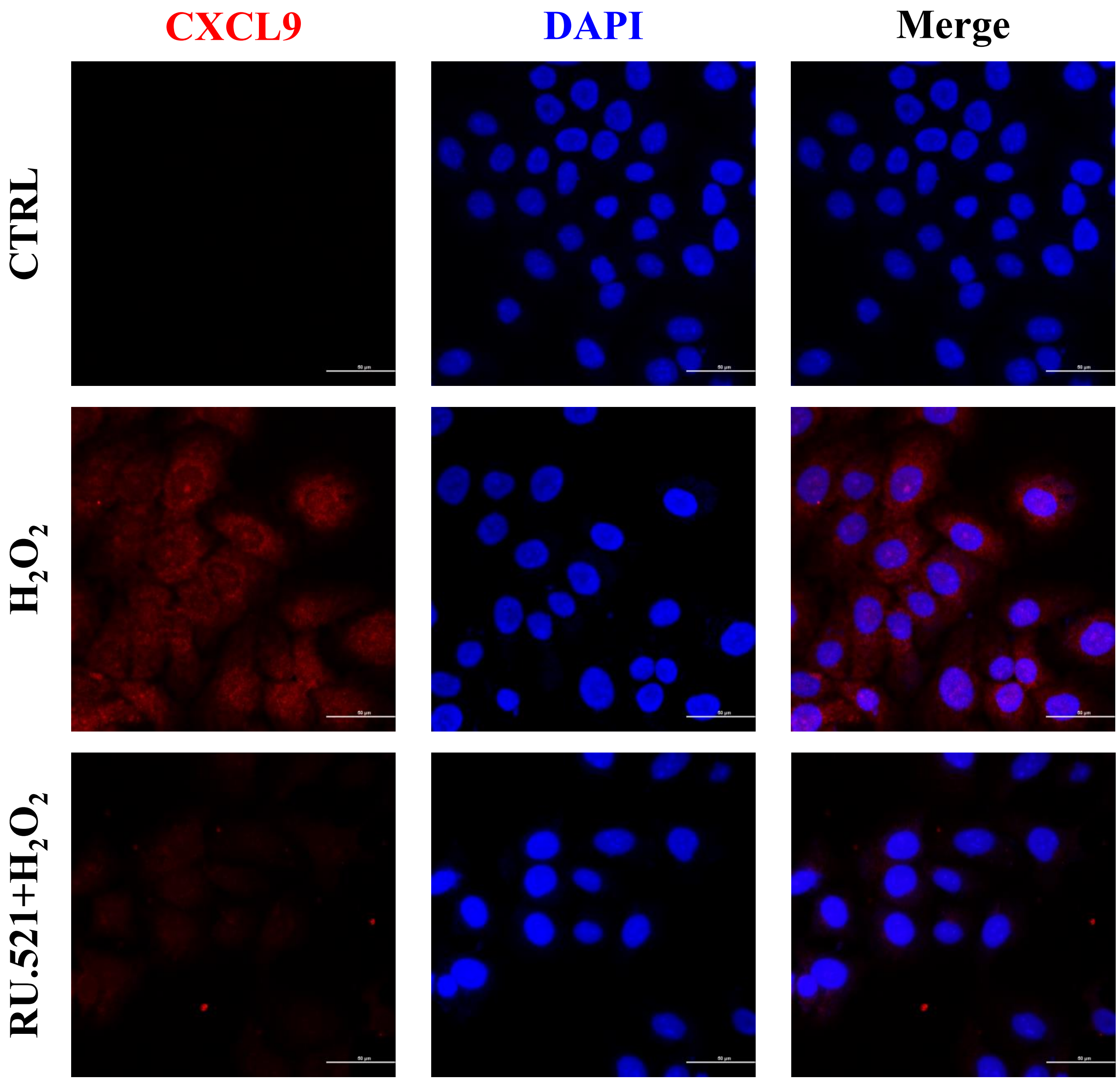

Figure 4F Repeat 1

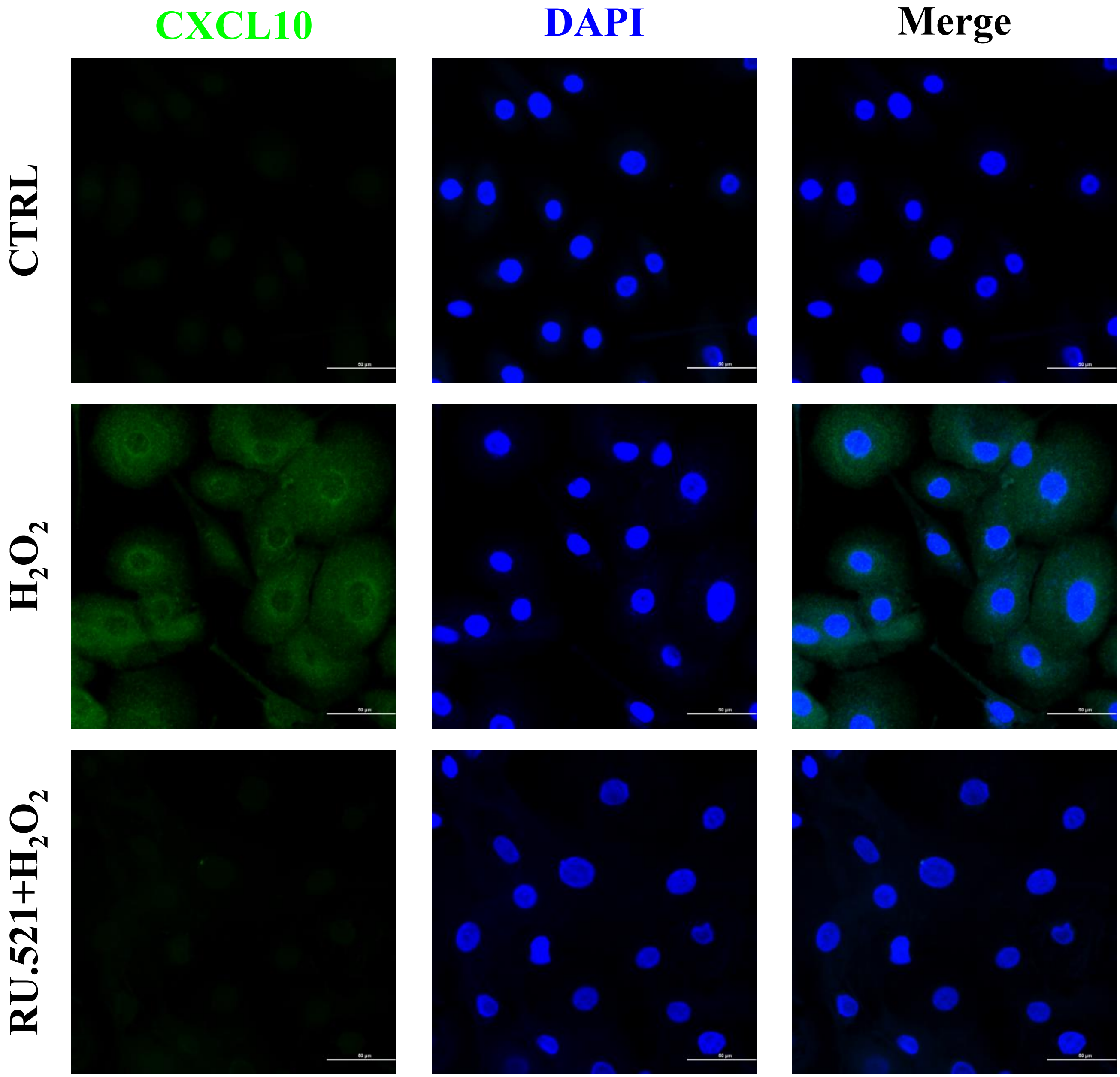

Figure 4F Repeat 2

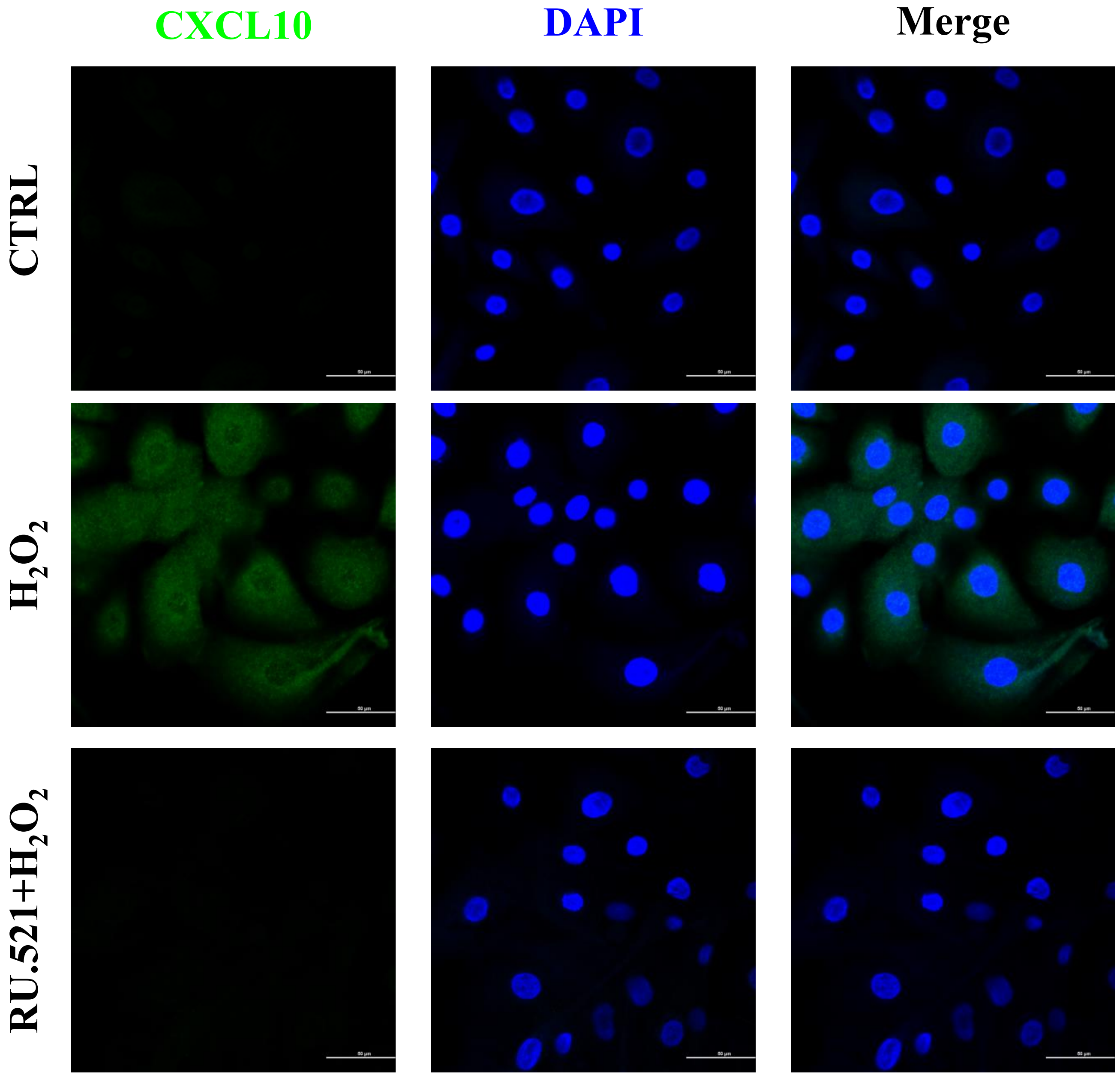

Figure 4F Repeat 3

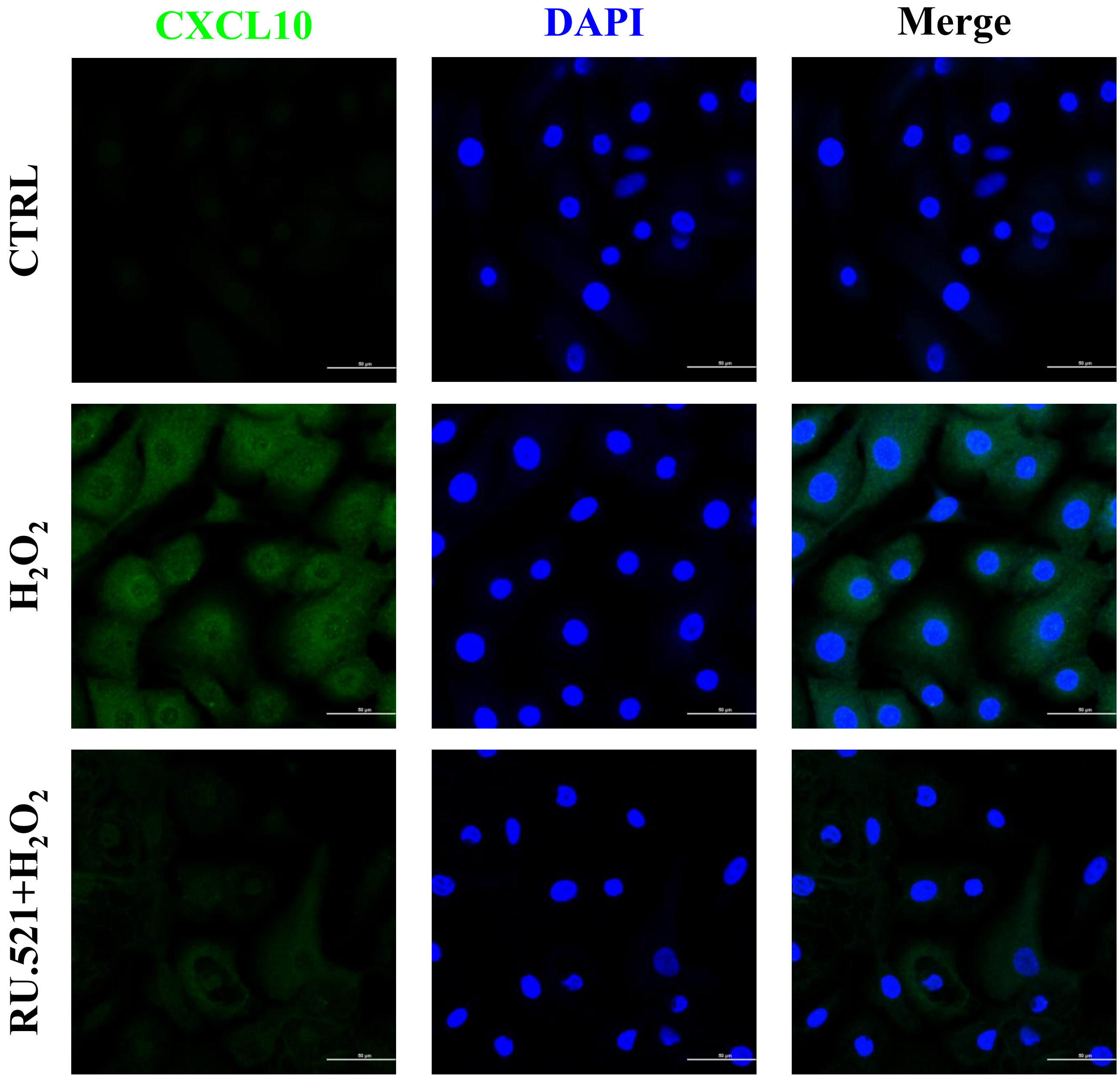

Figure 5A

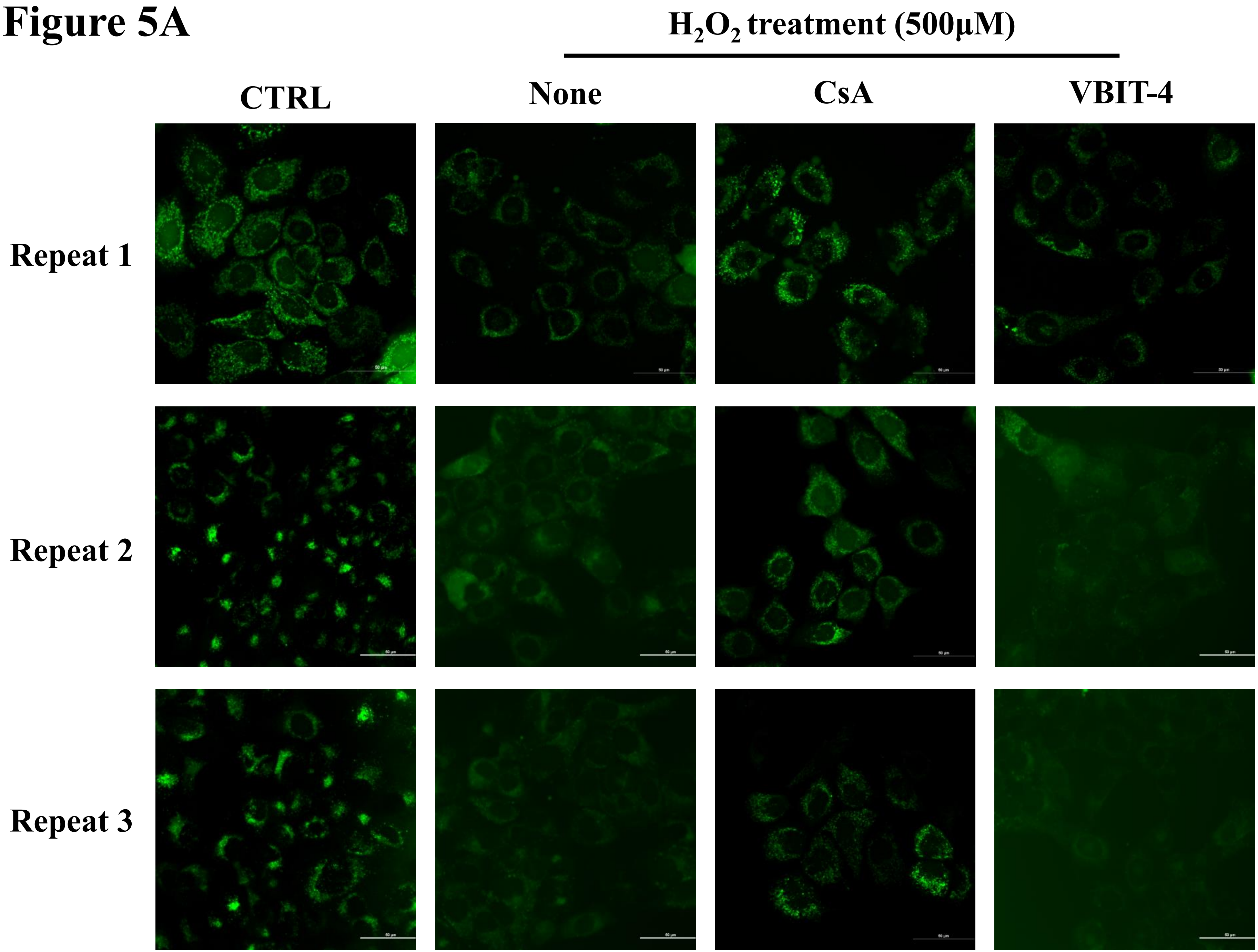

H_2O_2 treatment (500μM)
CTRL
None
CsA
VBIT-4
Repeat 1
Repeat 2
Repeat 3

Figure 5A

Repeat 1		CTRL	H_2O_2	$CsA+H_2O_2$	$VBIT\text{-}4+H_2O_2$
	Mean	96.025	42.127	66.879	47.277
	%	100.00	43.87	69.65	49.23
**Repeat 2**		**CTRL**	**H_2O_2**	**$CsA+H_2O_2$**	**$VBIT\text{-}4+H_2O_2$**
	Mean	97.564	40.183	77.32	34.964
	%	100.00	41.19	79.25	35.84
**Repeat 3**		**CTRL**	**H_2O_2**	**$CsA+H_2O_2$**	**$VBIT\text{-}4+H_2O_2$**
	Mean	98.674	31.615	59.686	36.529
	%	100.00	32.04	60.49	37.02

Figure 5F Repeat 1

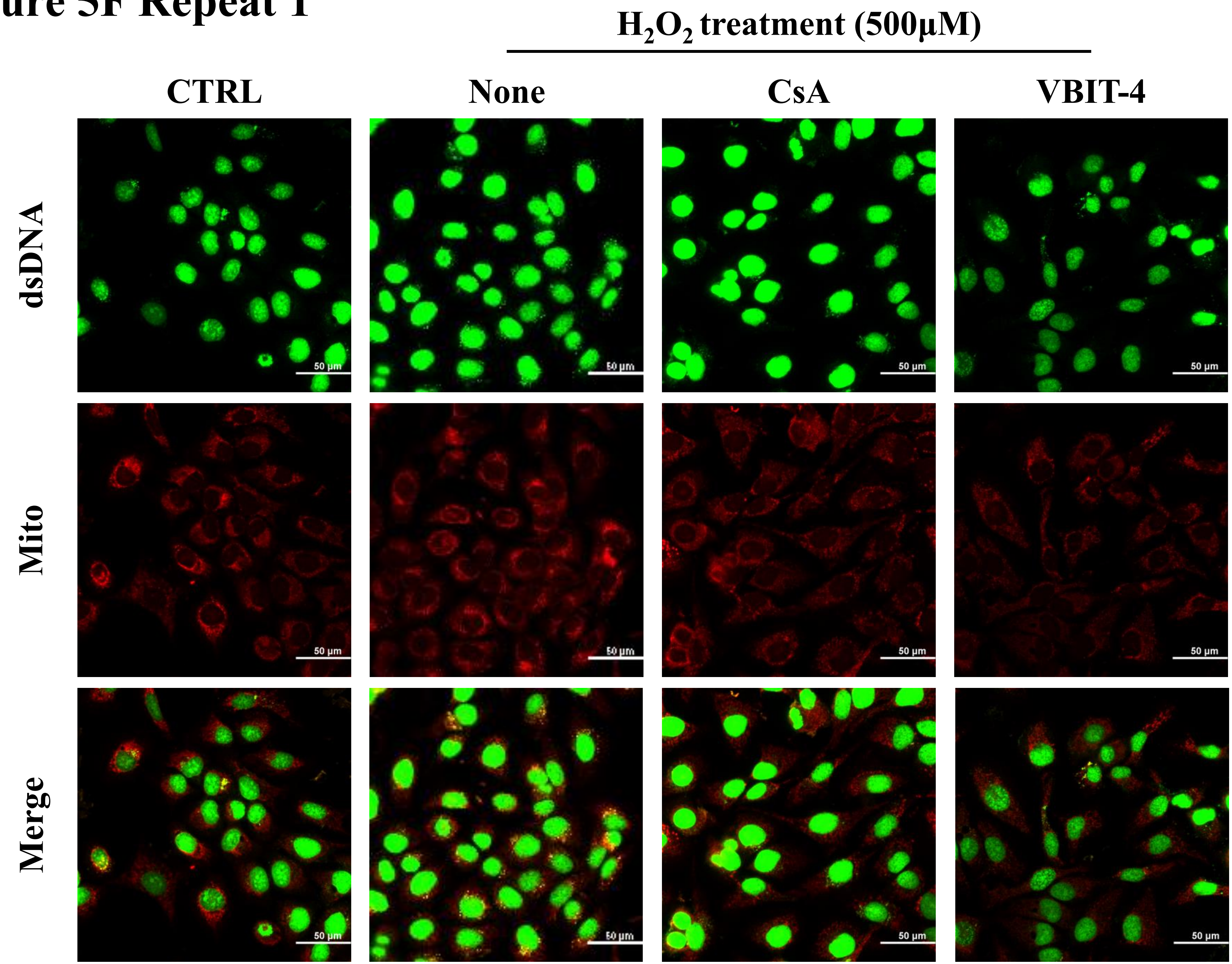

Figure 5F Repeat 2

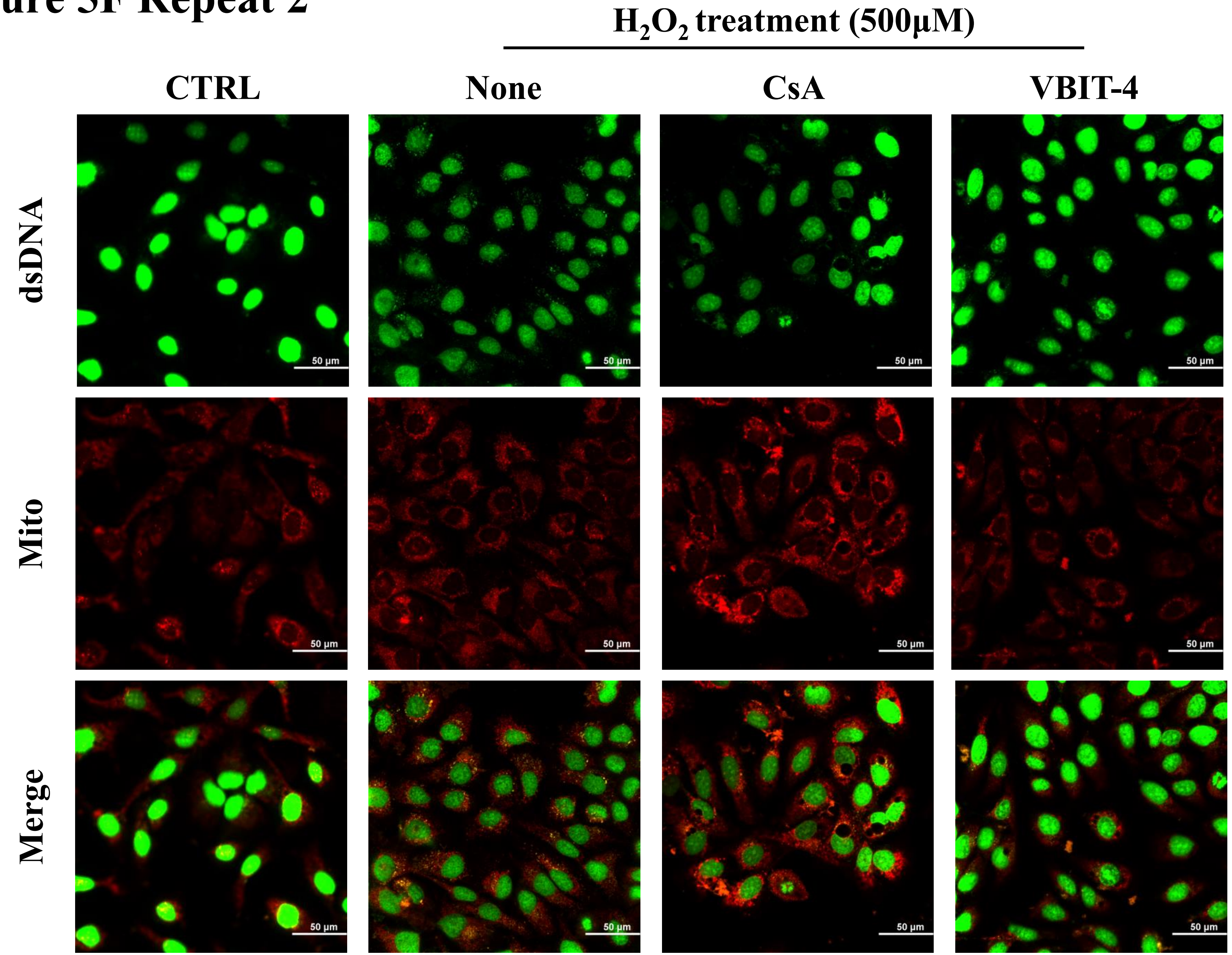

Figure 5F Repeat 3

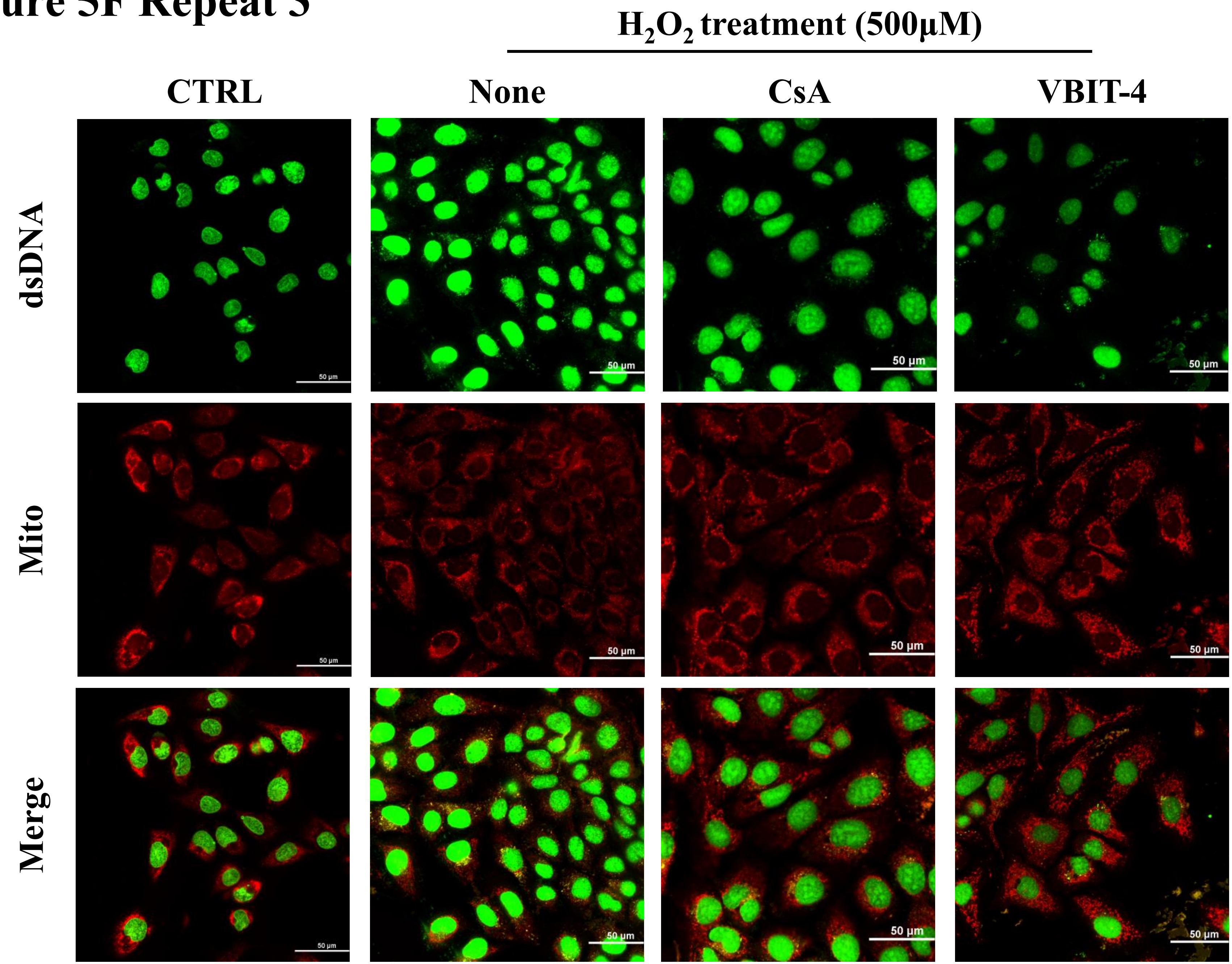

Figure 7B Repeat 1

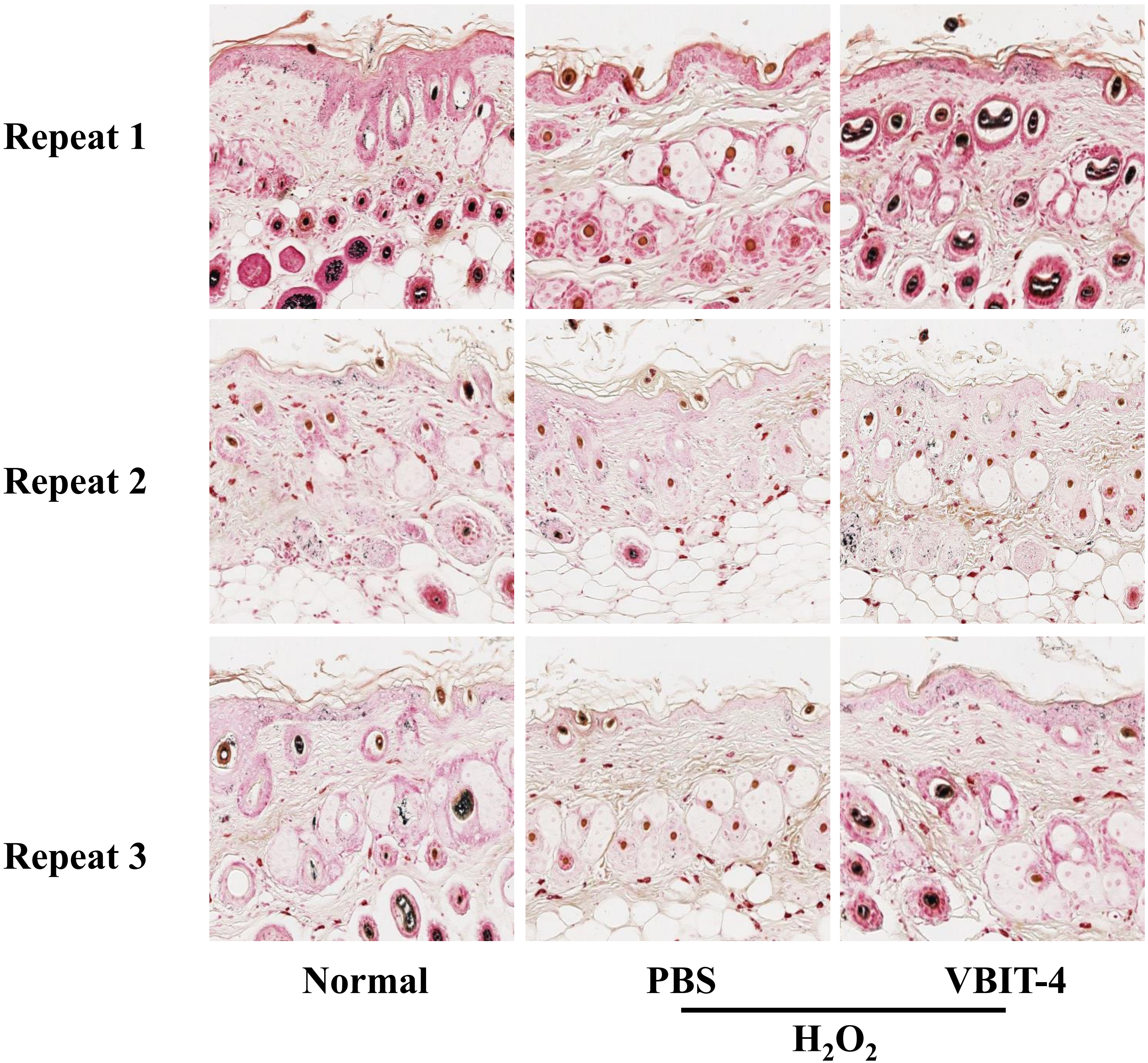

Figure 7C Repeat 1

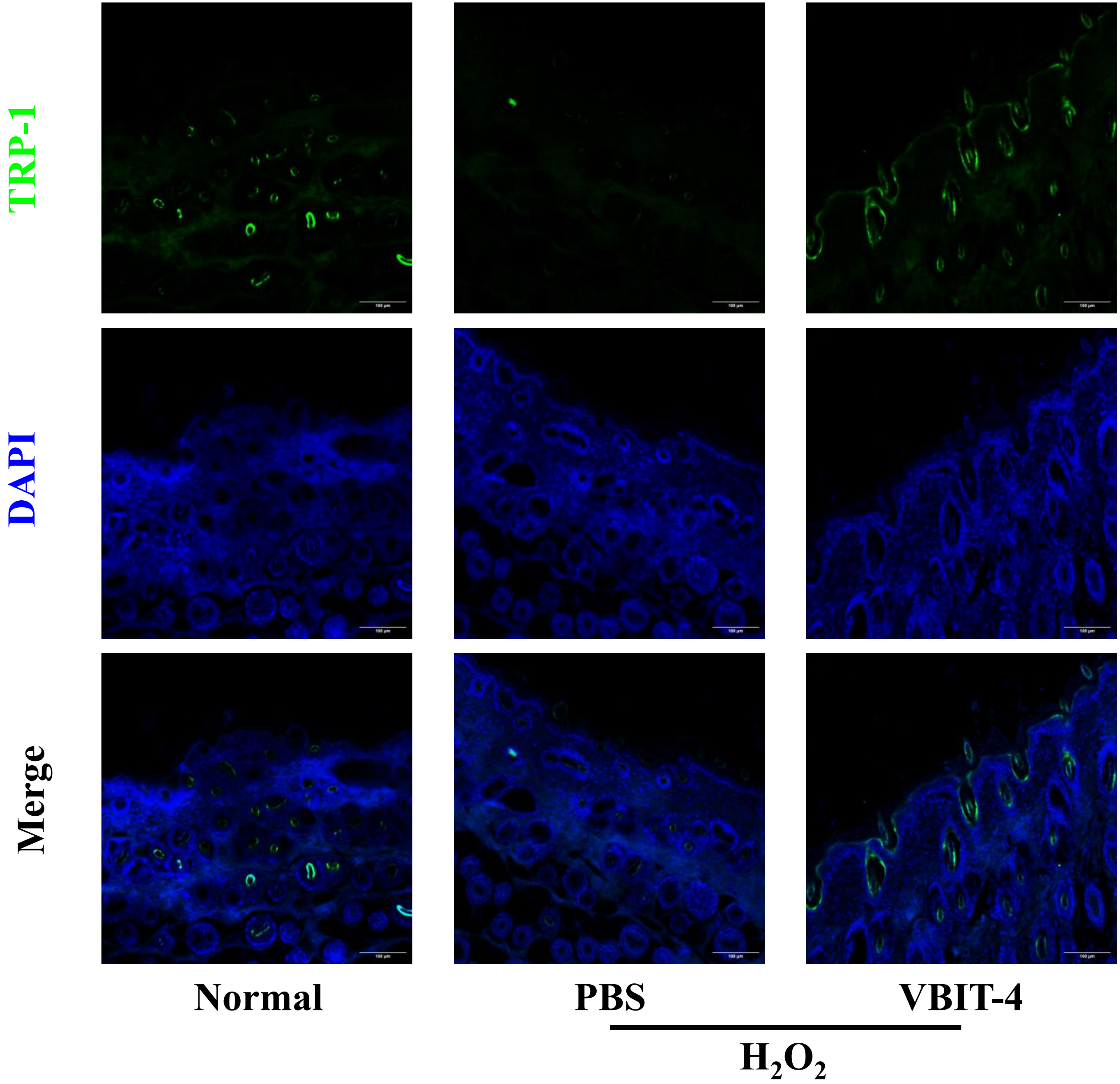

Figure 7C Repeat 2

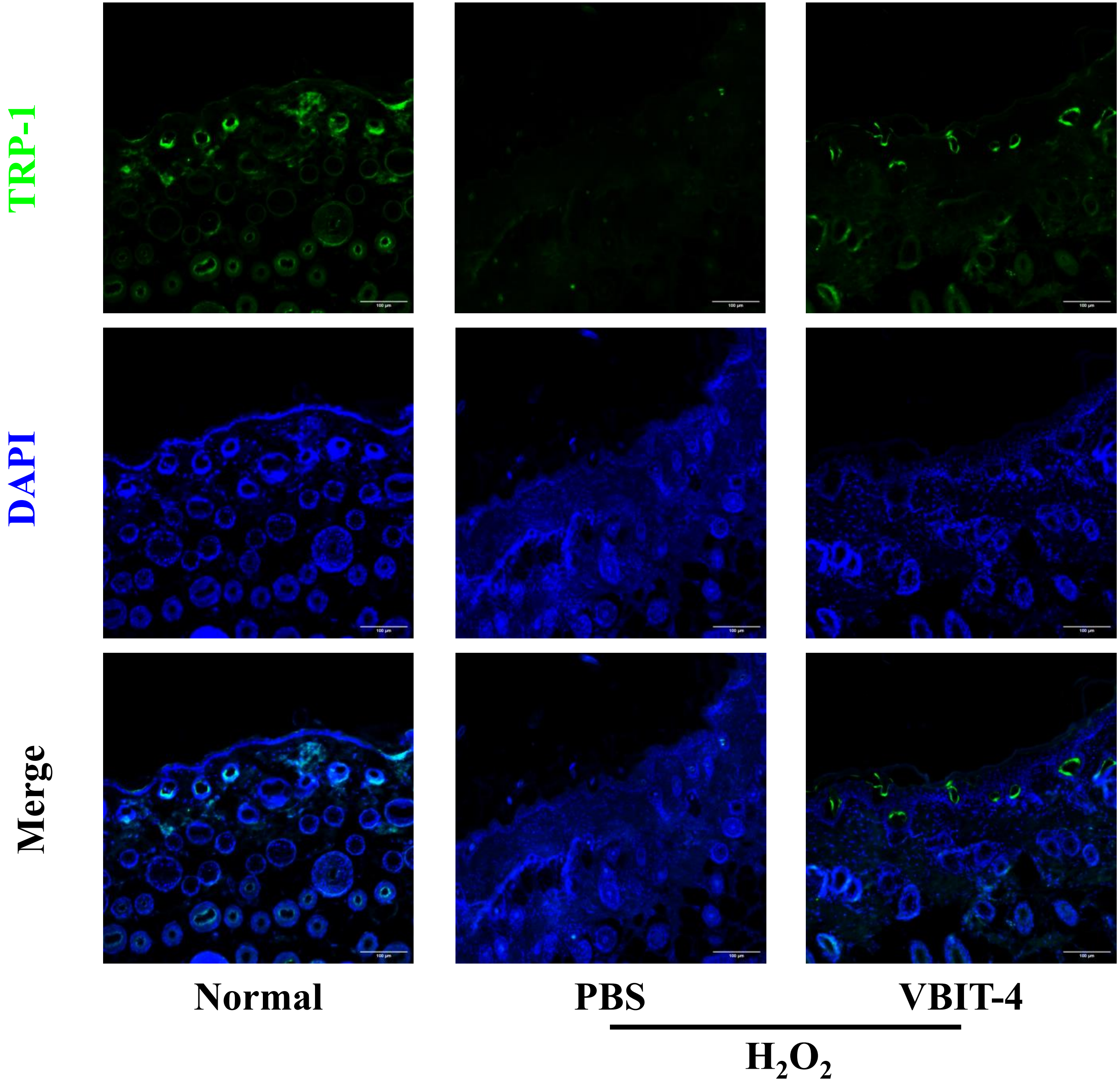

Figure 7C Repeat 3

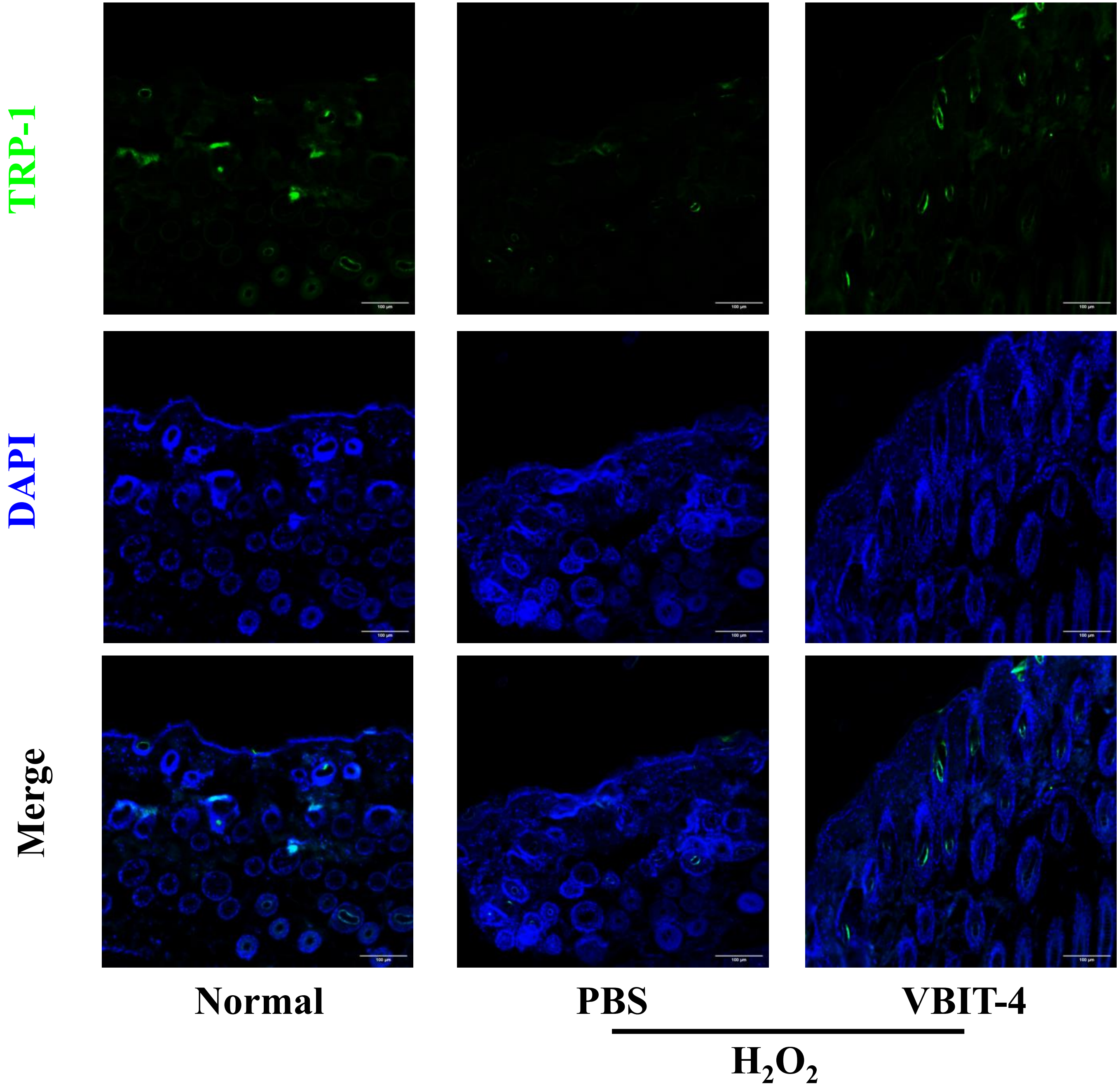

Figure 7D Repeat 1

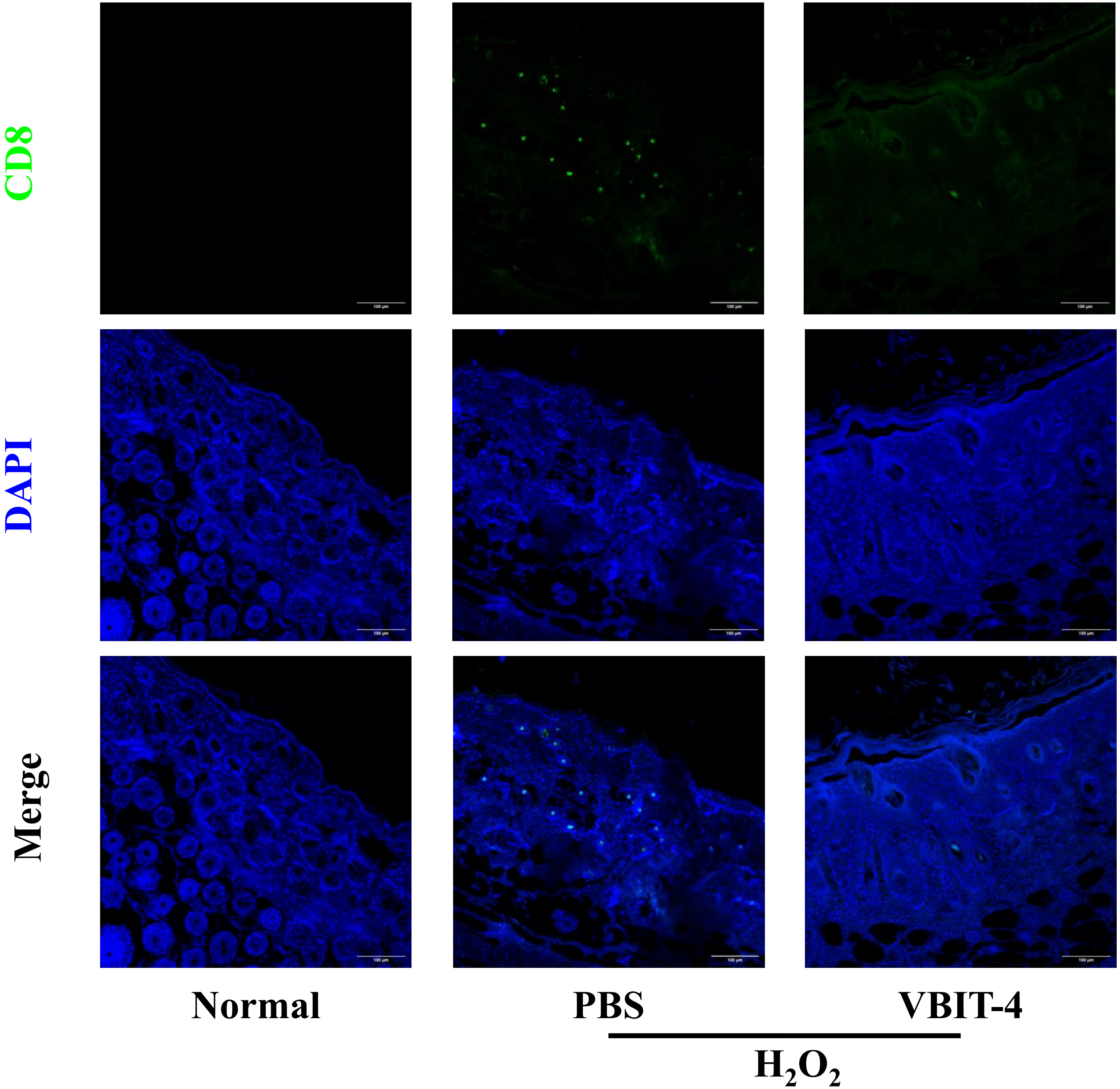

Figure 7D Repeat 2

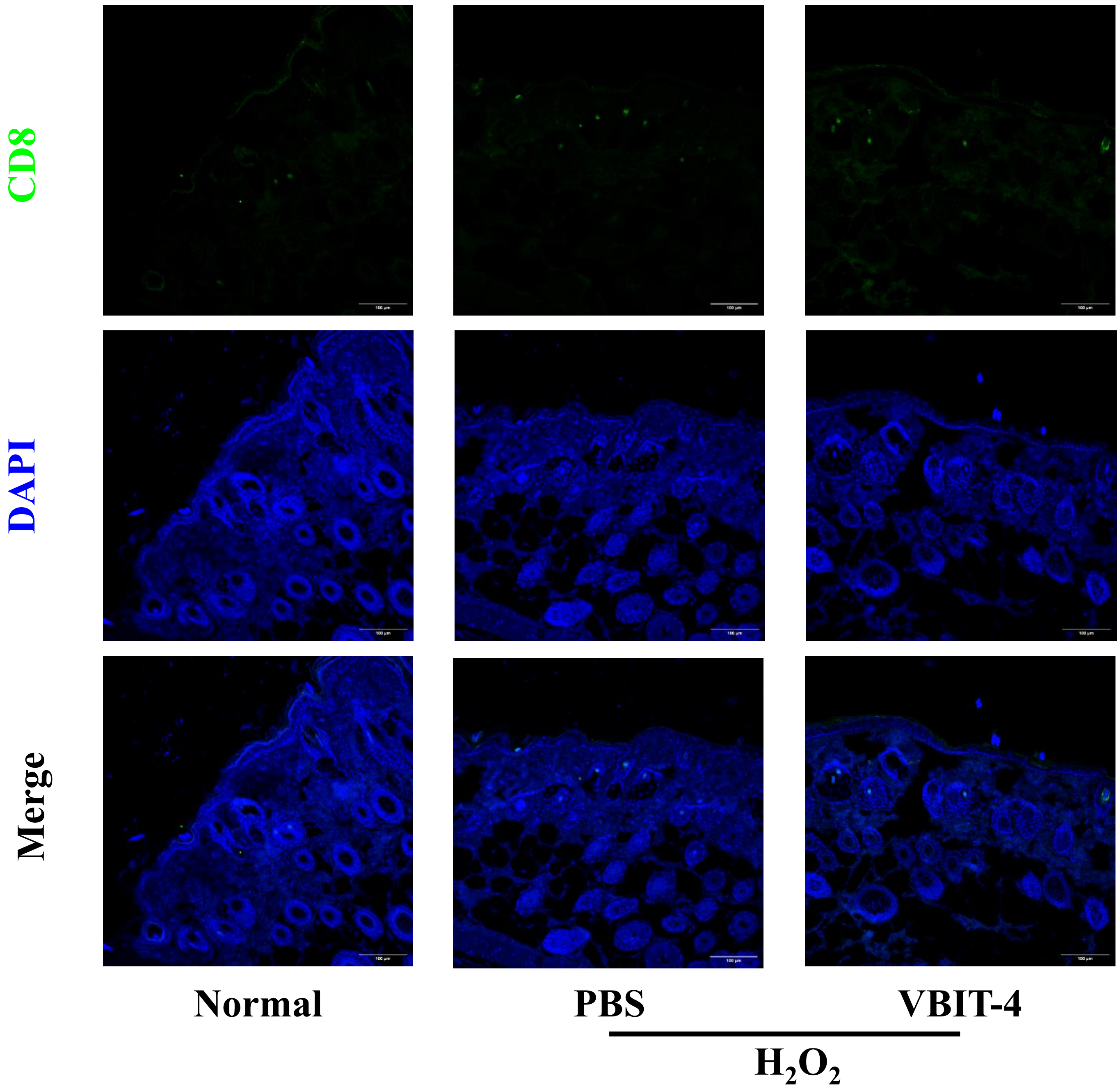

Figure 7D Repeat 3

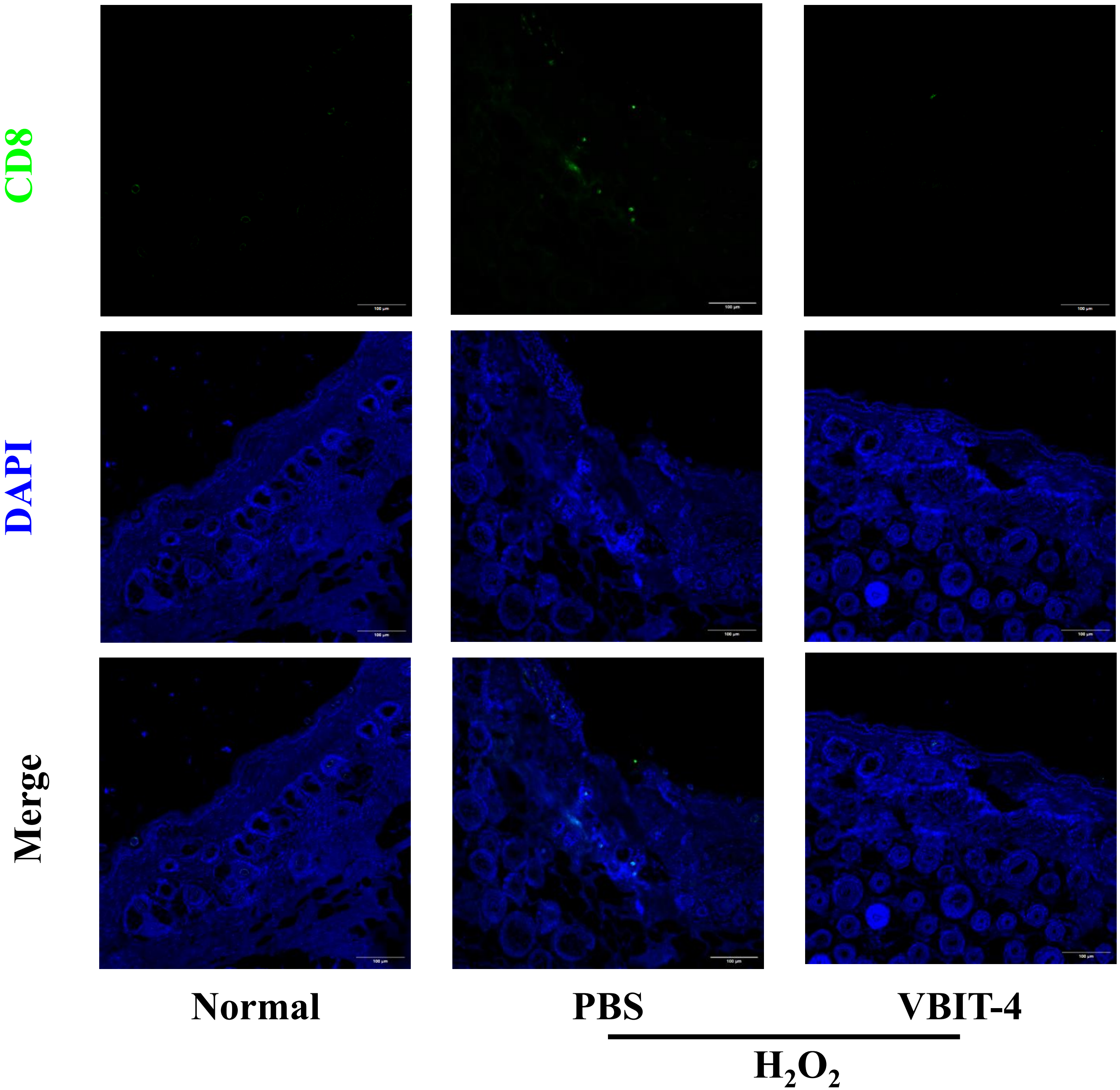

Figure S5A Repeat 1

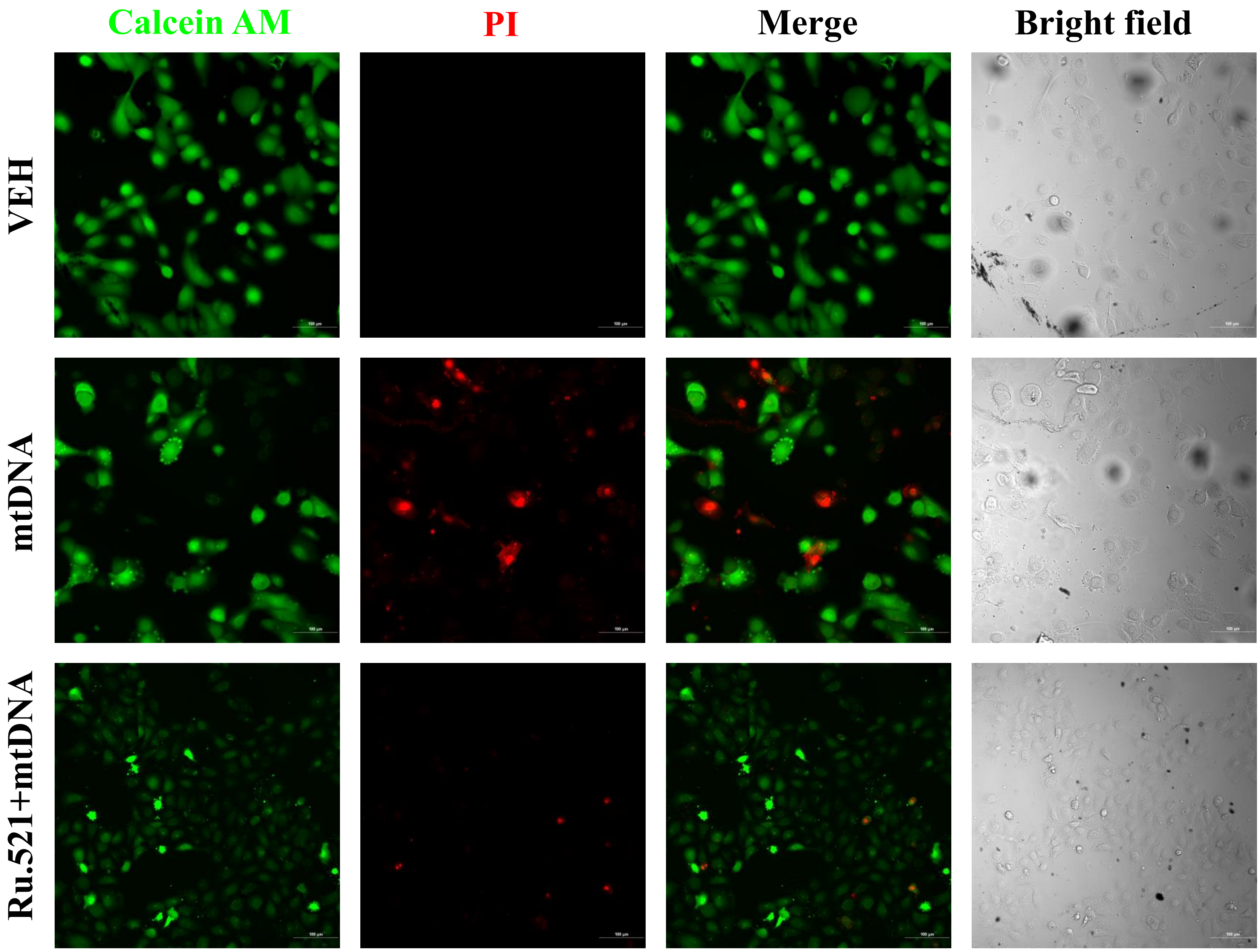

Figure S5A Repeat 2

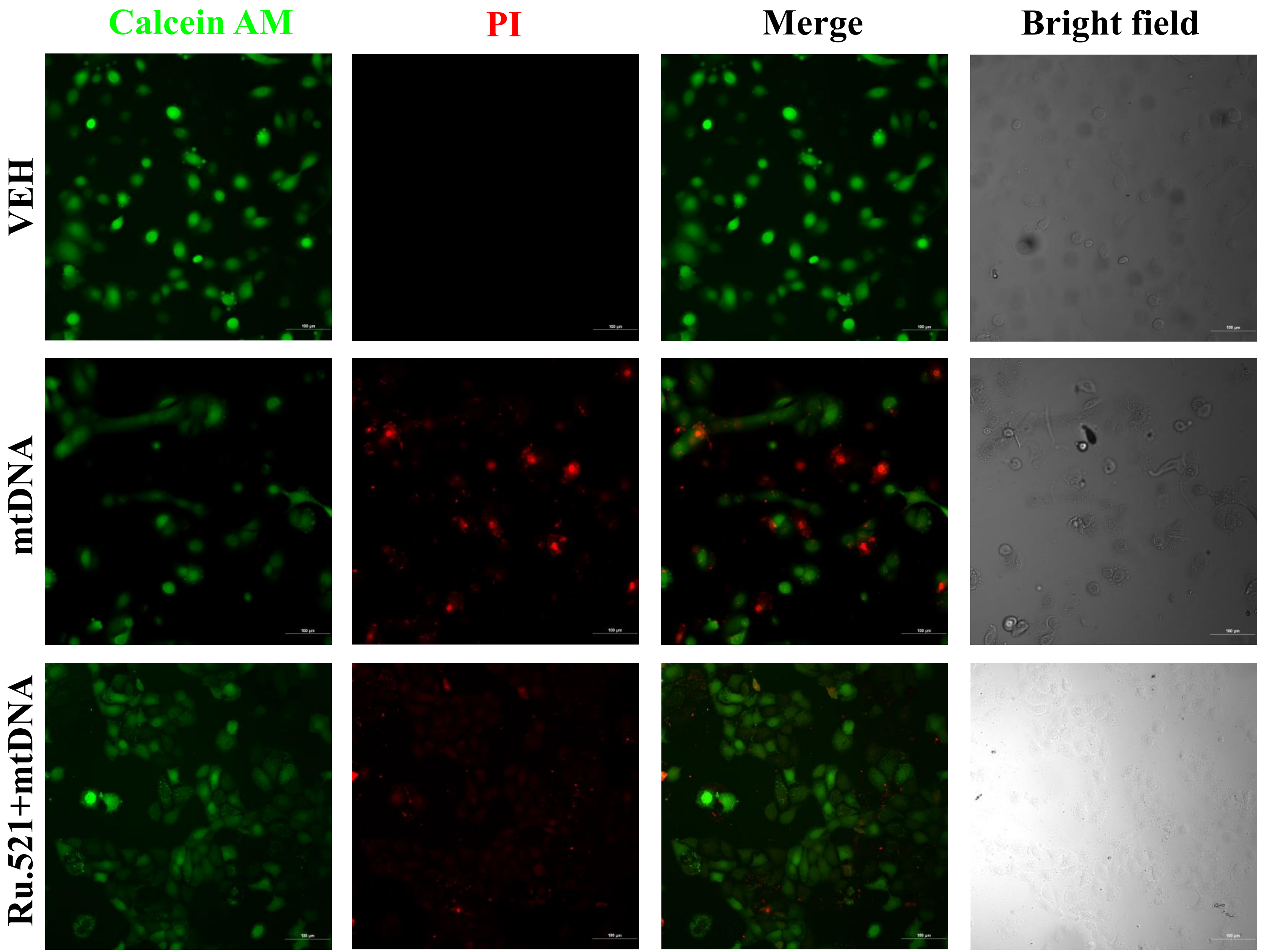

Figure S5A Repeat 3

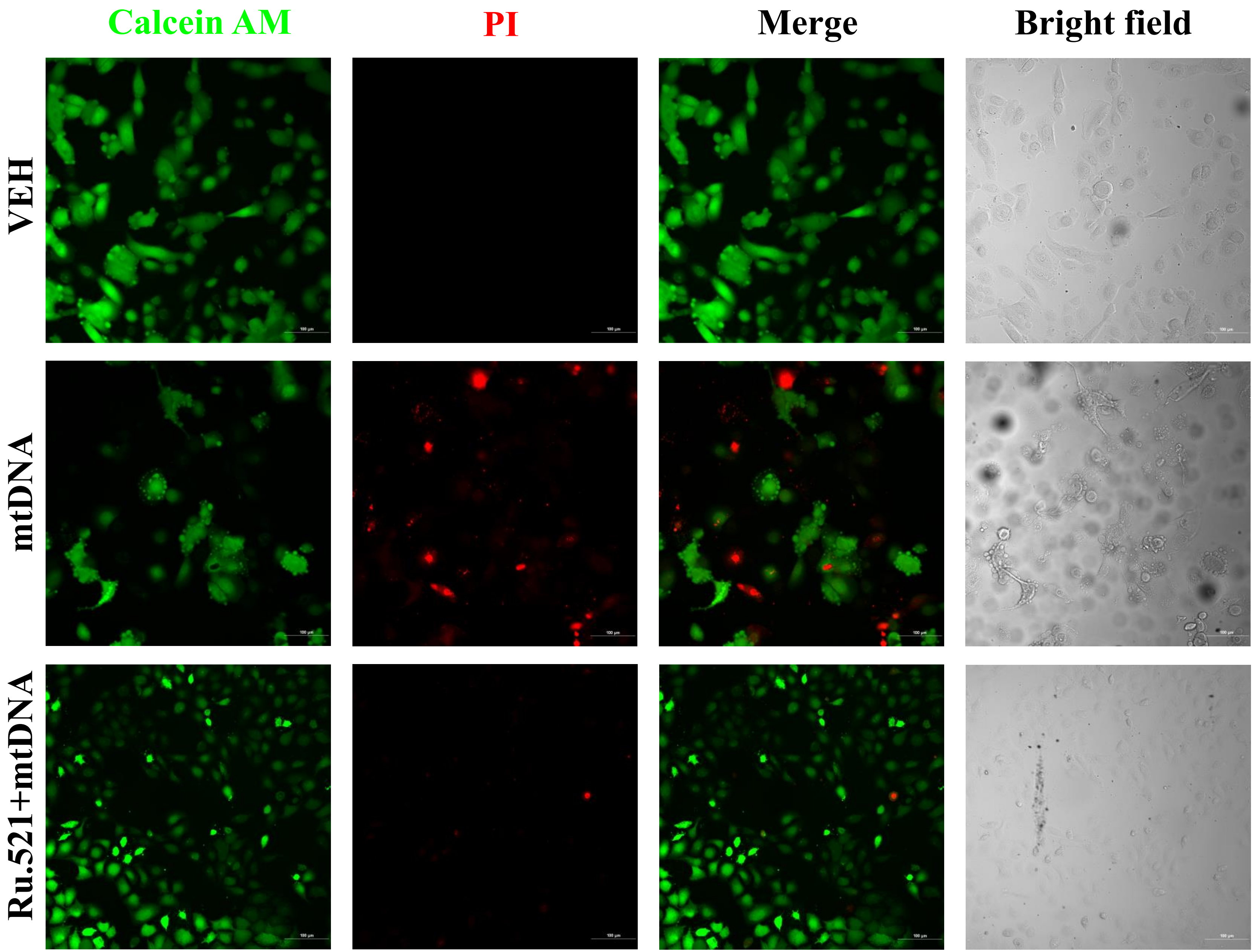

Figure S5B

Repeat 1		VEH	mtDNA	Ru.521+mtDNA
	PI	1	18	20
	Total	81	43	195
	%	1.23	41.86	10.26
**Repeat 2**		**VEH**	**mtDNA**	**Ru.521+mtDNA**
	PI	1	32	14
	Total	64	44	98
	%	1.56	72.73	14.29
**Repeat 3**		**VEH**	**mtDNA**	**Ru.521+mtDNA**
	PI	2	24	15
	Total	66	33	176
	%	3.03	72.73	8.52

Figure S6A

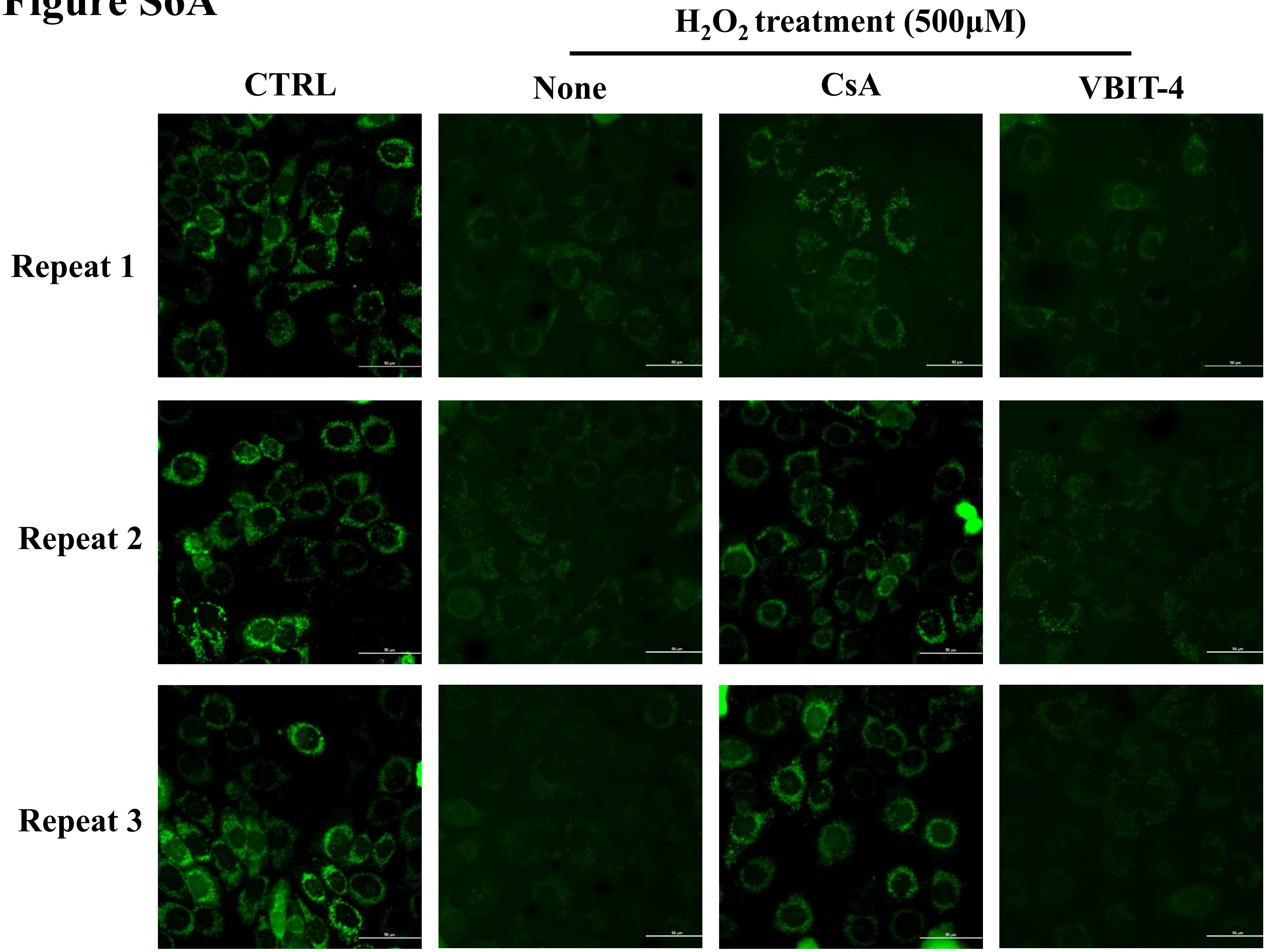

H_2O_2 treatment (500μM)
CTRL
None
CsA
VBIT-4
Repeat 1
Repeat 2
Repeat 3

Figure S6A

Repeat 1		CTRL	H_2O_2	CsA+H_2O_2	VBIT-4+H_2O_2
	Mean	78.398	36.321	61.483	41.924
	%	100.00	46.33	78.42	53.48
**Repeat 2**		**CTRL**	**H_2O_2**	**CsA+H_2O_2**	**VBIT-4+H_2O_2**
	Mean	102.683	37.556	77.743	48.545
	%	100.00	36.57	75.71	47.28
**Repeat 3**		**CTRL**	**H_2O_2**	**CsA+H_2O_2**	**VBIT-4+H_2O_2**
	Mean	96.004	33.469	86	43.77
	%	100.00	34.86	89.37	45.59
