# Supplementary material for: Mitochondrial DNA release via VDAC1 in keratinocytes: a key driver of innate immunity and vitiligo pathogenesis
Source: Cell Death Dis. 2026 Mar 18;17(1):318. doi: 10.1038/s41419-026-08585-5 (PMC13039960; doi:10.1038/s41419-026-08585-5)

FIG 5B

Repeat 1 Repeat 2 Repeat 3

CTRL

H_2O_2

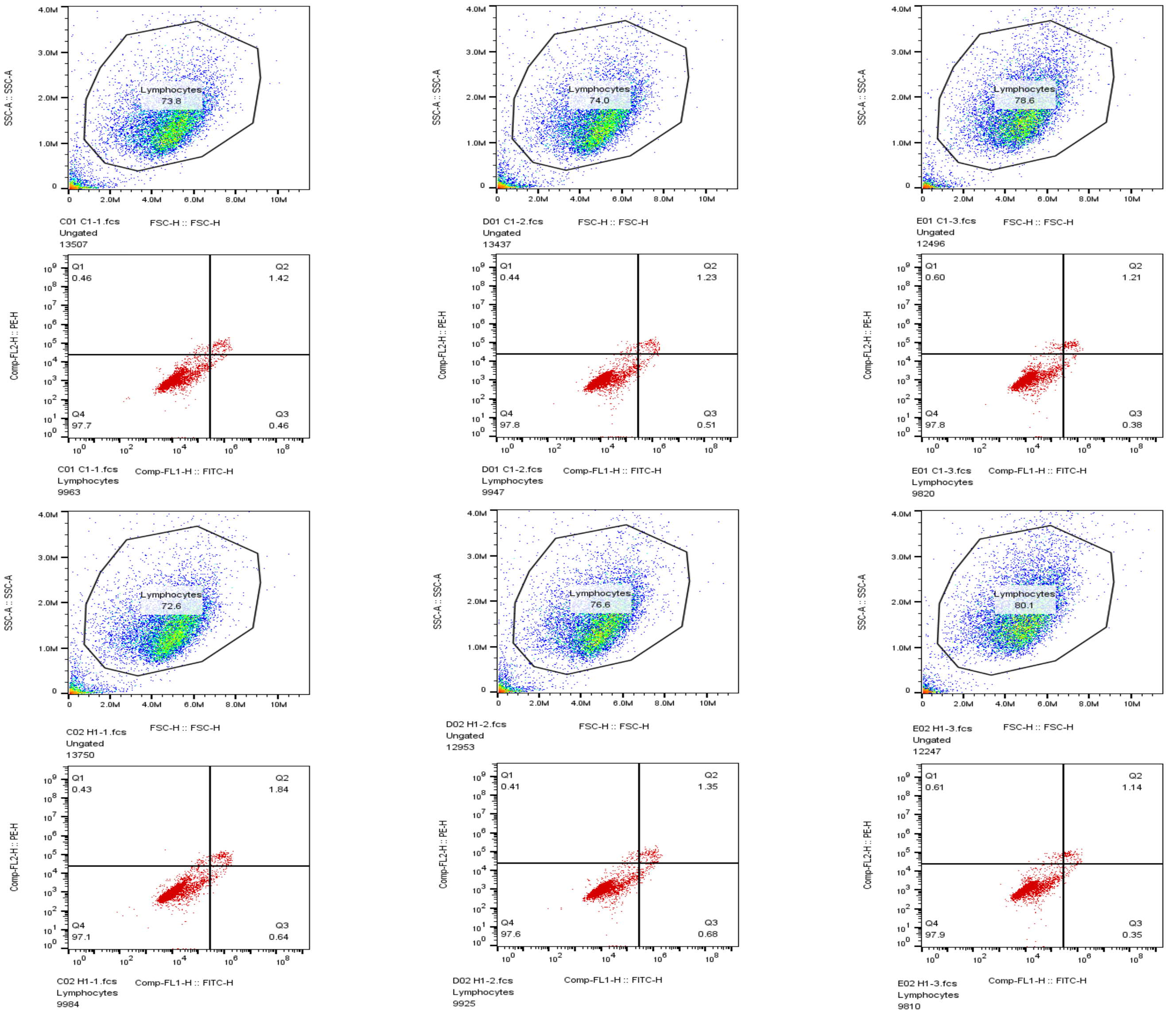

	C1	C2	C3
Q2	1. 42	1. 23	1. 21
Q3	0. 46	0. 51	0. 38
Q2+Q3	1. 88	1. 74	1. 59

	H1	H2	H3
Q2	1. 84	1. 35	1. 14
Q3	0. 64	0. 68	0. 35
Q2+Q3	2. 48	2. 03	1. 49

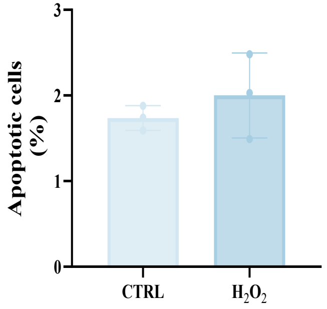

FIG S6B

Repeat 1 Repeat 2 Repeat 3

CTRL

H_2O_2

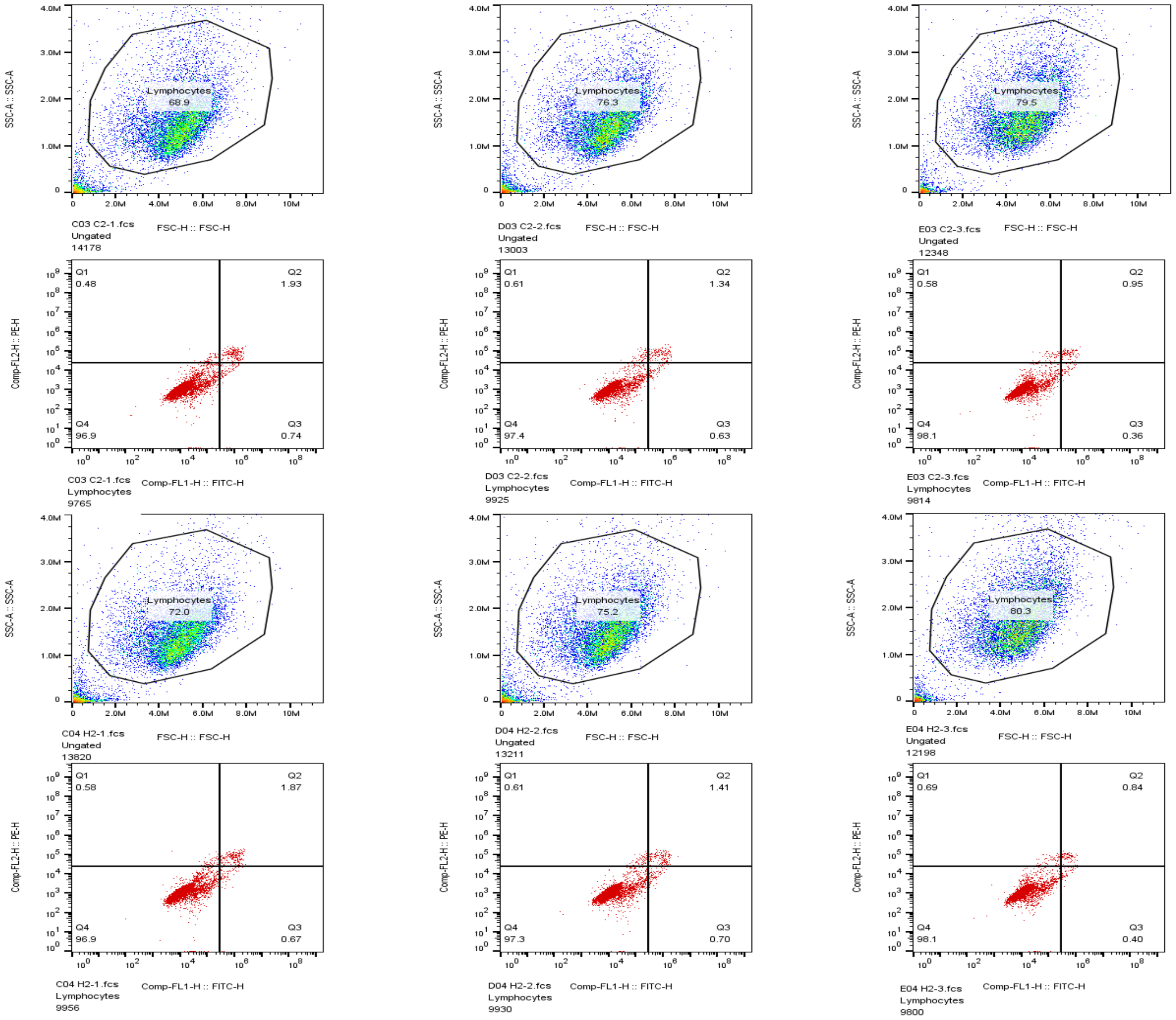

	C1	C2	C3
Q2	1.93	1.34	0.95
Q3	0.74	0.63	0.36
Q2+Q3	2.67	1.97	1.31

	H1	H2	H3
Q2	1.87	1.41	0.84
Q3	0.67	0.7	0.4
Q2+Q3	2.54	2.11	1.24

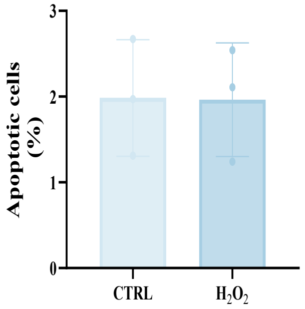

Supplement: Supplementary file 5 — Supplementary Data-Flow cytometry data [file 41419_2026_8585_MOESM5_ESM.pdf]
